# Gold-Based Coronands as Hosts for M^3+^ Metal Ions: Ring Size Matters

**DOI:** 10.3390/molecules28145421

**Published:** 2023-07-14

**Authors:** Suelen Ferreira Sucena, Türkan Ilgin Demirer, Anna Baitullina, Adelheid Hagenbach, Jacqueline Grewe, Sarah Spreckelmeyer, Juliane März, Astrid Barkleit, Pedro Ivo da Silva Maia, Hung Huy Nguyen, Ulrich Abram

**Affiliations:** 1 Institute of Chemistry and Biochemistry, Freie Universität Berlin, Fabeckstr. 34/36, 14195 Berlin, Germany; 2 Department of Nuclear Medicine and Berlin Institute of Health, Charité—Universitätsmedizin Berlin, Corporate Member of Freie Universität Berlin and Humboldt-Universität zu Berlin, Augustenburger Platz 1, 13353 Berlin, Germany; 3 Institute of Resource Ecology, Helmholtz-Zentrum Dresden-Rossendorf (HZDR), Bautzner Landstr. 400, 01328 Dresden, Germany; 4 Núcleo de Desenvolvimento de Compostos Bioativos (NDCBio), Universidade Federal do Triângulo Mineiro, Av. Dr. Randolfo Borges 1400, Uberaba 38025-440, MG, Brazil; 5 Department of Inorganic Chemistry, VNU University of Science, 19 Le Thanh Tong, Hanoi 100000, Vietnam

**Keywords:** coronands, gold, lanthanides, aroylthioureas, self-assembly

## Abstract

The controlled, self-assembled synthesis of multinuclear coordination compounds can be performed via different approaches. Frequently, steric, geometric and/or electronic factors located at the ligand systems predefine the way in which metal ions can assemble them to large aggregates. For the compounds in the present paper, also the Pearson’s acidities and preferred coordination geometries of the metal ions were used as organization principles. The ligand under study, 2,6-dipicolinoylbis(*N*,*N*-diethylthiourea), H_2_L1^ethyl^, possesses ‘soft’ sulfur and ‘hard’ nitrogen and oxygen donors. One-pot reactions of this compound with [AuCl(tht)] (tht = tetrahydrothiophene) and M^3+^ salts (M = Sc, Y, La, Ln, Ga, In) give products with gold-based {Au_3_(L1^ethyl^)_3_}^3+^ or {Au_2_(L1^ethyl^)_2_}^2+^ coronands, which host central M^3+^ ions. The formation of such units is templated by the M^3+^ ions and the individual size of the coronand rings is dependent on the ionic radii of the central ions in a way that small ions such as Ga^3+^ form a [Ga⊂{Au_2_(L1^ethyl^)_2_}]^+^ assembly, while larger ions (starting from Sc^3+^/In^3+^) establish neutral [M⊂{Au_3_(L1^ethyl^)_3_}] units with nine-coordinate central ions.

## 1. Introduction

Molecular self-assembly or molecular self-organization are common organization principles in all branches of chemistry and many supramolecular systems found in the biological chemistry or material sciences are products of such processes. The thermodynamic drive to lower energy is regarded as the general principle of such processes, which frequently work with a combination of strong and weak intra- and intermolecular interactions [1,2,3,4,5]. Similar mechanisms apply for metal-based compounds, where mainly coordinative bonds are responsible for the self-assembled formation of supramolecular aggregates. Such systems are of widespread interest in different fields of research, e.g., as molecular nanocontainers, molecular magnets, catalysts and as models (or even mimics) of reactive centers in bioinorganic systems [6,7,8,9,10,11,12,13,14,15,16].

Well-defined supramolecular aggregates are commonly obtained in one-pot reactions by mixing metal precursors and appropriate ligands in defined ratios and suitable solvents. A rational design of the desired products becomes possible by a careful selection of the ligand systems concerning their coordination abilities and inherent constraints defined by the arrangement of the donor atoms, their chelating abilities and/or by geometrical parameters, which control the possible coordination sphere(s) of the metal ion(s). Several successful approaches were reported, e.g., the directional binding approach by P. Stang and coworkers [17], Fujita’s molecular paneling approach [18], Raymond’s use of symmetry-triggered interactions [19], Mirkin’s weak-interaction approach [20], or the use of bimetallic building blocks as was demonstrated by Cotton and coworkers [21]. Well-defined supramolecular compounds with targeted shapes and sizes are accessible following these synthetic principles and two-dimensional assemblies such as molecular triangles or squares [22,23], but also three-dimensional units such as tetrahedral or octahedral cages were reported [24,25]. Similar organization principles are applicable for other large molecular aggregates as in the case of the compounds of the present paper.

**Figure 1:**
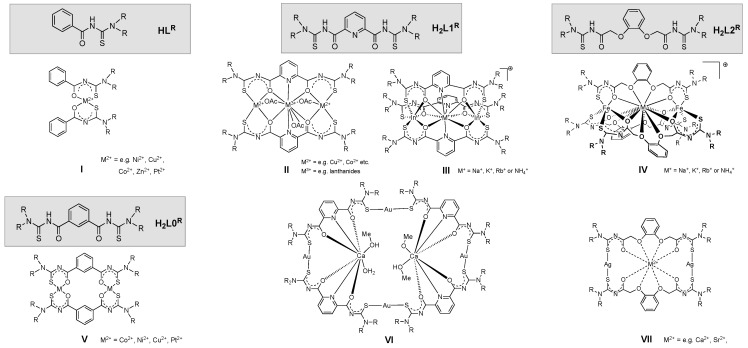
Mono- and bipodal aroyl-*N,N*-(dialkylthioureas) and their complexes [27,28,29,30].

In addition to topological aspects of the ligand design and preferred coordination environments of the used metal ions, also simple principles, e.g., Pearson’s classical concept of ‘hard and soft’ acids and bases [26], can be applied to direct metal ions to desired positions in multidentate ligands. This is particularly useful when ligands have ‘hard’ and ‘soft’ donor atoms well separated in different positions of their scaffold. Such ligands can be designed on the basis of aroylthioureas, which represent in their most simple form, the benzoylthioureas (HL^R^), a bidentate *S*,*O* chelator, which forms [M(L^R^)_2_] complexes (type **I** in Figure 1) with a large variety of transition metal ions [27,28,29,30]. It is interesting to note that corresponding bipodal phthaloylbis(*N*,*N*-dialkylthioureas) with two *S*,*O* chelating units form multinuclear assemblies with divalent metal ions such as Ni^2+^, Pt^2+^ or Cu^2+^. The nuclearity of the resulting complexes strongly depends on the relative positions of the aroylthiourea units. Trinuclear products are formed when two of the acylthiourea units substitute the central phenyl ring in para-positions, while meta substitution (ligands H_2_L0^R^ in Figure 1) result in dimeric products [31,32,33,34,35,36,37,38].

Although the bi- or trinuclear complexes of the composition [M^II^(*meta*-L0^R^)]_2_ (see Figure 1) or [M^II^(*para*-L0^R^)]_3_ [31,32,33] possess large central voids, these voids are empty and cannot accommodate any guest atoms or molecules. No suitable donor atoms are available and the effective space is restricted by the hydrogen atoms of the central phenyl rings pointing toward the centers. The replacement of the central phenyl ring in ligand H_2_L0^R^ by a pyridine ring or the introduction of ether units in the side chains results in the formation of the ligand systems H_2_L1^R^ and H_2_L2^R^, which have such donor atoms available.

Moreover, they represent a class of ligands with bifunctional coordination sites (moderately ‘soft’ *S*,*O* and ‘medium hard’ *O*,*N*,*O* or ‘hard’ *O*,*O*,*O*,*O* donors), each of which favorably binds to a particular type of metal ions. This gives access to the syntheses of a large variety of multinuclear, bimetallic complexes (types **II**, **II**, **IV**, **VI** and **VII** in Figure 1). They are usually formed by simple one-pot reactions in solutions containing the ligands and mixtures of two metal ions with different Pearson’s acidity [39,40,41,42,43,44,45,46,47]. Particularly interesting bonding features are established with ‘soft’ metal ions such as Ag^+^ or Au^+^, where no chelate formation was found, but a monodentate coordination to the sulfur atoms of the ligands is observed. As a result, macrocyclic, coronand-type ligands were formed, which can accommodate one or two alkaline earth metal ions [30,41]. The ready formation of such assemblies raises questions concerning the mechanism of their formation, the role of the (central) metal ion(s) and if the resulting ring size is exclusively determined by the used precursors H_2_L1^R^ or H_2_L2^R^.

In the present study, we report the results of reactions of the pyridine-based ligand H_2_L1^ethyl^, the common Au(I) precursor [AuCl(tht)] (tht = tetrahydrothiophene) and salts of M^3+^ ions. The trivalent metal ions were taken from group 3, group 13 of the periodic table and from the lanthanide series.

## 2. Results and Discussion

### 2.1. General Synthetic Aspects

**Scheme 1:**
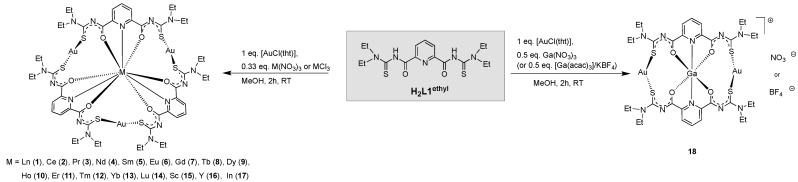
Syntheses of the [M⊂{Au_3_(L1^ethyl^)_3_}] complexes and [Ga⊂{Au_2_(L1^ethyl^)_2_}]^+^ salts.

One-pot reactions of each three equivalents of [AuCl(tht)] (tht = tetrahydrothiophene) and H_2_L1^ethyl^, and one equivalent of the chlorides or nitrates of the trivalent metal ions shown in the left part of Scheme 1 in methanol give tetranuclear complexes of the composition [M⊂{Au_3_(L1^ethyl^)_3_}]. The use of labile gold(I) starting material such as [AuCl(tht)] is essential for the isolation of the products in good yields and high purity. Although the use of (NEt_4_)[Au^III^Cl_4_] also results in the formation of [M ⊂ {Au_3_(L1^ethyl^)_3_}] compounds, the products obtained in this way contain a significant amount of less-defined side-products resulting from the reduction of the Au(III) precursor, which are hard to remove from the host/guest compounds. Attempted reactions with [AuCl(PPh_3_)] instead of [AuCl(tht)] did not form the mixed-metal assemblies, most probably due to the stability of the Au-P bond in this precursor (vide infra).

The selection of the M^3+^ precursors is not crucial as long as the used salts are sufficiently soluble in methanol. The use of the corresponding chlorides and nitrates is perfectly suitable and the anions do not have any influence on the obtained yields. Alternatively, also labile chelate complexes of the M^3+^ ions (e.g., acetylacetonates) can be used as precursors. This was proven with [Sc(acac)_3_] and [Ga(acac)_3_]. The corresponding one-pot reactions give pure products in similar yields as reactions starting from salts. For lanthanides, this approach was not attempted since the reactions starting from the nitrates or chlorides gave good yields and the products precipitated in high purity.

The [M⊂{Au_3_(L1^ethyl^)_3_}] complexes **1**–**17** precipitate directly from the reaction mixtures upon stirring the components at room temperature, either directly or after the addition of a few drops of triethylamine as a supporting base. For the isolation of [Ga⊂{Au_2_(L1^ethyl^)_2_}](BF_4_), which was prepared from [Ga(acac)_3_], the addition of one equivalent of KBF_4_ was required. The products are readily soluble in CH_2_Cl_2_ or CHCl_3_. Single crystals were commonly produced by slow diffusion of MeOH or diethyl ether into CH_2_Cl_2_ solutions of the complexes. The products were characterized by elemental analysis, IR and UV-vis spectroscopy, mass spectrometry and (where appropriate) by NMR spectroscopy.

The first information about the success of the reactions can be derived from two indicative features in the IR spectra of the products: (1) double deprotonation of the organic ligands can be concluded from the disappearance of the NH stretches of the ligands in the region above 3200 cm^−1^ and (2) the C=O bands, which appears at 1674 cm^−1^ in the uncoordinated H_2_L1^ethyl^ [42], are bathochromically shifted in the spectra of the complexes by approximately 100 cm^−1^. This is a clear sign for the formation of a delocalized π-system upon coordination. Stronger shifts by more than 120 cm^−1^ are observed when the aroylthiourea ligands are involved in *O*,*S*-chelate formation [47]. More structural and spectroscopic features of the complexes will be discussed within the group of metals in the following sections.

**Scheme 2:**
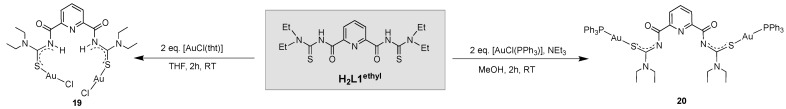
Syntheses of [(AuCl)_2_(H_2_L1^ethyl^)] (**19**) and [{Au(PPh_3_)_2_(L1^ethyl^)}] (**20**).

The formation of large ring systems with two, three or four [30] {Au(L1^ethyl^)}^−^ units is not intuitive and requires an explanation. As already mentioned above, the selection of reactive gold starting materials is important and the use of [AuCl(tht)] with an only weakly bonded thioether ligand proved to be optimal. Corresponding one-pot reactions between H_2_L1^ethyl^, [AuCl(PPh_3_)] and M^3+^ ions gave only mixtures of poorly defined products. Therefore, we performed reactions of H_2_L1^ethyl^ with [AuCl(tht)] or [AuCl(PPh_3_)] in absence of additional metal ions (Scheme 2).

Such reactions gave pure products of the composition [(AuCl)_2_(H_2_L1^ethyl^)] (**19**) and [{Au(PPh_3_)}_2_(L1^ethyl^)] (**20**). They precipitate as colorless solids from the reaction mixtures either after a short heating period (**19**) or after the addition of NEt_3_ (**20**). The necessity of a supporting base for the latter reaction arises from the required deprotonation of the ligand, which is not the case during the formation of the chlorido complex **19**. It becomes evident that in both reactions only the sulfur atoms of 2,6-dipicolinoylbis(*N*,*N*-diethylthiourea) are involved in the coordination and neutral, linear gold(I) complexes are formed. Charge compensation in complex **20** is achieved by a double deprotonation of H_2_L1^ethyl^, which can readily be seen in the IR spectrum of the compound, where the ν(NH) stretch (3302 cm^−1^ in **19**) is absent. The ^1^H NMR spectrum of **19** provides additional evidence for the proposed composition. It shows a singlet related to the NH proton at 10.83 ppm. One doublet and one triplet at 8.42 and 8.20 ppm are assigned to the protons of the pyridine ring. Two quartets due to the CH_2_ protons (4.00 and 3.75 ppm) and two triplets of the CH_3_ protons (1.49 and 1.42 ppm) show that the ethyl groups are magnetically not equivalent.

The ESI+ mass spectrum of **19** does not show the molecular ion and the most intense signal belongs to the [Au(H_2_L1^ethyl^)]^+^ cation at *m*/*z* = 592.1175 (calcd. 592.1110). An intense signal at *m*/*z* = 1334.2305 in the spectrum of compound **20** can be assigned to the corresponding [M+Na]^+^ ion (calcd. 1334.2339). In none of the two spectra was evidence found for signals, which can be interpreted for the formation of {Au_2_(L1^ethyl^)_2_}^2−^, {Au_3_(L1^ethyl^)_3_}^3−^ or {Au_4_(L1^ethyl^)_4_}^4−^ rings.

**Figure 2:**
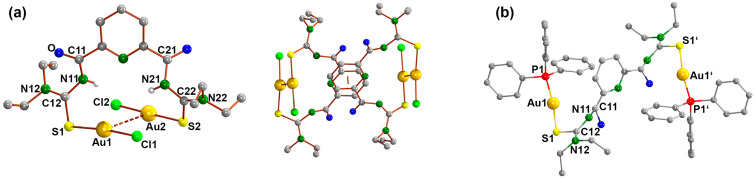
Molecular structures of (**a**) [(AuCl)_2_(H_2_L^ethyl^)] (**19**) and (**b**) [{Au(PPh_3_)}_2_(L1^ethyl^)] (**20**).

The spectroscopic information could be confirmed by single-crystal X-ray diffraction studies on [(AuCl)_2_(H_2_L1^ethyl^)] (**19**) and [{Au(PPh_3_)}_2_(L1^ethyl^)] (**20**). Figure 2 depicts the molecular structures of both compounds. Although the H_2_L1^ethyl^ or {L1^ethyl^}^−^ ligands are only bonded to gold via their sulfur atoms, the coordination results in some bond length modifications in the skeleton of the ligands with regard to the non-coordinated form. The corresponding values are summarized in Table 1. The values clearly show that the C–S bonds are lengthened upon coordination irrespective of the deprotonation of the ligand. Furthermore, a considerable bond length equalization is observed for the C–N bonds. This is a common result for this type of ligands and is explained by the formation of an extended π-system as the consequence of the chelate formation [27,28,29]. In the present case, it is found for monodentate coordinated aroylthioureas as well as for non-deprotonated ligands of this type.

The Au-Cl and Au-S distances in compound **19** are very similar to those observed in the structure of *N*,*N*-diethyl-*N′*-camphanylthioureatogold(I) chloride obtained by Koch [48] and *N*,*N*-diethyl-*N′*-benzoylthioureatogold(I) chloride reported by Bensch [49]. Weak Au–Au interactions of 3.5493(5) Å are established in the solid-state structure of **19**. They are accompanied by π–π interactions between the pyridine rings of two adjacent molecules of 3.709(1) Å as is illustrated in Figure 2a. The values of the Au···Au as well as the offset parallel orientation of the π–π interactions are consistent with the values determined by Pathaneni [50] and Janiak [51], respectively. The shortest Au–Au distance in compound **20** is 9.535(8) Å. Thus, no bonding interactions can be discussed. The solid state structure of this compound exhibits a perfect disorder of the carbonyl groups and pyridine ring over two positions. A similar disorder is observed in another gold(I) complex with an isophtaloylbis(thiourea) ligand [52]. As in the chlorido complex **19**, the gold atoms in **20** are in an almost linear environment with a P–Au–S angle of 171.4(1)°. The Au–P distances of 2.267(3) Å and the Au-S bond lengths of 2.296(3) Å are similar to the values in the structures published by Schwade [52] and Gimeno [53].

The ready formation of [(AuCl)_2_(H_2_L^ethyl^)] (**19**) during the reaction of [AuCl(tht)] with H_2_L1^ethyl^ without any sign for the parallel formation of cyclic units as well as the dependence of the obtained ring sizes on the template ion (Scheme 1 and [30]) strongly indicate a mechanism involving templating for the formation of the title compounds.

### 2.2. Lanthanum and the Lanthanide Series

Chlorides or nitrates of the lanthanide elements readily undergo the reactions illustrated in Scheme 1 and described above. The resulting [M⊂{Au_3_(L1^ethyl^)_3_}] complexes precipitate from the reaction mixtures essentially as colorless solids. Only the cerium complex has an orange-red color, while the holmium one is pale pink. The IR spectra of the products show the features described in the previous section and allow for a fast assessment of the colorless solids obtained.

NMR spectra of sufficient quality were recorded for the diamagnetic lanthanum complex. Deprotonation of the organic ligand is indicated by the absence of NH signals, which are found in the spectrum of uncoordinated H_2_L1^ethyl^ at 9 ppm. The protons of the methylene groups are not equivalent and appear as three multiplets at 3.7, 3.5 and 3.4 ppm. This non-equivalence is due to the rigidity of the formed assemblies and hindered rotation around the C-NEt_2_ bonds. Similar effects were found for other complexes with {L1^ethyl^}^2−^ ligands [39,40,41,42,43], but are also typical for chelate complexes with the parent benzoylthioureato ligands, where a rotational barrier of approximately 650 kJ/mol was found [54]. The ^13^C NMR spectrum shows the signals in the expected regions. The C=O and C=S signals appear in the complexes at 185.5 and 159.3 ppm, while they are found in the spectrum of H_2_L1^ethyl^ at 177.2 and 156.3 ppm, respectively. The observed downfield shift of the signals upon coordination is not unusual for the class of ligands under study.

**Figure 3:**
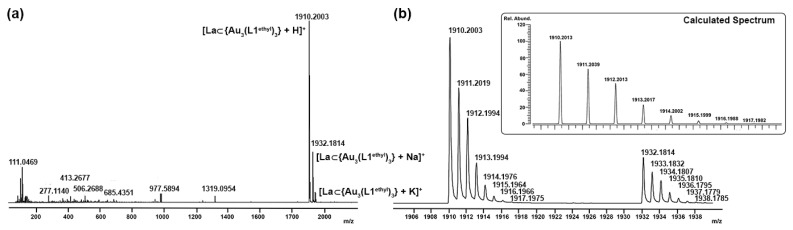
(**a**) Overview ESI+ mass spectrum of [La⊂{Au_3_(L1^ethyl^)_3_}] and (**b**) zoom of the molecular ion region together with the simulated masses.

An indicative method for the characterization of the complexes is mass spectrometry. ESI+ mass spectra of the host/guest complexes show the molecular peaks of the bimetallic [M⊂{Au_3_(L1^ethyl^)_3_} + H]^+^ assemblies with high intensity. They are frequently accompanied by the corresponding [M⊂{Au_3_(L1^ethyl^)_3_} + Na]^+^ and/or [M⊂{Au_3_(L1^ethyl^)_3_} + K]^+^ cluster ions. Figure 3 shows the spectrum of the lanthanum complex together with the simulation of the isotopic pattern of the molecular ion, which perfectly matches the experimental one. The mass spectra of the other compounds can be found in the Supplementary Materials. It is interesting to note that in almost all spectra of such compounds, fragments of low intensities are observed, which can be assigned to a subsequent loss of {Au(L1^ethyl^} units, while the lanthanide ions remain bonded. This type of fragmentation is doubtlessly attributed to the high-energy conditions inside the mass spectrometer, but might be regarded as a hint for general degradation pathways for such compounds under thermal conditions.

**Figure 4:**
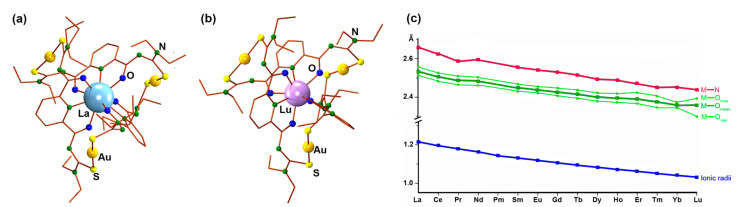
Molecular structures of (**a**) [La⊂{Au_3_(L1^ethyl^)_3_}] and (**b**) [Lu⊂{Au_3_(L1^ethyl^)_3_}]. The radii of the La^3+^ and Lu^3+^ ions are taken from ref. [55], the other atoms are shown with arbitrary sizes (Au, S, N, O) or as wireframe. (**c**) Graphical representation of the experimental Ln-N and Ln-O bond lengths for the entire series of nine-coordinate [Ln⊂{Au_3_(L1^ethyl^)_3_}] coronands together with the respective ionic radii of the M^3+^ ions taken from ref. [55]. For the Ln-O bonds each the minimum, mean and maximum values are given for the individual compounds.

The availability of a large number of lanthanide complexes with a presumably similar or identical composition makes it interesting to study the solid-state structures of the compounds. Therefore, we attempted to grow single crystals of the products for an X-ray study. Eventually, we obtained single crystals for almost the entire series with the exception of the complex with the radioactive promethium. The compounds could be crystallized by a careful covering of CH_2_Cl_2_ solutions with *n*-hexane and subsequent diffusion of the solvents. Irrespective of the central M^3+^ ion, the compounds have a composition of [M⊂{Au_3_(L1^ethyl^)_3_}] with a coordination number of nine for the central M^3+^ ions. This is remarkable considering they encompass ions with radii ranging from 1.216 Å (La^3+^) to 1.032 Å (Lu^3+^) [55]. Figure 4 shows the molecular structures of the lanthanum and lutetium complexes as representatives of the full series. The La^3+^ and Lu^3+^ ions are depicted with their respective ionic radii and it becomes evident that the sizes of these ions do not have a significant influence on how they are hosted by the {Au_3_(L1^ethyl^)_3_}^3−^ coronands. This is a clear sign that the 36-membered {Au_3_(L1^ethyl^)_3_}^3−^ ring system is flexible over a wide range for the coordination of central metal ions. The structures of the other compounds are similar and are depicted in the Supplementary Materials.

Although the coordination environments of the central M^3+^ ions in the [M⊂{Au_3_(L1^ethyl^)_3_}] complexes seem to be independent of their ionic radii, clear changes in the M-N and M-O bond lengths are found. They decrease almost steadily from the lanthanum to the lutetium compound, following the well-documented lanthanide contraction as is shown in Figure 4c, where they are depicted along with the radii of the central ions for coordination number nine [55]. A summary of selected bond lengths and angles is given in Table 2. Some anomalies become evident with the three elements with the smallest ionic radii (thulium, ytterbium and lutetium), where differences between the individual M-O bond lengths are apparently larger (Tm, Lu) or smaller (Yb) than in the other compounds. On the basis of the available data, however, it is not justified to attribute a single reason to this observation, especially considering that unlike all other [M⊂{Au_3_(L1^ethyl^)_3_}] complexes in this series, the thulium and lutetium compounds do not crystallize in the trigonal space group $P\bar{3}$. The M-N bonds are not affected to the same extent.

It is also interesting to note that the M^3+^–O and M^3+^–N bond lengths in the gold-containing coronates of the present study are remarkably short compared with another series of nine-coordinate lanthanide chelates with {L1^ethyl^}^2−^ ligands: trinuclear cobalt(II) complexes of the composition [Co_2_Ln(L1^ethyl^)_2_(µ-acetate)_2_Cl] with Ln = Ce, Nd, Sm, Dy, Er, Yb [43]. The M^3+^ ions in the latter compounds are bonded by the *N* and *O* donor atoms of each two {L1^ethyl^}^2−^chelators and additional acetato and chlorido ligands (see compounds II in Figure 1). Their M^3+^–N bonds also regularly decrease within the lanthanides series, but span a range from 2.650(3) Å (Ce^3+^) to 2.541(3) Å (Yb^3+^). The corresponding M^3+^–(L1^ethyl^)O bonds range between 2.606(3) and 2.359(3) Å. For La^3+^ and Gd^3+^, the coordination spheres are widened and compounds with the coordination number ten are formed [43]. This is not observed for the [M⊂{Au_3_(L1^ethyl^)_3_}] complexes of the present study supporting the indicated high flexibility of the donor atom constellation in the {Au_3_(L1^ethyl^)_3_}^3−^ coronands. Geometric strains seem to play an increasing role, however, for the [M⊂{Au_3_(L1^ethyl^)_3_}] complexes when the M^3+^ ions become smaller. The first effects are observed for the thulium, ytterbium and lutetium compounds (see Figure 4). More consequences of a further decrease in the radii of the central ions become evident for the group 3 and group 13 elements (vide infra), where a clear influence on the coordination sphere of the metal ions and on the reactivity of the products is observed.

**Figure 5:**
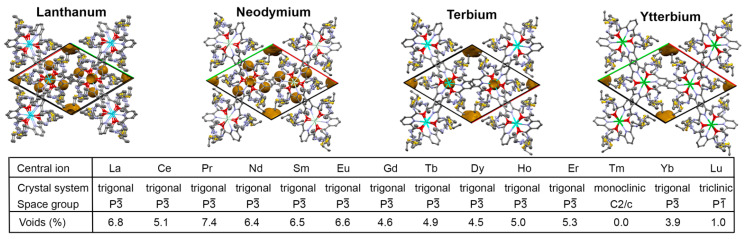
Plots of the trigonal unit cells of [La⊂{Au_3_(L1^ethyl^)_3_}], [Nd⊂{Au_3_(L1^ethyl^)_3_}], [Tb⊂{Au_3_(L1^ethyl^)_3_}] and [Yb⊂{Au_3_(L1^ethyl^)_3_}] together with solvent accessible voids. The values in the table below represent voids on the basis of a probe radius of 1.2 Å calculated with *Mercury* [56].

Although the [M⊂{Au_3_(L1^ethyl^)_3_}] units are large assemblies, they are relatively compact and closely packed. This is illustrated in Figure 5, where the voids between the units are shown for four representative trigonal structures. They form channels along the *c* axis around the four edges of the unit cells, which predominantly host solvent molecules (mostly diffuse water) in most of the compounds. Additional smaller voids are established in the compounds with the larger M^3+^ ions. In general, the established voids do not exceed seven per cent of the unit cell volumes. [Tm⊂{Au_3_(L1^ethyl^)_3_}] and [Lu⊂{Au_3_(L1^ethyl^)_3_}], which crystallize in monoclinic or triclinic space groups are even tighter packed (see the listing of the involved percentage included in Figure 5).

Considerable progress in the exploration of the fundamental chemistry of lanthanides was achieved in recent years [57,58,59,60], and specific properties of their compounds make them interesting for applications particularly in fields such as catalysis [61,62,63,64,65,66,67,68,69,70], optical [71,72,73,74] and magnetic materials [75,76,77], but also in pharmacy and life science [78,79,80,81]. Although the present series of compounds would give a unique opportunity for a comprehensive comparative study, it cannot be the aim of the present, introducing paper to cover all these interesting issues of lanthanide chemistry. Some magnetic properties of lanthanide/transition metal complexes containing {L1^ethyl^}^2−^ ligands were published recently, showing clear differences in the temperature dependence of the magnetic susceptibility for the different lanthanide ions [43]. Gold(I) and gold(III) complexes found increasing interest in medicinal chemistry for cancer treatment [82,83,84,85,86,87] and, recently, the potential use of self-assembled host-guest compounds was addressed for such applications [87,88,89,90]. Consequently, also the bimetallic [M⊂{Au_3_(L1^ethyl^)_3_}] complexes of the present study may have a considerable potential for such purposes, particularly when it succeeds to increase the cytotoxic activity of the gold compounds either through cytotoxic effects induced by the guest metal ions or by ionizing radiation of appropriate β^−^-emitting isotopes (e.g., ^198^Au, ^177^Lu, ^90^Y, ^153^Sm, ^149^Tb). Suitable radioactive gamma or positron emitters (e.g., ^44^Sc, ^68^Ga, ^111^In) can also be used to monitor the therapeutic progress. The first studies on the evaluation of [M⊂{Au_3_(L1^R^)_3_}] complexes are currently underway [91] and shall not be treated more in detail in the present communication. Instead, we briefly want to describe exemplarily the luminescent properties of the europium compound.

**Figure 6:**
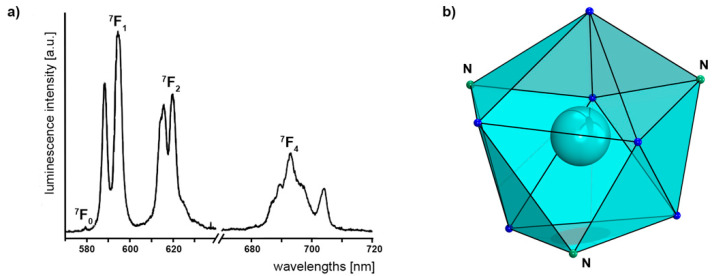
(**a**) Emission spectrum of [Eu⊂{Au_3_(L1^ethyl^)_3_}] recorded at an excitation wavelength 396 nm with 1200 mm^−1^ grating and (**b**) the coordination polyhedron of the nine-coordinate Eu^3+^ ion lying between a capped square antiprism (JCSAPR-9, CSAPR-9), tricapped trigonal prism (JTCTPR-9, TCTPR-9) and a muffin (MFF-9) [94].

In a recent review, the fluorescence properties of Eu^3+^ complexes were discussed with regard to the influence of the site symmetry in the compounds and used to derive structural information about the coordination geometry around the metal ion [92]. It is interesting to compare the derived information with the results of the single-crystal X-ray structure of the compound. In order to reach the necessary resolution of the single transitions, a grating of 1200 mm^−1^ was chosen. Figure 6a shows the emission spectrum of [Eu⊂{Au_3_(L1^ethyl^)_3_}] measured in CH_2_Cl_2_ at room temperature with a grating of 1200 mm^−1^. The europium(III) spectrum is composed of the red luminescence of the Eu^3+^ ion, which results from transitions from the first excited state (^5^D_0_) to all the lower J levels of the ground term ^7^F*_J_* (J = 0–4) [93]. Emission bands can be observed at 16835, 16207, 15290 and 14205 cm^−1^, which are attributed to the f-f transitions ^5^D_0_→^7^F_1_, ^5^D_0_→^7^F_2,_ ^5^D_0_→^7^F_3_ and ^5^D_0_→^7^F_4_, respectively.

An additional band is observed in the region of the ^5^D_0_→^7^F_0_ transition, but its intensity is very low and, thus, this transition can be regarded as forbidden. Transitions arising from ^5^D_1_ are not observed or they are too weak for an interpretation.

In the first and most intense transition, ^5^D_0_→^7^F_1_, the selection rules forbid an electric-dipole, but allow a magnetic-dipole transition [95]. It consists of two strong lines, where the lower frequency is split into two components. The ^5^D_0_→^7^F_2_ transition is a so-called “hypersensitive transition”, the intensity of which is markedly influenced by the environment of the europium(III) ion and the nature of the ligand [96]. In this case, we observed a relatively intense band with the higher frequency being split into two lines. A shoulder is observed in the second one. Its high intensity can be attributed to a low symmetry around the Eu^3+^ ion or the presence of highly polarizable ligands. Similar effects were found before, especially in compounds with chelating rings, such as β-diketonates or 2,6-pyridinedicarboxylato ligands [96], which are similar to H_2_L1^ethyl^.

No band is observed in the region of ^5^D_0_→^7^F_3_ transitions, while two broad bands of medium intensity appear in the region of the ^5^D_0_→^7^F_4_ transition. The highest frequency band is split into five lines, although it is quite difficult to determine their exact positions. The second one is split into two lines.

The derived relatively low symmetry of the Eu^3+^ ion in [M⊂{Au_3_(L1^ethyl^)_3_}] is in accordance with the experimental X-ray data. An analysis of the coordination polyhedron around europium with the continuous shape measures algorithm [97,98] gives no clear preference of one of the idealized coordination polyhedra for coordination number nine [94]. The bonding situation in the solid state can best be described as lying in between a capped square antiprism, a tricapped trigonal prism and a muffin structure, and a fluxional behavior in solution cannot be ruled out. A more detailed, comparative evaluation of the coordination polyhedra for the entire series of nine-coordinate [M⊂{Au_3_(L1^ethyl^)_3_}] complexes will be given vide infra.

### 2.3. Group 3 and Group 13 Elements

The formation of isostructural nine-coordinate compounds having the same donor atom constellation, which are recruited from one single, self-assembled ring system over the entire lanthanide series is unique. It became evident that the formation of such aggregates proceeds via a templated reaction, which requires effective interactions between the donor atoms and the central M^3+^ ions during their formation. The bond length values of Table 2 and particularly some anomalies found for the smaller ions suggest that the size of the M^3+^ ion seems to be important for the optimization of the metal–donor atom interactions. This raises the questions of the limiting factors for the formation of such nine-coordinate assemblies and what happens when [M⊂{Au_3_(L1^ethyl^)_3_}] complexes can no longer be stabilized. The latter question also becomes interesting in the light of the fact that divalent metal ions such as Ca^2+^ or Ba^2+^ form large {Au_4_(L1^ethyl^)_4_}^4−^ rings, which can accommodate two alkaline earth ions [30]. Good candidates to answer these questions might be given with the ions of the group 3 and group 13 elements having ion radii smaller than that of lutetium: e.g., Sc^3+^ (0.87 Å for coordination number C.N. 8), In^3+^ (0.92 Å for C.N. 8), Ga^3+^ (0.62 Å for C.N. 6). Additionally, the group 3 element yttrium with an ionic radius of 1.075 Å for C.N. 9 seem to be a good supplement to the values obtained for the lanthanides series given in Figure 2c and Table 1. Indeed, the bonding situation in [Y⊂{Au_3_(L1^ethyl^)_3_}] (see Table 3) is very similar to those in the lanthanides compounds with ionic radii close to that of Y^3+^ (Ho^3+^: 1.072 Å and Dy^3+^: 1.082 Å).

On the other hand, the very small Ga^3+^ ion (unlike the other M^3+^ ions regarded in this study) was not able to establish bonds with the nine donor atoms provided by a {Au_3_(L1^ethyl^)_3_}^3−^ ring system. Such a result is not completely surprising, considering the fact that the gallium complexes with coordination numbers larger than six are extremely rare. Apart from compounds with π-bonded rings, carbaboranes and cluster compounds, there are only a few entries of such compounds in the Cambridge Structural Database [99]. These examples involve polymeric carboxylates [100,101], crown ethers or cryptands [102]. It should be mentioned that the latter compounds contain gallium in its oxidation state “+1”. Reactions of common Ga(III) starting materials (Ga(NO_3_)_3_ or [Ga(acac)_3_], Hacac = acetylacetone) with mixtures of H_2_L1^ethyl^ and [AuCl(tht)] in methanol or THF give the six-coordinate [Ga⊂{Au_2_(L1^ethyl^)_2_}]^+^ cation (**18**) in good yields (Scheme 1). [Ga⊂{Au_2_(L1^ethyl^)_2_}]^+^ is the only charged product observed in all conducted reactions of metal ions with H_2_L1^ethyl^/[AuCl(tht)] mixtures, including those with Ca^2+^ and Ba^2+^ ions [30]. This suggests that the energetically favored interaction for the Ga^3+^ ions involves “chelate formation” with the 24-membered {Au_2_(L1^ethyl^)_2_}^2−^ ring system, rather than charge compensation with ligands such as nitrate.

**Figure 7:**
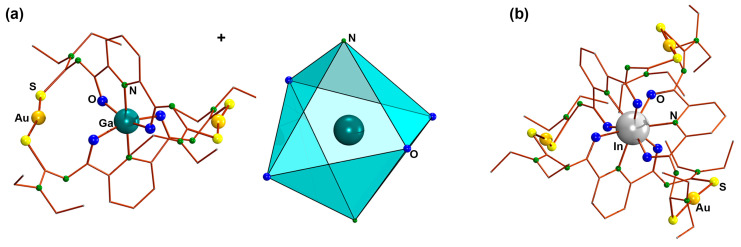
(**a**) Structure of the [Ga⊂{Au_2_(L1^ethyl^)_2_}]^+^ cation in **18a** together with the coordination polyhedron around the gallium ion and (**b**) the molecular structure of the [In⊂{Au_3_(L1^ethyl^)_3_}]. The size of the Ga^3+^ and In^3+^ ions are adopted from their ionic radii given in [55], the Au, S, P and N atoms are shown with arbitrary sizes.

Crystalline materials with nitrate or BF_4_^−^ counter ions were obtained directly from the reaction mixture in the case of [Ga⊂{Au_2_(L1^ethyl^)_2_}](NO_3_) (**18a**) or after the addition of KBF_4_ to the reaction mixture with [Ga(acac)_3_] (**18b**). The structures of the complex cations are not influenced by their counter ions and the structure of the nitrate salt is shown in Figure 7. Selected bond lengths and angles for both salts of the six-coordinate gallium complex are summarized in Table 3 along with the values of the scandium, yttrium and indium complexes, all of which are all nine-coordinate. The coordination polyhedron around the Ga^3+^ ion is a strongly distorted octahedron with the two pyridine nitrogen atoms in *trans* position to each other. The deviations from an ideal octahedral symmetry are evident by *cis*-angles ranging from approximately 80 to 100° and the maximum deviations of the *trans*-angles from the ideal symmetry are about 20° for the O–Ga–O angles, while the N–Ga–N angle is 178.4(2)°.

**Figure 8:**
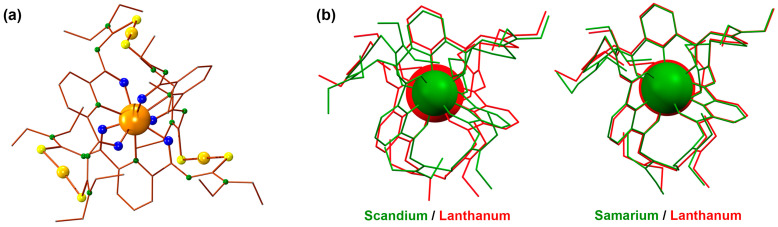
(**a**) Molecular structure of the [Sc⊂{Au_3_(L1^ethyl^)_3_}] (**15**) and (**b**) overlays of the molecular structures of the [Sm⊂{Au_3_(L1^ethyl^)_3_}]/[La⊂{Au_3_(L1^ethyl^)_3_}] and [Sc⊂{Au_3_(L1^ethyl^)_3_}]/[La⊂{Au_3_(L1^ethyl^)_3_}] complexes illustrating the good fit of the two lanthanide structures and the differences between the structures of the lanthanum/scandium complexes due to their different ionic radii. The sizes of the central ions are adopted from their ionic radii given in [55], the Au, S, P and N atoms are shown with arbitrary sizes.

The formation of complexes with coordination numbers < 9 in the present series is restricted to gallium. This is remarkable since, at least for In^3+^ and Sc^3+^, coordination compounds with C.N. 9 are extremely rare [103,104,105,106,107,108,109,110] and, particularly, the few scandium compounds contain a considerable number of aqua ligands or small chelators such as nitrate or boronhydride in their coordination spheres [106,107,108,109,110]. The molecular structures of the indium and scandium compounds are shown in Figure 7 and Figure 8.

Despite the retention of the coordination number, some structural changes are observed when switching from the lanthanide series to the elements of group 3 and group 13. While the M^3+^-N distances are generally larger than the M^3+^-O bonds for the lanthanides (see the graph in Figure 4 and the values in Table 2), this situation gradually changes for the main group and transition metals (Table 3) and in [In⊂{Au_3_(L1^ethyl^)_3_}], the In–pyridine bonds are in the same range or even shorter than the In–O bonds. In the six-coordinate gallium cation, we find a continuation of the observed trend and the Ga-N bonds are shorter than the Ga–O bonds. The coordination environments of the gold atoms in the {Au_3_(L1^ethyl^)_3_}^3−^ and {Au_2_(L1^ethyl^)_2_}^2−^ rings are not significantly influenced by the M^3+^ ions bonded in the central position of the resulting assemblies. Most of the S-Au-S angles show only slight and no systematic deviations from linearity. Moreover, there is no systematic trend in the Au-S or S-C bond lengths in these units depending on the ionic radii of the central ions. Thus, they might be regarded as more or less as rigid units and the observed flexibility of the {Au_3_(L1^ethyl^)_3_}^3−^ and {Au_2_(L1^ethyl^)_2_}^2−^ ring systems is mainly provided by their organic parts, which comes not completely surprising. Mono- and bipodal aroyl-*N*,*N*-(dialkylthioureas) demonstrated their ability to adopt the coordination requirements of various metal ions optimally due to their ability to form highly flexible π-systems in their chelating units [27,28,29,30,31,32,33,34,35,36,37,38,39,40,41,42,43,44,45,46,47,48,49,50,51,52]. The formation of isostructural, nine-coordinate compounds of {Au_3_(L1^ethyl^)_3_}^3−^ coronands with M^3+^ ions, the ionic radii of which range from 1.216 Å (La^3+^) to 0.87 Å (Sc^3+^), necessarily requires such a flexibility in order to direct the donor atoms into optimal positions, from where their lone-pairs point to the central metal ions. This results in gradual differences in the established coordination polyhedra (vide infra) and considerable changes in the arrangement of the organic skeleton of the {Au_3_(L1^ethyl^)_3_}^3−^ rings. The latter fact is illustrated in Figure 8b, where overlays are shown between the lanthanum complex as the one with the largest central ion and [Sm⊂{Au_3_(L1^ethyl^)_3_}] as an example with a ‘medium sized’ lanthanide (ionic radius: 1.132 Å) and with the product containing Sc^3+^ as the smallest M^3+^ ion of this series. Despite the differences in the M-N and M-O bond lengths, the spatial arrangement of the organic skeletons of the {Au_3_(L1^ethyl^)_3_}^3−^ units in the lanthanum and the samarium compounds almost perfectly fits, while clear deviations can be seen between [La⊂{Au_3_(L1^ethyl^)_3_}] and [Sc⊂{Au_3_(L1^ethyl^)_3_}]. A more quantitative discussion of this point, including the consequences concerning the established coordination polyhedra around the M^3+^ ions will be provided in the following subchapter.

### 2.4. More Structural Aspects and Potential Implication for the Reactivity

**Figure 9:**
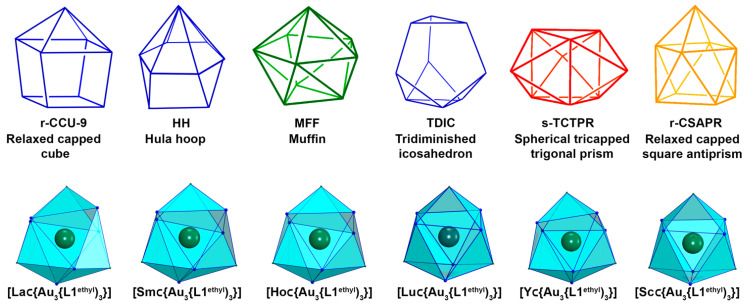
Nine-vertex shapes relevant for the description of coordination compounds according to ref. [113] and experimentally determined coordination polyhedra for selected [M⊂{Au_3_(L1^ethyl^)_3_}] complexes.

There exist no regular Platonic, Archimedean or Catal polyhedra with nine vertices and no prisms or antiprisms can be constructed with an odd number of vertices. *S*. *Alvarez* and co-workers address this point in an excellent, scholarly written paper and derive a number of shapes to be relevant for compounds with C.N. 9 [94]. Among others, they include an octagonal pyramid (OPY-9), a heptagonal bipyramid (HBPY-9), a spherical relaxed capped cube (CCU-9), a spherical capped square antiprism (CSAPR-9), a spherical tricapped trigonal prism (TCTPR-9), a tridiminished icosahedron (JTDIC-9) and a muffin (MFF-9). Some of the classical Johnson polyhedra [111] were adopted to better meet the description of coordination compounds, i. e. providing approximately identical vertex to center distances instead of identical edge lengths, as used for the description of the pure geometrical shapes [94,112]. This results in ‘relaxed’ or ‘spherical’ polyhedra, which are also used in the present considerations and relevant shapes are shown in Figure 9. The ‘Continuous Shape’ approach [94,97,98] allows the evaluation of the bonding situations, even in the case of simple visualization giving no unambiguous results (as in the present case).

Figure 9 illustrates the above mentioned selection of idealized polyhedra applicable for coordination compounds having C.N. 9 [113] together with the coordination polyhedra around some M^3+^ ions derived from the X-ray diffraction studies. The examples were selected in order to cover the entire range of ionic radii, but also to include lanthanides, main group and transition metals. It becomes evident that a plain visual inspection of the polyhedra does not allow to derive differences within the series. This becomes possible by means of the continuous shape algorithm [114,115]. Table 4 contains the continuous shape measures derived for the [M⊂{Au_3_(L1^ethyl^)_3_}] complexes of the present study, considering the six coordination polyhedra regarded as relevant for coordination compounds [113]. It becomes clear that capped cubes, Hula hoop shapes and tridiminished icosahedra do not play any role for any of the complexes. The preferred polyhedron for all [M⊂{Au_3_(L1^ethyl^)_3_}] complexes of the present study is a tricapped trigonal prism (see the corresponding values in Table 4 highlighted in red). However, it also becomes evident that the r-CSAPR-9 (highlighted in orange) and MFF-9 (highlighted in green) shapes play a considerable role in the description of these compounds. It is noteworthy that the fits of the experimental data with the three idealizes shapes are best for the compounds, which have the smallest M^3+^ ions. The best fits are found for the indium compound with continuous shape values of 0.404 for s-TCTPR-9 and 0.910 for r-CSAPR-9. This might have to do with the fact that the M–O and M–N bonds in these compounds are almost equal, while they are clearly different in most of the other compounds (particularly in the representatives of the lanthanide series, see Figure 4 and Table 2 and Table 3).

**Scheme 3:**
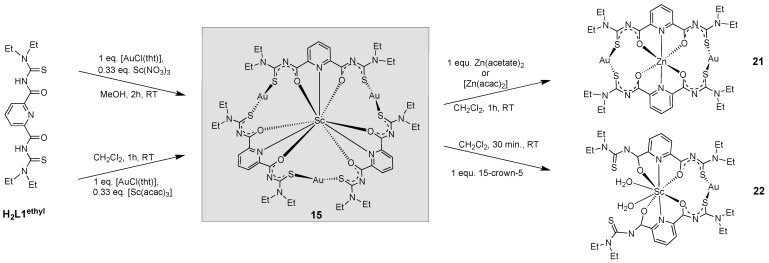
Syntheses and reactions of [Sc⊂{Au_3_(L1^ethyl^)_3_}].

The continuous shape studies on the [M⊂{Au_3_(L1^ethyl^)_3_}] complexes indicate a widespread independence of the established coordination spheres from the size of the M^3+^ ions. Thus, geometrical factors should also not be discussed as driving forces for a potential reactivity of the bimetallic assemblies. Nevertheless, some information about potential reaction patterns of such compounds is desired for the evaluation of potential applications. This particularly concerns their reactivity with other metal ions. Thus, we performed first of such studies with [Sc⊂{Au_3_(L1^ethyl^)_3_}]. As the complex with the smallest M^3+^ ion and a low tendency for stabilization in a nine-coordinate environment, which is documented by the small number of such compounds [106,107,108,109,110], we regarded it as a good candidate for potential transmetalations. Another advantage of scandium is that ^45^Sc NMR is feasible. ^45^Sc has 100% natural abundance, an NMR frequency close to that of ^13^C and a high receptivity (approximately 0.3 relative to ^1^H) [116]. As a quadrupole nucleus with I = 7/2 (Q = −22 fm^2^) [117], ^45^Sc frequently produces NMR spectra with large line widths, particularly for compounds having a low local symmetry around the nucleus. This point might also be a considerable drawback with the nine-coordinate compounds of the present study. Nevertheless, a broad signal at 8.1 ppm (line width 1670 Hz) could be resolved for a CH_2_Cl_2_ solution of [Sc⊂{Au_3_(L1^ethyl^)_3_}]. Thus, we could use this method to monitor some preliminary reactions of the scandium compound as depicted in Scheme 3.

In a first reaction, we exposed [Sc⊂{Au_3_(L1^ethyl^)_3_}] to a solution of zinc acetate in methanol, which resulted in the rapid formation of colorless crystals of [Zn⊂{Au_2_(L1^ethyl^)_2_}] (**21**). This means that in addition to the release of the Sc^3+^ ions from the potentially unfavorable coordination situation with C.N. 9, gold-sulfur bonds of the {Au_3_(L1^ethyl^)_3_}^3−^ rings were cleaved. This comes not completely unexpected in the light of the fact that Zn^2+^ ions preferably form complexes with the coordination numbers four or six. However, the mechanism of the ring cleavage of the {Au_3_(L1^ethyl^)_3_}^3−^ unit and the re-formation of an {Au_2_(L1^ethyl^)_2_}^2−^ ring system under the influence of Zn^2+^ ions is not yet completely clear and requires further studies.

**Figure 10:**
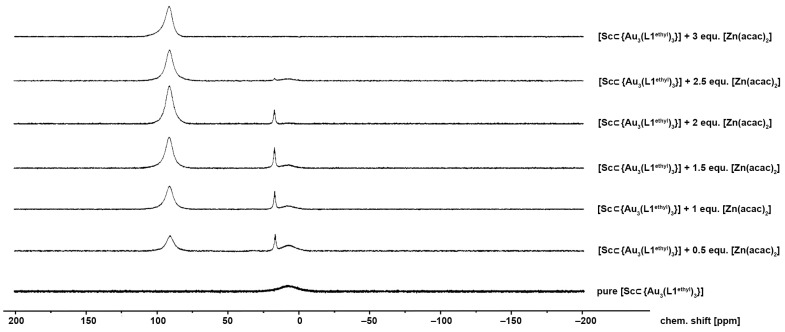
^45^Sc NMR monitoring of the reaction between [Sc⊂{Au_3_(L1^ethyl^)_3_}] and [Zn(acac)_2_] in CH_2_Cl_2_.

Considering that tris(acetylacetonato)scandium(III), [Sc(acac)_3_], can be used as a precursor for the synthesis of [Sc⊂{Au_3_(L1^ethyl^)_3_}] (**15**) (Scheme 3), it is interesting to note that Sc^3+^ can also be released from compound **15** by interacting with [Zn(acac)_2_] and [Sc(acac)_3_] is re-formed. The latter reaction was chosen for a corresponding NMR experiment. Figure 10 depicts a reaction sequence with the subsequent addition of [Zn(acac)_2_] aliquots. The broad signal of the starting material at 6.8 ppm gradually disappears and two new signals appear: a transient, small signal at 17.0 ppm and that of the final scandium-containing product [Sc(acac)_3_] at 90.1 ppm. Unfortunately, we could not isolate the intermediate with the ^45^Sc NMR signal at 17 ppm. Indicated by its small line width, it most probably contains the Sc^3+^ ion in a sterically less restricted form than in the starting compound **15** or [Sc(acac)_3_], where the metal ion establishes a distorted octahedron due to the limitations given by the three chelating acetylacetonato ligands [118].

The existence of such ‘open chain’ compounds was proven by the (unintended) isolation of [Sc(H_2_O)_2_{Au(L1^ethyl^)_2_}] (**22**) from a reaction of compound **15** with the crown ether 15-crown-5 (Scheme 3). We undertook this experiment to see if the Sc^3+^ ions can be removed from [Sc⊂{Au_3_(L1^ethyl^)_3_}] without the destruction of the {Au_3_(L1^ethyl^)_3_}^3+^ ring system. The preliminary results of such reactions suggest that this was obviously not the case. Oxacrown ethers of various ring sizes form stable complexes with Sc^3+^ ions [119,120,121], and indeed 15-crown-5 also reacted with the scandium complex **15**, even in the presence of Na_2_CO_3_. The ‘crown ether-less’ compound **22** is hitherto the only product, which could be isolated in crystalline form from such reactions. However, the (partially intermediate) formation of at least three scandium-containing products is suggested by an NMR monitoring of such reactions. More experimental work is required to understand the mechanism of the transmetalation and the stability of the formed products. The same holds true for reactions with other M^2+^ transition metal ions and other [M^3+^⊂{Au_3_(L1^R^)_3_}], [M^2+^⊂{Au_2_(L1^R^)_2_}] and [M^2+^_2_⊂{Au_4_(L1^R^)_4_}] coronates as precursors. Such work is currently being carried out and the results will be communicated in due course. The two reactions introduced in Scheme 3 shall just demonstrate that such compounds possess a certain reactivity.

**Figure 11:**
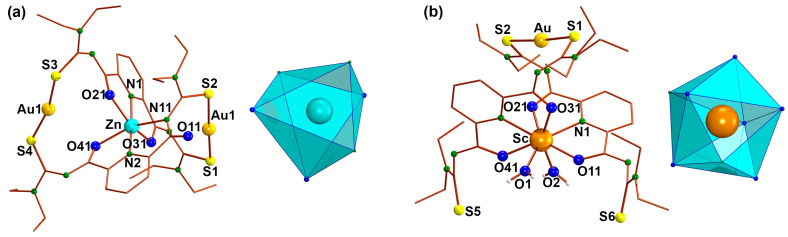
Molecular structures of (**a**) [Zn⊂{Au_2_(L1^ethyl^)_2_}] (**21**) and (**b**) [Sc(H_2_O)_2_{Au(L1^ethyl^)_2_}] (**22**) together with the coordination polyhedra of the Zn^2+^ and Sc^3+^ ions.

Since the complexes [Zn⊂{Au_2_(L1^ethyl^)_2_}] (**21**) and [Sc(H_2_O)_2_{Au(L1^ethyl^)_2_}] (**22**) could be isolated in crystalline form, their structures could be elucidated by X-ray diffraction. Figure 11 shows their molecular structures. Selected bond lengths and angles are shown in Table 5. The Zn^2+^ ion in **21** is coordinated in a distorted octahedral environment. The main distortions were due to the strains induced by the organic backbone of the {Au_2_(L1^ethyl^)_2_}^2−^ ring. Thus, similar to the situation in the [Ga⊂{Au_2_(L1^ethyl^)_2_}]^+^ cations of compounds **18a** and **18b**, there was a deviation up to 15° for *cis* angles and up to 18° for trans angles. The irregularity of the resulting octahedron can be seen in Figure 11a. An unexpected feature in the molecular structure of **21** was the composition of the coordination sphere of Zn^2+^. Unlike the situations in the [M⊂{Au_3_(L1^ethyl^)_3_}] complexes and [Ga⊂{Au_2_(L1^ethyl^)_2_}]^+^, the 2,6-dipicolinoylbis(*N*,*N*-diethylthiourea) units did not exclusively coordinate via a *O*,*N*,*O* donor atom set. One of the central unit used the imido nitrogen atom, establishing a *O*,*N*,*N* donor set for coordination. This might be interpreted as another sign of the structural flexibility of the {Au_2_(L1^ethyl^)_2_}^2−^ coronand and encourages conducting further studies on such ring systems.

The same holds true for the formation of ‘open-chain’ donor systems formed from the gold-containing coronands. The isolation of significant amounts of [Sc(H_2_O)_2_{Au(L1^ethyl^)_2_}] (**22**) was unexpected and might be related to the low solubility of the compound in the reaction mixture. This assumption is supported by the fact that re-dissolution of crystalline **22** in CH_2_Cl_2_ results in solutions containing at least three scandium-containing species according to the corresponding ^45^Sc NMR spectrum.

The scandium atom in [Sc(H_2_O)_2_{Au(L1^ethyl^)_2_}] (**22**) is 8-coordinate with two aqua ligands completing the coordination sphere formed by the *O*,*N*,*O* donor sets of two gold-connected {L1^ethyl^}^2−^ ligands. Similar to the nine-coordinate [M⊂{Au_3_(L1^ethyl^)_3_}] complexes, the coordination polyhedron in compound **22** cannot be assigned to one of the idealized shapes of C.N. 8. The determination of the continuous shape measure for [Sc(H_2_O)_2_{Au(L1^ethyl^)_2_}] describes the coordination sphere of scandium as a triangular dodecahedron (TDD-8, 0.651) with contributions of a triaugmented trigonal prism (BTPR-8, 2.613) and a snub bisphenoid (JSD-8, 2.314) (see Figure 11b).

## 3. Materials and Methods

Unless otherwise stated, reagent-grade starting materials were purchased from commercial sources and either used as received or purified by standard procedures. When purchased as hydrates, the M^3+^ salts were used without additional drying. [AuCl(tht)]) [122], [AuCl(PPh_3_)] [123], H_2_L1^ethyl^ [124] and N,N-diethylthiourea [125] were synthesized according to literature procedures.

### 3.1. Syntheses

*General procedure for the synthesis of [M⊂{Au_3_(L1^ethyl^)_3_}] complexes.* H_2_L1^ethyl^ (59.3 mg, 0.15 mmol) was added to a suspension of [AuCl(tht)] (48.0 mg, 0.15 mmol) and the respective M^3+^ salts (MCl_3_ for M = Gd an Dy; MCl_3_ · 6 H_2_O for M = Nd, Sm, Eu, Er, Yb; M(NO_3_)_3_ for M = In, La, Ho, Tm; M(NO_3_)_3_ · 5 H_2_O for M = Tb, Pr; Ce(NO_3_)_3_ · 6 H_2_O, Sc(NO_3_)_3_ · 4 H_2_O, Lu(NO_3_)_3_ · H_2_O and Y(CF_3_SO_3_)_3_ (0.05 mmol) in 3 mL of MeOH. Most of the mixtures formed a clear solution after 30 minutes, with the exception of the reactions with Er^3+^, Tm^3+^, Yb^3+^, and Lu^3+^, which formed suspensions. The addition of 6 drops of triethylamine resulted in an immediate precipitation of the products as colorless solids. The cerium complex was formed as an orange-red solid. The mixtures were stirred for 90 min. at room temperature. The solids were filtered off, washed with a small amount of MeOH and dried in the air. The complexes were dissolved in 1 mL of CH_2_Cl_2_ or CHCl_3_ and overlayered with 1 mL of MeOH, which was added carefully on the wall of the vial. Single crystals suitable for X-ray diffraction were obtained overnight by slow diffusion of MeOH into the CH_2_Cl_2_ or CHCl_3_ solutions, which were stored in the refrigerator. Elemental analyses of the products were performed on finely powdered samples, which were carefully dried in vacuum.

[*La⊂*{*Au_3_*(*L1^ethyl^*)*_3_*}] (***1***). Colorless crystals. Yield: 90 mg (94%). Elemental analysis: Calcd. for C_51_H_69_N_15_O_6_S_6_Au_3_La: C, 32.1; H, 3.6; N, 11.0; S, 10.1%. Found: C, 32.0; H, 3.7; N, 10.7; S, 10.3%. IR (KBr, cm^−1^): 3066 (vw), 2970 (w), 2933 (w), 2870 (w), 1583 (m), 1558 (vs), 1512 (vs), 1436 (m), 1357 (s), 1244 (s), 1124 (s), 1072 (m), 912 (m), 750 (m), 669 (m), 632 (m), 482 (w), 430 (w), 410 (w). ^1^H NMR (400 MHz, CDCl_3_, ppm): 8.02 (d, J = 8.0 Hz, 2H, py); 7.83 (t, J = 8.0 Hz, 1H, py); 3.68 (m, 2H, CH_2_); 3.50–3.40 (m, 6H, CH_2_); 1.07 (s, br, 12H, CH_3_). ^13^C{^1^H} NMR (CDCl_3_, ppm): 185.7 (C=O); 162.7 (C=S); 153.3, 138.2, 125.2 (py); 46.9, 46.3 (CH_2_); 12.8, 12.5 (CH_3_). ESI^+^ MS (*m*/*z*): 1910.2003, 100% [M+H]^+^ (calcd. 1910.2012). UV-vis: (CH_2_Cl_2_), 227 nm (ε = 156 × 10^3^ M^−1^ cm^−1^), 264 nm (ε = 104 × 10^3^ M^−1^ cm^−1^), 309 nm (ε = 83 × 10^3^ M^−1^ cm^−1^).

[*Ce⊂*{*Au_3_*(*L1^ethyl^*)*_3_*}] (***2***). Orange-red crystals. Yield: 80 mg (84%). Elemental analysis: Calcd. for C_51_H_69_N_15_O_6_S_6_Au_3_Ce: C, 32.0; H, 3.6; N, 11.0; S, 10.1%. Found: C, 32.1; H, 3.7; N, 11.0; S, 10.2%. IR (KBr, cm^−1^): 3066 (vw), 2972 (w), 2933 (w), 2870 (vw), 1583 (m), 1556 (vs), 1512 (vs), 1438 (m), 1398 (m), 1357 (s), 1315 (m), 1244 (s), 1201 (m), 1124 (s), 1070 (m), 1016 (m), 912 (s), 860 (w), 752 (s), 667 (s), 632 (m), 482 (w), 432 (w), 410 (w). ESI^+^ MS (*m*/*z*): 1911.2008, 100% [M+H]^+^ (calcd. 1911.2003); 1933.5769 61% [M+Na]^+^ (calcd. 1933.1823). UV-vis: (CH_2_Cl_2_), 228 nm (ε = 93 × 10^3^ M^−1^ cm^−1^), 265 nm (ε = 67 × 10^3^ M^−1^ cm^−1^), 308 nm (ε = 52 × 10^3^ M^−1^ cm^−1^).

[*Pr⊂*{*Au_3_*(*L1^ethyl^*)*_3_*}] (***3***). Colorless crystals. Yield: 80 mg (84%). Elemental analysis: Calcd. for C_51_H_69_N_15_O_6_S_6_Au_3_Pr: C, 32.0; H, 3.6; N, 11.0%. Found: C, 32.9; H, 3.5; N, 10.8%. IR (KBr, cm^−1^): 3069 (vw), 2972 (w), 2965 (w), 2932 (w), 2871 (vw), 1584 (m), 1558 (vs), 1504 (vs), 1449 (m), 1425 (m), 1409 (m), 1376 (m), 1357 (m), 1315 (m), 1244 (s), 1201 (m), 1146 (m), 1124 (s), 1093 (m), 1071 (m), 1016 (w), 949 (w), 911 (s), 855 (w), 840 (m), 746 (s), 665 (s), 633 (m). ESI^+^ MS (*m*/*z*): 1912.216, 100% [M+H]^+^ (calcd. 1912.203); 1950.116, 5% [M+K]^+^ (calcd. 1950.159).

[*Nd⊂*{*Au_3_*(*L1^ethyl^*)*_3_*}] (***4***). Colorless crystals. Yield: 81 mg (85%). Elemental analysis: Calcd. for C_51_H_69_N_15_O_6_S_6_Au_3_Nd: C, 32.0; H, 3.6; N, 11.0; S, 10.0%. Found: C, 31.8; H, 3.6; N, 10.4; S, 9.7%. IR (KBr, cm^−1^): 3066 (vw), 2972 (w), 2931 (w), 2870 (w), 1585 (m), 1558 (vs), 1512 (vs), 1438 (m), 1400 (m), 1357 (s), 1315 (m), 1246 (s), 1201 (m), 1124 (s), 1070 (m), 1016 (m), 912 (s), 860 (w), 752 (s), 665 (s), 632 (m), 482 (w), 432 (w), 410 (w). ESI^+^ MS (*m*/*z*): 1913.2135, 55% [M+H]^+^ (calcd. 1913.2026); 1935.1957, 52% [M+Na]^+^ (calcd. 1935.1851). UV-vis (CH_2_Cl_2_): 227 nm (ε = 72 × 10^3^ M^−1^ cm^−1^), 264 nm (ε = 49 × 10^3^ M^−1^ cm^−1^), 309 nm (ε = 58 × 10^3^ M^−1^ cm^−1^).

[*Sm⊂*{*Au_3_*(*L1^ethyl^*)*_3_*}] (***5***). Colorless crystals. Yield: 89 mg (92%). Elemental analysis: Calcd. for C_51_H_69_N_15_O_6_S_6_Au_3_Sm: C, 31.9; H, 3.6; N, 10.9; S, 10.0%. Found: C, 31.8; H, 3.6; N, 10.5; S, 10.0%. IR (KBr, cm^−1^): 3066 (vw), 2972 (w), 2931 (w), 2870 (w), 1585 (m), 1560 (vs), 1512 (vs), 1438 (m), 1400 (m), 1357 (s), 1315 (m), 1246 (s), 1203 (m), 1124 (s), 1070 (m), 1018 (w), 912 (s), 860 (w), 752 (s), 663 (m), 632 (m), 484 (w), 432 (w), 412 (w). ESI^+^ MS (*m*/*z*): 1923.2432, 44% [M+H]^+^ (calcd. 1923.2146). UV-vis (CH_2_Cl_2_): 228 nm (ε = 55 × 10^3^ M^−1^ cm^−1^), 264 nm (ε = 37 × 10^3^ M^−1^ cm^−1^), 310 nm (ε = 28 x10^3^ M^−1^ cm^−1^).

[*Eu⊂*{*Au_3_*(*L1^ethyl^*)*_3_*}] (***6***). Colorless crystals. Yield: 90.2 mg (94%). Elemental analysis: Calcd. for C_51_H_69_N_15_O_6_S_6_Au_3_Eu: C, 31.9; H, 3.6; N, 10.9; S, 10.0%. Found: C, 31.9; H, 3.7; N, 10.6; S, 10.2%. IR (KBr, cm^−1^): 3066 (vw), 2970 (w), 2931 (w), 2870 (w), 1587 (m), 1560 (vs), 1508 (vs), 1456 (m), 1357 (m), 1246 (s), 1124 (s), 1070 (m), 912 (m), 750 (m), 663 (m), 634 (m), 484 (w), 432 (w), 412 (w). ESI^+^ MS (*m*/*z*): 1924.2145, 100% [M+H]^+^ (calcd. 1924.2161). UV-vis: (CH_2_Cl_2_), 227 nm (ε = 88 × 10^3^ M^−1^ cm^−1^), 264 nm (ε = 59 × 10^3^ M^−1^ cm^−1^), 309 nm (ε = 44 × 10^3^ M^−1^ cm^−1^).

[*Gd⊂*{*Au_3_*(*L1^ethyl^*)*_3_*}] (***7***). Colorless crystals. Yield: 86 mg (89%). Elemental analysis: Calcd. for C_51_H_69_N_15_O_6_S_6_Au_3_Gd: C, 31.8; H, 3.6; N, 10.9; S, 10.0%. Found: C, 31.4; H, 3.8; N, 10.4; S, 10.0%. IR (KBr, cm^−1^): 3066 (vw), 2970 (w), 2931 (w), 2870 (w), 1587 (m), 1560 (vs), 1512 (vs), 1450 (m), 1400 (m), 1357 (s), 1317 (m), 1246 (s), 1203 (m), 1124 (s), 1070 (m), 1018 (w), 912 (s), 858 (w), 839 (w), 750 (s), 663 (m), 632 (m), 484 (w), 432 (w), 410 (w). ESI^+^ MS (*m*/*z*): 1929.2168, 44% [M+H]^+^ (calcd. 1929.2190). UV-vis (CH_2_Cl_2_): 227 nm (ε = 98 × 10^3^ M^−1^ cm^−1^), 263 nm (ε = 68 × 10^3^ M^−1^ cm^−1^), 309 nm (ε = 50 × 10^3^ M^−1^ cm^−1^).

[*Tb⊂*{*Au_3_*(*L1^ethyl^*)*_3_*}] (***8***). Colorless crystals. Yield: 78 mg (81%). Elemental analysis: Calcd. for C_51_H_69_N_15_O_6_S_6_Au_3_Tb: C, 31.7; H, 3.6; N, 10.9; S, 10.0%. Found: C, 31.9; H, 3.7; N, 10.8; S, 10.1%. IR (KBr, cm^−1^): 3066 (vw), 2970 (w), 2931 (w), 2870 (w), 1587 (m), 1560 (vs), 1508 (vs), 1456 (m), 1357 (m), 1246 (s), 1124 (s), 1070 (m), 912 (m), 750 (m), 663 (m), 634 (m), 484 (w), 432 (w), 412 (w). ESI^+^ MS (*m*/*z*): 1952.2019, 100% [M+Na]^+^ (calcd. 1952.2022). UV-vis (CH_2_Cl_2_): 265 nm (ε = 79 × 10^3^ M^−1^ cm^−1^), 310 nm (ε = 45 × 10^3^ M^−1^ cm^−1^).

[*Dy⊂*{*Au_3_*(*L1^ethyl^*)*_3_*}] (***9***). Colorless crystals. Yield: 93 mg (96%). Elemental analysis: Calcd. for C_51_H_69_N_15_O_6_S_6_Au_3_Dy: C, 31.7; H, 3.6; N, 10.9; S, 10.0%. Found: C, 31.6; H, 3.6; N, 10.3; S, 10.0%. IR (KBr, cm^−1^): 3066 (vw), 2970 (w), 2931 (w), 2870 (w), 1587 (m), 1560 (vs), 1512 (vs), 1458 (m), 1404 (m), 1357 (s), 1317 (m), 1246 (s), 1203 (m), 1124 (s), 1070 (m), 1020 (w), 912 (s), 858 (w), 839 (w), 750 (s), 663 (m), 634 (m), 484 (w), 432 (w), 410 (w). ESI^+^ MS (*m*/*z*): 1935.2172, 100% [M+H]^+^ (calcd. 1935.2241). UV-vis (CH_2_Cl_2_): 227 nm (ε = 334 × 10^3^ M^−1^ cm^−1^), 264 nm (ε = 250 × 10^3^ M^−1^ cm^−1^), 310 nm (ε = 182 × 10^3^ M^−1^ cm^−1^).

[*Ho⊂*{*Au_3_*(*L1^ethyl^*)*_3_*}] (***10***). Colorless crystals. Yield: 83 mg (86%). Elemental analysis: Calcd. for C_51_H_69_N_15_O_6_S_6_Au_3_Ho: C, 31.6; H, 3.6; N, 10.9; S, 9.9%. Found: C, 31.6; H, 3.6; N, 10.3; S, 10.0%. IR (KBr, cm^−1^): 3066 (vw), 2970 (w), 2931 (w), 2870 (w), 1587 (m), 1560 (vs), 1512 (vs), 1458 (m), 1404 (m), 1357 (s), 1317 (m), 1246 (s), 1203 (m), 1124 (s), 1070 (m), 1020 (w), 912 (s), 858 (w), 839 (w), 750 (s), 663 (m), 634 (m), 484 (w), 432 (w), 410 (w). ESI^+^ MS (*m*/*z*): 1936.2224, 45% [M+H]^+^ (calcd. 1936.2252). UV-vis (CH_2_Cl_2_): 265 nm (ε = 53 × 10^3^ M^−1^ cm^−1^), 310 nm (ε = 34 × 10^3^ M^−1^ cm^−1^).

[*Er⊂*{*Au_3_*(*L1^ethyl^*)*_3_*}] (***11***). Colorless crystals. Yield: 86 mg (88%). Elemental analysis: Calcd. for C_51_H_69_N_15_O_6_S_6_Au_3_Er: C, 31.6; H, 3.6; N, 10.8; S, 9.9%. Found: C, 31.6; H, 3.7; N, 10.3; S, 9.9%. IR (KBr, cm^−1^): 3066 (vw), 2970 (w), 2931 (w), 2870 (w), 1587 (m), 1560 (vs), 1512 (vs), 1458 (m), 1404 (m), 1357 (s), 1317 (m), 1246 (s), 1203 (m), 1124 (s), 1070 (m), 1020 (w), 912 (s), 858 (w), 839 (w), 750 (s), 663 (m), 634 (m), 484 (w), 432 (w), 410 (w). ESI^+^ MS (*m*/*z*): 1937.2222, 100% [M+H]^+^ (calcd. 1937.2252). UV-vis (CH_2_Cl_2_): 227 nm (ε = 58 × 10^3^ M^−1^ cm^−1^), 264 nm (ε = 39 × 10^3^ M^−1^ cm^−1^), 309 nm (ε = 27 × 10^3^ M^−1^ cm^−1^).

[*Tm⊂*{*Au_3_*(*L1^ethyl^*)*_3_*}] (***12***). Colorless crystals. Yield: 87 mg (89%).Elemental analysis: Calcd. for C_51_H_69_N_15_O_6_S_6_Au_3_Tm: C, 31.6; H, 3.6; N, 10.8; S, 9.9%. Found: C, 31.2; H, 3.5; N, 10.5; S, 9.9%. IR (KBr, cm^−1^): 3066 (vw), 2970 (w), 2931 (w), 2870 (w), 1587 (m), 1560 (vs), 1512 (vs), 1458 (m), 1404 (m), 1357 (s), 1317 (m), 1246 (s), 1203 (m), 1124 (s), 1070 (m), 1020 (w), 912 (s), 858 (w), 839 (w), 750 (s), 663 (m), 634 (m), 484 (w), 432 (w), 410 (w). ESI^+^ MS (*m*/*z*): 1940.2262, 50% [M+H]^+^ (calcd. 1940.2291). UV-vis (CH_2_Cl_2_): 265 nm (ε = 63 × 10^3^ M^−1^ cm^−1^), 310 nm (ε = 46 × 10^3^ M^−1^ cm^−1^).

[*Yb⊂*{*Au_3_*(*L1^ethyl^*)*_3_*}] (***13***). Colorless crystals. Yield: 86 mg (89%). Elemental analysis Calcd. for C_51_H_69_N_15_O_6_S_6_Au_3_Yb: C, 31.5; H, 3.6; N, 10.8; S, 9.9%. Found: C, 31.5; H, 3.6; N, 10.7; S, 9.9%. IR (KBr, cm^−1^): 3066 (vw), 2970 (w), 2931 (w), 2870 (w), 1591 (m), 1564 (vs), 1514 (vs), 1458 (m), 1359 (m), 1246 (s), 1124 (s), 1070 (m), 914 (m), 856 (m), 750 (m), 661 (m), 634 (m), 486 (w), 430 (w). ESI^+^ MS (*m*/*z*): 1945.2332, 100% [M+H]^+^ (calcd. 1945.2337). UV-vis (CH_2_Cl_2_): 227 nm (ε = 128 x10^3^ M^−1^ cm^−1^), 264 nm (ε = 85 × 10^3^ M^−1^ cm^−1^), 309 nm (ε = 60 × 10^3^M^−1^ cm^−1^).

[*Lu⊂*{*Au_3_*(*L1^ethyl^*)*_3_*}] (***14***). Colorless crystals. Yield: 84 mg (87%). Elemental analysis: Calcd. for C_51_H_69_N_15_O_6_S_6_Au_3_Lu: C, 31.5; H, 3.6; N, 10.8; S, 9.9%. Found: C, 31.5; H, 3.6; N, 10.8; S, 9.8%. IR (KBr, cm^−1^): 2974 (w), 29313 (w), 28720 (w), 1589 (m), 1560 (vs), 1517 (vs), 1458 (m), 1436 (m), 1406 (m), 1382 (m), 1357 (m), 1315 (w), 1294 (w), 1246 (s), 1124 (s), 1068 (m), 914 (m), 756 (w), 659 (w), 632 (w), 484 (w), 437 (w). ESI^+^ MS (*m*/*z*): 1946.2431, 100% [M+H]^+^ (calcd. 1946.2356). UV-vis (CH_2_Cl_2_): 274 nm (ε = 14 × 10^3^ M^−1^ cm^−1^), 310 nm (ε = 18 × 10^3^ M^−1^ cm^−1^).

[*Sc⊂*{*Au_3_*(*L1^ethyl^*)*_3_*}] (***15***). Colorless crystals. Yield: 65.5 mg (72%). Elemental analysis: Calcd. for C_51_H_69_N_15_O_6_S_6_Au_3_Sc: C, 33.7; H, 3.9; N, 11.6; S, 10.6%. Found: C, 33.7; H, 3.9; N, 11.4; S, 10.6%. IR (KBr, cm^−1^): 3072 (vw), 2970 (w), 2931 (w), 2870 (w), 1591 (m), 1564 (vs), 1508 (vs), 1458 (m), 1357 (m), 1246 (s), 1124 (s), 1068 (m), 914 (m), 854 (m), 754 (m), 659 (m), 486 (w), 453 (w), 426 (w). ^1^H NMR (400 MHz, CDCl_3_, ppm): 7.89 (d, J = 8.0 Hz, 2H, py); 7.80 (t, J = 8.0 Hz, 1H, py); 3.72 (m, 2H, CH_2_); 3.45 (m, 6H, CH_2_); 1.08 (t, J = 8.0 Hz, 12H, CH_3_). ^13^C{^1^NMR (CDCl_3_, ppm): 184.4 (C=O); 166.2 (C=S); 150.1, 137.7, 124.2 (py); 46.9, 46.1 (CH_2_); 13.0, 12.6 (CH_3_). ^45^Sc NMR (CH_2_Cl_2_, ppm): 6.8. ESI^+^ MS (m/z): 1816.2544, 100% [M+H]^+^ (calcd. 1816.2508); 1838.2359, 44% [M+Na]^+^ (calcd. 1838.2327). UV-vis (CH_2_Cl_2_): 226 nm (ε = 60 × 10^3^ M^−1^ cm^−1^), 265 nm (ε = 39 × 10^3^ M^−1^ cm^−1^), 311 nm (ε = 26 × 10^3^ M^−1^ cm^−1^).

[*Y⊂*{*Au_3_*(*L1^ethyl^*)*_3_*}] (***16***). Colorless crystals. Yield: 60.8 mg (65%). Elemental analysis: Calcd. for C_51_H_69_N_15_O_6_S_6_Au: C, 32.9; H, 3.7; N, 11.3; S, 10.3%. Found: C, 32.8; H, 3.8; N, 11.3; S, 10.1%. IR (KBr, cm^−1^): 2973 (w), 2931 (w), 2870 (w), 1585 (m), 1557 (s), 1509 (vs), 1435 (br), 1356 (m), 1240 (s), 1121 (m), 1066 (m), 910 (w), 751 (m), 668 (m), 628 (m). ^1^H NMR (400 MHz, CDCl_3_, ppm): 8.08 (m, 2H, py), 7.90 (m, 1H, py), 3.73–3.68 (m, 2H, CH_2_), 3.58 (m, 2H, CH_2_), 3.51–3.45 (m, 2H, CH_2_), 3.37–3.32 (m, 2H, CH_2_), 1.13–1.08 (m, 12H, CH_3_). ESI^+^ MS (m/z): 1860.202, 15.83% [M+H]^+^, (calcd. 1860.197); 1882.185, 18.54% [M+Na]^+^, (calcd. 1882.1827); 1898.158, 7.35% [M+K]^+^, (calcd. 1898.157); 1923.123, 7.35% [M+Na+MeCN]^+^, (calcd. 1923.209).

[*In⊂*{*Au_3_(L1^ethyl^)_3_*}] (***17***). Colorless crystals. Yield: 86.0 mg (91%). Elemental analysis: Calcd. for C_51_H_69_N_15_O_6_S_6_Au_3_In: C, 32.5; H, 3.7; N, 11.1; S, 10.2%. Found: C, 32.6; H, 3.9; N, 11.2; S, 10.4%. IR (KBr, cm^−1^): 2974 (m), 2931 (m), 2870 (w), 1597 (m), 1560 (vs), 1516 (vs), 1458 (m), 1431 (m), 1408 (m), 1377 (w), 1357 (s), 1319 (m), 1246 (s), 1205 (m), 1124 (s), 1074 (m), 914 (m), 862 (w), 750 (m), 663 (m), 653 (m), 634 (m), 486 (w), 449 (w). ^1^H NMR (400 MHz, CDCl_3_, ppm): 8.15 (d, J = 8.0 Hz, 2H, py); 7.89 (t, J = 8.0 Hz, 1H, py); 3.73 (m, 2H, CH_2_); 3.45 (m, 6H, CH_2_); 1.09 (br, 12H, CH_3_). ^13^C{^1^H} NMR (CDCl_3_, ppm): 183.1 (C=O); 162.6 (C=S); 145.7, 138.5, 125.1 (py); 46.7, 46.1 (CH_2_); 12.9, 12.7 (CH_3_). ESI^+^ MS (m/z): 1295.1030, 100% [M-Au-L1^ethyl^+H]^+^ (calcd. 1295.1202), 1908.1793, 5% [M+Na]^+^ (calcd. 1908.1813). UV-vis (CH_2_Cl_2_): 228 nm (ε = 69 *×* 10^3^ M^−1^ cm^−1^), 262 nm (ε = 42 *×* 10^3^ M^−1^ cm^−1^), 308 nm (ε = 30 *×* 10^3^ M^−1^ cm^−1^).

[*Ga⊂*{*Au_2_*(*L1^ethyl^*)*_2_*}](*NO_3_*) (***18a***). H_2_L1^ethyl^ (39.9 mg, 0.1 mmol) was added to a suspension of [AuCl(tht)] (32.5 mg, 0.1 mmol) and Ga(NO_3_)_3_·nH_2_O (12.8 mg, 0.05 mmol) in 3 mL of MeOH. The mixture was stirred at room temperature for 30 minutes and 6 drops of triethylamine were added. The mixture was stirred for additional 1.5 h. During this time, a colorless precipitate was obtained, which was filtered off, washed with a small amount of MeOH and dried in the air. The solid was dissolved in 0.5 mL of CH_2_Cl_2_ and 0.5 mL of CHCl_3_. The solution was overlayered with 1 mL of MeOH, which was added carefully on the wall of the vial. Single crystals suitable for X-ray diffraction were obtained from slow diffusion of MeOH into the CHCl_3_/CH_2_Cl_2_ solution. Colorless crystals. Yield: 44.0 mg (63%). Elemental analysis: Calcd. for C_36_H_50_._5_Au_2_Cl_3_._5_N_11_O_7_._5_S_4_Ga ([Ga{Au_2_(L1^ethyl^)_2_}](NO_3_) · 0.5 CHCl_3_ ·CH_2_Cl_2_ · 0.5 MeOH): C, 30.1; H, 3.5; N, 10.7; S, 8.9%. Found: C, 28.7; H, 3.5; N, 10.6; S, 9.6%. IR (KBr, cm^−1^): 3066 (vw), 2970 (w), 2931 (w), 2870 (w), 1591 (m), 1564 (vs), 1514 (vs), 1458 (m), 1359 (m), 1246 (s), 1124 (s), 1070 (m), 914 (m), 845 (br), 833 (br), 750 (m), 661 (m), 634 (m), 558 (m), 486 (w), 430 (w). ^1^H NMR (400 MHz, CDCl_3_, ppm): 8.1 (d, J = 8.0 Hz, 2H, py); 7.68 (t, J = 8.0 Hz, 1H, py); 3.73 (m, 2H, CH_2_); 3.45 (m, 6H, CH_2_); 1.1 (br, 12H, CH_3_). ^13^C{^1^H} NMR (CDCl_3_, ppm): 182.8 (C=O); 162.2 (C=S); 145.5, 137.5, 125.7 (Py); 46.4, 46.6 (CH_2_); 12.8, 12.9 (CH_3_). ESI^+^ MS (m/z): 1249.1341, 100% [M]^+^ (calcd. 1249.1168). UV-vis (CH_2_Cl_2_): 227 nm (ε = 30 x10^3^ M^−1^ cm^−1^), 260 nm (ε = 35 × 10^3^ M^−1^ cm^−1^), 307 nm (ε = 28 × 10^3^ M^−1^ cm^−1^).

[*Ga⊂*{*Au_2_*(*L1^ethyl^*)*_2_*}](*BF_4_*) (***18b***). H_2_L1^ethyl^ (39.6 mg, 0.10 mmol) was added to a suspension of [AuCl(tht)] (32.1 mg, 0.10 mmol), [Ga(acac)_3_] (18.5 mg, 0.05 mmol) and KBF_4_ (12.6 mg, 0.1 mmol) in MeOH (2 mL). Triethylamine (5 drops) was added and the reaction mixture was stirred at room temperature for 2 hours, which led to the formation of a colorless precipitate. The solid was filtered off, washed with n-hexane and dried under vacuum. Single crystals for an X-ray analysis were obtained by dissolving the product in CH_2_Cl_2_, overlaying the solution with a double volume of diethyl ether and keeping it at −15 °C for 2 days. Colorless crystals. Yield: 50.0 mg (72%). Elemental analysis: Calcd for C_35_H_48_Au_2_BF_4_GaN_10_O_4_S_4_Cl_2_ ([Ga{Au_2_(L1^ethyl^)_2_}](BF_4_) · CH_2_Cl_2_): C, 29.6; H, 3.4; N, 9.9; S, 9.0%. Found: C, 30.0; H, 3.5; N, 10.1; S, 9.2%.IR (ATR, cm^−^1): 3447 (w), 2975 (vw), 2961 (w), 2935 (w), 2874 (w), 1613 (s), 1579 (s), 1518 (s), 1437 (m), 1357 (s), 1242 (s), 1078 (br), 955 (w), 916 (s), 862 (m), 773 (s), 762 (s), 674 (m), 642 (m). ^1^H NMR (400 MHz, DMSO-d_6_, ppm): 8.85 (ddd, J = 9.8, 7.5, 2.3 Hz, 2H), 8.78–8.72 (m, 4H), 4.03–3.90 (m, 8H), 3.67 (dq, J = 14.6, 7.2 Hz, 8H), 3.44 (dq, J = 13.2, 7.2 Hz, 8H), 1.24 (q, J = 7.2 Hz, 12H), 0.96 (t, J = 7.2 Hz, 12H). ^19^F-NMR (DMSO-d_6_, ppm): –48.18. ESI+ MS (m/z): 1249.122, [M]^+^ (calcd. 1249.117).

[{*AuCl*}*_2_*(*H_2_L1^ethyl^*)] (***19***). [AuCl(tht)] (64 mg, 0.2 mmol) was added to a solution of H_2_L1^ethyl^ (40 mg, 0.1 mmol) in 3 mL of hot THF. After a few minutes, the suspension became clear and a colorless solid precipitated. The reaction mixture was stirred for 2 hours at room temperature and the precipitate was filtered off and washed with THF. The solid was dissolved in 2 mL of CH_2_Cl_2_ and overlayered with 2 mL of MeOH. Single crystals suitable for X-ray diffraction were obtained from slow diffusion of the MeOH into the CH_2_Cl_2_ solution. Colorless crystals. Yield: 55 mg (64%). Elemental analysis: Calcd. for C_17_H_25_Au_2_Cl_2_N_5_O_2_S_2_: C, 23.7; H, 2.9; N, 8.1; S, 7.5%. Found: C, 23.7; H, 2.9; N, 8.2; S, 7.8%. IR (KBr, cm^−1^): 3302 (s), 2976 (w), 2930 (w), 1707 (s), 1560 (s), 1446 (s), 1290 (m), 1224 (m), 1076 (w), 999 (w), 842 (w), 750 (m). ^1^H NMR (400 MHz, CDCl_3_, ppm): 10.83 (s, 2H, NH); 8.42 (d, J = 7.8 Hz, 2H, Ph); 8.20 (t, J = 8 Hz, 1H, Ph); 4.02 (q, J = 7 Hz, 4H, CH_2_); 3.75 (q, J = 7.2 Hz, 4H, CH_2_); 1.49 (t, J = 7.2 Hz, 3H); 1.42 (t, J = 7.2 Hz, 3H). ^13^C{^1^H} NMR (CDCl_3_, ppm): 177.4 (C=O); 157.3 (C=S); 147.4, 140.2, 127.5 (py); 51.4, 48.9 (CH_2_); 12.8, 11.6 (CH_3_). ESI^+^ MS (m/z): 592.1175, 100% [M-Au-2Cl]^+^ (calcd. 592.1110).

[{*Au*(*PPh_3_*}*_2_*(*L1^ethyl^*)] (***20***). A solution of H_2_L1^ethyl^ (40 mg, 0.1 mmol) in 3 mL of MeOH was added to a suspension of [AuCl(PPh_3_)] (100 mg, 0.2 mmol) in 5 mL of MeOH. After addition of 2 drops of Et_3_N, the suspension became a clear yellow solution. The mixture was stirred for 2 hours at room temperature and kept in a freezer for crystallization. Colorless crystals were obtained after one week. Colorless crystals. Yield: 60 mg (46%). Elemental analysis: Calcd. for C_53_H_53_Au_2_N_5_O_2_P_2_S_2_: C, 48.5; H, 4.1; N, 5.3; S, 4.9%. Found: C, 48.4; H, 4.2; N, 5.6; S, 4.9%. IR (KBr, cm^−1^): 3069 (w), 3049 (w), 2976 (w), 2932 (w), 2870 (w), 1610 (m), 1558 (m), 1500 (s), 1435 (s), 1355 (s), 1240 (s), 1101 (s), 997 (m), 900 (m), 848 (m), 746 (s), 692 (s), 538 (s), 503 (s), 401 (m). ESI^+^ MS (m/z): 1334.2305, 26% [M+Na]^+^ (Calcd. 1334.2339). The acquisition of NMR spectra was not possible due to the low solubility of the compound in organic solvents.

[*Zn⊂*{*Au_2_*(*L1^ethyl^*)*_2_*}] (***21***). [Zn(acac)_3_] (26 mg, 0.1 mmol) was dissolved in 1 mL MeOH and added dropwise to a solution of [Sc⊂{Au_3_(L1^ethyl^)_3_}] (181 mg, 0.1 mmol) in 1 mL CH_2_Cl_2_. The solution was stirred at room temperature for 1 h and then kept for 2 days at room temperature to obtain single crystals for X-ray analysis by slow evaporation of the solvents. Colorless crystals. Yield: 47 mg (38%). Elemental analysis: Calcd. for C_34_H_46_N_10_O_4_S_4_Au_2_Zn: C, 32.8; H, 3.7; N, 11.3; S, 10.3%. Found: C, 32.8; H, 3.8; N, 11.3; S, 10.3%. IR (KBr, cm^−1^): 3433 (w), 2974 (w), 2931 (w), 2870 (w), 1589 (w), 1560 (vs), 1508 (vs), 1431 (s), 1404 (s), 1355 (s), 1313 (m), 1242 (s), 1122 (m), 1070 (m), 842 (w), 759 (s), 638 (m), 484 (w), 430 (w). ESI+ MS (m/z)**:** 1245.1352, 100% [M+H]^+^ (calcd. 1245.1282).

[*Sc*(*H_2_O*)*_2_*{*Au*(*L1^ethyl^*)*_2_*}] (***22***). 15-Crown-5 (0.03 mmol) and Na(OAc) · 3 H_2_O (2.5 mg, 0.03 mmol) were dissolved in 1 mL of CH_2_Cl_2_. After 15 min, [Sc(acac)_3_] (50 mg, 0.03 mmol) was added and the solution was stirred for an additional hour. The solution was overlayered with MeOH and kept for crystallization in a refrigerator. A crystalline material deposited within one week. Colorless crystals. Yield: mg (47%). IR (KBr, cm^−1^): 2974 (w), 2931 (w), 1591 (w), 1560 (vs), 1521 (s), 1436 (m), 1398 (m), 1377 (w), 1357 (m), 1315 (w), 1296 (w), 1246 (m), 1205 (w), 1124 (m), 1124 (m), 1093 (w), 1068 (w), 952 (w), 914 (w), 869 (w), 756 (w), 659 (w), 632 (w), 488 (w), 447 (w). ^45^Sc NMR (CH_2_Cl_2_, ppm): 12.1.

### 3.2. Spectroscopic Methods

Elemental analyses of carbon, hydrogen, nitrogen and sulfur were determined using a Heraeus vario EL elemental analyzer. IR spectra were measured as KBr pellets on a Shimadzu IR Affinity-1 spectrometer between 400 and 4000 cm^−1^ or a Thermo Scientific Nicolet iS10 ATR spectrometer. The NMR spectra were recorded on a JEOL 400 MHz spectrometer. ESI TOF mass spectra were measured with an Agilent 6210 ESI TOF (Agilent Technologies, Santa Clara, CA, USA). All MS results are given in the form: m/z, assignment. UV-vis spectra were recorded on a Specord 40 (Analytik Jena) spectrophotometer in the wavelength range 200–700 nm. Time-Resolved Laser Fluorescence (TRLFS) was performed at T < 20 K using a pulsed Nd:YAG (Spectra Physics) pumped dye laser system (Radiant Dyes Narrow Scan K). The detection was carried out with a spectrograph (Shamrock 303i) equipped with a polychromator with 300, 600 and 1200 lines/mm gratings and an ICCD camera system (Andor iStar). Common concentrations were applied for the spectroscopic studies. In some cases, they were limited by the low solubility of the compounds.

### 3.3. X-ray Crystallography

The intensities for the X-ray determinations were collected on STOE IPDS-2T or Bruker CCD instruments with Mo/Kα radiation. The various temperatures applied are due to the experimental setup of the different diffractometers. Semi-empirical or numerical absorption corrections were carried out by the SADABS or X-RED32 programs [126,127]. Structure solution and refinement were performed with the SHELX programs [128,129] included in the WinGX [130] program package or OLEX2 [131]. Hydrogen atoms were calculated for the idealized positions and treated with the ‘riding model’ option of SHELXL. Since some of the lanthanide compounds crystallized together with disordered solvent molecules (partially close to special positions), the refinements of such structures were undertaken with the removal of the disordered solvent molecules using the SQUEEZE option installed in the program PLATON or the solvent mask option of OLEX2. Details are given in the Supplementary Materials. The representation of molecular structures was done using the program DIAMOND [132].

## 4. Conclusions

2,6-Dipicolinoylbis(*N*,*N*-diethylthiourea), H_2_L1^ethyl^, reacts with a mixture of [Au(Cl(tht)] and trivalent metal ions under formation of a novel class of self-assembled compounds of the compositions [M⊂{Au_3_(L1^ethyl^)_3_}] or [M⊂{Au_2_(L1^ethyl^)_2_}]^+^. The basic structural motifs are gold-based {Au_3_(L1^ethyl^)_3_}^3−^ or {Au_2_(L1^ethyl^)_2_}^2−^ coronands, which host M^3+^ ions from the lanthanide series or the ‘group 3′ and ‘group 13′ elements. Except for the very small Ga^3+^ ions, nine-coordinate environments are established around the M^3+^ ions. This is particularly surprising for small ions such as In^3+^ or Sc^3+^, as nine-coordinate complexes are extremely rare for them.

Transmetalation reactions starting from the scandium complex gives access to corresponding compounds with bivalent transition metal ions. The mechanism of such reactions is hitherto not clear and require more detailed studies, which should also give information about the stabilities of such assemblies and their synthetic potential.

## Figures and Tables

**Table 1:** Selected bond lengths (Å) in the [(AuCl)_2_(H_2_L^ethyl^)] (**19**) and [{Au(PPh_3_)}_2_(L1^ethyl^)] (**20**) complexes and in the uncoordinated ligand H_2_L1^ethyl^ [42].

	Au1–S1	S1–C12	C12–N12	C12–N11	N11–C11	Au2–S2	S2–C22	C22–N22	C22–N21	N21–C21
**19**	2.266(1)	1.710(4)	1.305(5)	1.408(5)	1.375(5)	2.270(1)	1.717(4)	1.313(5)	1.398(5)	1.382(5)
**20**	2.296(3)	1.73(1) ^1^	1.36(2) ^1^	1.33(2)	1.22(3)					
H_2_L1^ethyl^ [42]		1.62(2)	1.35(2) ^1^	1.45(2)	1.33(2)		1.65(2)	1.32(2)	1.43(2)	1.35(2)

^1^ Mean value from disordered regions of the structure.

**Table 2:** Selected bond lengths (Å) and angles (°) in the [M⊂{Au_3_(L1^ethyl^)_3_}] complexes.

	La	Ce	Pr	Nd	Sm	Eu	Gd	Tb	Dy	Ho	Er	Tm	Yb	Lu
S. G. ^1^	$P\bar{3}$	$P\bar{3}$	$P\bar{3}$	$P\bar{3}$	$P\bar{3}$	$P\bar{3}$	$P\bar{3}$	$P\bar{3}$	$P\bar{3}$	$P\bar{3}$	$P\bar{3}$	C2/c	$P\bar{3}$	$P\bar{1}$
Radii ^2^	1.216	1.196	1.179	1.163	1.132	1.12	1.107	1.095	1.083	1.072	1.062	1.052	1.042	1.032
M-N	2.657(6)	2.623(6)	2.589(9)	2.594(6)	2.554(8)	2.54(1)	2.529(6)	2.514(3)	2.49(1)	2.487(4)	2.480(6)	2.444(6) 2.454(3)	2.45(1)	2.429(6)– 2.447(6) ^3^
M-O	2.510(6) 2.555(5)	2.525(5) 2.482(5)	2.471(9) 2.520(9)	2.502(5) 2.461(5)	2.465(7) 2.428(7)	2.453(8) 2.421(9)	2.445(5) 2.405(5)	2.435(3) 2.392(3)	2.378(8) 2.420(7)	2.417(3) 2.371(4)	2.372(5) 2.428(5)	2.378(3) 2.342(3) 2.403(3)	2.371(9) 2.34(1)	2.298(5)– 2.391(5) ^4^
Au-S	2.267(2) 2.276(3)	2.274(2) 2.284(2)	2.278(4) 2.294(4)	2.290(2) 2.277(2)	2.774(3) 2.285(3)	2.280(3) 2.289(4)	2.276(2) 2.291(2)	2.278(1) 2.289(1)	2.279(3) 2.291(4)	2.281(1) 2.294(2)	2.295(2) 2.313(2)	2.286(2) 2.277(2) 2.284(1)	2.292(4) 2.275(4)	2.269(2)– 2.289(2) ^5^
C-S	1.739(8) 1.74(1)	1.73(1) 1.738(7)	1.69(2) 1.74(1)	1.744(7) 1.742(9)	1.74(1) 1.75(1)	1.73(2) 1.74(1)	1.747(8) 1.74(1)	1.743(5) 1.74(4)	1.74(2) 1.74(1)	1.741(5) 1.748(7)	1.734(8) 1.747(9)	1.730(6) 1.747(5) 1.75(1)	1.76(2) 1.73(2)	1.729(9)– 1.777(9) ^6^
S-Au-S	172.8(1)	173.9(1)	173.8(2)	173.54(9)	173.9(1)	174.2(2)	174.1(1)	173.97(5)	174.4(2)	174.26(7)	174.4(1)	176.80(5) 168.91(7)	174.4(2)	174.6(1)–177.4(1) ^7^

^1^ Space group; ^2^ Ionic radii of the M^3+^ ions in Å for coordination number 9 taken from ref. [55]; ^3^ Individual values: 2.429(6), 2.447(6), 2.434(6) Å; ^4^ Individual values: 2.367(5), 2.365(5), 2.342(5), 2.371(5), 2.391(5), 2.298(5) Å; ^5^ Individual values: 2.286(2), 2.289(2), 2.289(2), 2.280(2), 2.282(2), 2.269(2) Å; ^6^ Individual values: 1.777(9), 1.742(9), 1.741(9), 1.729(9), 1.753(8), 1.739(9) Å; ^7^ Individual values: 177.37(9), 174.57(9), 175.8(1)°.

**Table 3:** Selected bond lengths (Å) and angles (°) in [Sc⊂{Au_3_(L1^ethyl^)_3_}] (**15**), [Y⊂{Au_3_(L1^ethyl^)_3_}] (**16**), [In⊂{Au_3_(L1^ethyl^)_3_}] (**17**), [Ga⊂{Au_2_(L1^ethyl^)_2_}](NO_3_) (**18a**) and [Ga⊂{Au_2_(L1^ethyl^)_2_}](BF_4_) (**18b**).

	Sc (15)	Y (16)	In (17)	Ga (18a)	Ga (18b)
S. G. ^1^	$P\bar{1}$	*P*2_1_/*c*	*C*2/*c*	*P*2_1_/*c*	*P*2_1_/*c*
Radii ^2^	0.87 (for C.N. 8)	1.075 (for C.N. 8)	0.92 (for C.N. 8)	0.62 (for C.N. 6)	0.62 (for C.N. 6)
M-N	2.350(2), 2.350(2), 2.365(2)	2.50(1), 2.50(1), 2.49(1)	2.330(3), 2.334(2)	1.961(4), 1.950(4)	1.90(2), 1.95(2)
M-O	2.248(2), 2.291(2), 2.221(2), 2.291(2), 2.294(2), 2.316(2)	2.441(9), 2.337(9), 2.362(8), 2.352(9), 2.281(9), 2.463(9)	2.354(2), 2.322(2), 2.391(3)	1.977(4), 1.988(3), 2.010(4), 1.991(4)	1.98(1), 1.96(1), 1.97(1), 1.97(1)
Au-S	2.2891(6), 2.2838(6), 2.2811(6), 2.2923(6), 2.2915(6), 2.2771(6)	2.2888(5), 2.302(4), 2.290(5), 2.289(5), 2.294(4), 2.289(4)	2.287(1), 2.290(1), 2.402(7) ^3^	2.277(2), 2.279(2), 2.2757(1), 2.2670(1)	2.270(7), 2.268(7), 2.278(7), 2.303(7)
C-S	1.760(2), 1.754(2), 1.750(2), 1.741(2), 1.749(2), 1.736(2)	1.74(2), 1.73(1), 1.73(2), 1.72(2), 1.73(1), 1.73(2)	1.761(4), 1.740(4), 1.75(1) ^3^	1.738(6), 1.736(5), 1.738(6), 1.723(5)	1.71(3), 1.78(2), 1.71(2), 1.77(2)
S-Au-S	176.61(2), 174.15(2), 175.90(2)	175.6(1), 177.4(2), 1.76.8(1)	168.44(6), 174.2(2) ^3^	166.70(5), 167.29(5)	168.2(2), 1.669(2)

^1^ Space group; ^2^ Ionic radii of the M^3+^ ions in Å taken from [55], where no radii for Y^3+^, In^3+^ and Ga^3+^ with C.N. are available, ^3^ Main component of disordered unit.

**Table 4:** Continuous shape measures derived for the nine-coordinate [M⊂{Au_3_(L1^ethyl^)_3_}] species. Preferred polyhedra are highlighted in color (the used colors refer to those used in Figure 9).

	Capped Cube r-CCU-9 ^1^	Hula Hoop HH-9	Muffin MFF-9	Tridiminished Icosahedron TDIC-9	Tricapped Trigonal Prism s-TCTPR-9 ^1^	Capped Square Antiprism r-CSAPR-9 ^1^
La (**1**)	8.675	11.876	**2.743**	10.652	**2.230**	**2.416**
Ce(**2**)	8.709	11.890	**2.627**	10.551	**2.086**	**2.309**
Pr (**3**)	8.647	11.892	**2.562**	10.546	**1.949**	**2.206**
Nd (**4**)	8.813	11.873	**2.415**	10.888	**1.769**	**2.027**
Sm (**5**)	8.900	11.915	**2.275**	11.101	**1.567**	**1.857**
Eu (**6**)	8.932	11.957	**2.210**	11.033	**1.455**	**1.779**
Gd (**7**)	8.967	11.962	**2.181**	11.099	**1.390**	**1.727**
Tb (**8**)	9.019	11.977	**2.131**	11.220	**1.309**	**1.658**
Dy (**9**)	9.053	12.051	**2.061**	11.263	**1.186**	**1.570**
Y (**10**)	9.134	11.881	**1.835**	12.749	**1.203**	**1.157**
Ho (**11**)	9.080	12.081	**2.041**	11.240	**1.125**	**1.531**
Er (**12**)	9.127	12.140	**1.986**	11.133	**0.993**	**1.450**
Tm (**13**)	9.016	11.993	**1.847**	12.723	**0.978**	**1.311**
Yb (**14**)	9.194	12.146	**1.963**	11.452	**1.029**	**1.448**
Lu (**15**)	9.265	11.952	**1.876**	12.370	**0.926**	**1.228**
In (**16**)	9.790	12.708	**1.503**	12.972	**0.406**	**0.910**
Sc (**17**)	9.707	12.229	**1.633**	12.711	**0.614**	**0.986**

^1^ The prefixes ‘r’ and ‘s’ refer to the spherically relaxed or the spherical versions of the ideal Johnson polyhedra [94,113].

**Table 5:** Selected bond lengths and angles in [Zn⊂{Au_2_(L1^ethyl^)_2_}] (**21**) and [Sc(H_2_O)_2_{Au(L1^ethyl^)_2_}] (**22**).

**[Zn** **⊂{Au_2_(L1^ethyl^)_2_}] (21)**												
Zn–N1		2.035(3)	Zn–N2		2.050(3)		Zn–N11	2.221(3)		Zn–O21		2.154(3)
Zn–O31		2.120(2)	Zn-O41		2.181(2)		Au1-S1	2.283(1)		Au1–S3		2.291(1)
Au2–S2		2.289(1)	Au2-S4		2.289(1)		N1–Zn–N2	174.8(1)		N1–Zn–O21		77.1(1)
N1–Zn–O31		108.6(1)	N1–Zn–O21		99.4(1)		N1–Zn–N11	75.0(1)		N2–Zn–O21		101.8(2)
N2–Zn–O31		76.5(1)	N2–Zn–O41		75.5(1)		N2–Zn–N11	105.9(1)		O21–Zn–N11		152.0(1)
O21–Zn–O41		90.5(1)	O31–Zn–N11		97.3(1)		O31–Zn–O21	93.8(1)		O31–Zn–O41		151.87(9)
O41–Zn–N11		92.7(1)	S1–Au1–S3		173.24(4)		S2–Au2–S4	166.98(4)				
**[Sc(H_2_O)_2_{Au(L1^ethyl^)_2_}] (22)**												
Sc–O1	2.224(6)/2.147(7)			Sc–O2		2.171(6)/2.177(6)			Sc-N1		2.288(6)/2.322(8)	
Sc–O11	2.198(6)/2.269(6)			Sc–O21		2.190(6)/2.153(6)			Sc–N2		2.304(6)/2.339(7)	
Sc–O31	2.210(6)/2.197(7)			Sc–O41		2.209(6)/2.252(7)			Au1–S1		2.275(4)/2.284(3)	
Au2–S2	2.280(3)/2.283(3)											

## Data Availability

Not applicable.

## Supplementary Materials

The following supporting information can be downloaded at: https://www.mdpi.com/article/10.3390/molecules28145421/s1, Table S1. Crystallographic data and data collection parameters; Figure S1. Ellipsoid representation of [La⊂{Au_3_(L1^ethyl^)_3_}] (**1**) including the positional disorder in the peripheral ethyl groups. The thermal ellipsoids are set at a 50% probability level. Hydrogen atoms are omitted for clarity. A solvent mask was calculated and 53 electrons were found in a volume of 218 A^3^ in 3 voids per unit cell. This is consistent with the presence of 2.5 H_2_O per formula unit which account for 50 electrons per unit cell; Figure S2. Ellipsoid representation of [Ce⊂{Au_3_(L1^ethyl^)_3_}] (**2**) including the positional disorder in the peripheral ethyl groups. The thermal ellipsoids are set at a 50% probability level. Hydrogen atoms are omitted for clarity; Figure S3: Ellipsoid representation of [Pr⊂{Au_3_(L1^ethyl^)_3_}] (**3**) including the positional disorder in the peripheral ethyl groups. The thermal ellipsoids are set at a 50% probability level. Hydrogen atoms are omitted for clarity; Figure S4. Ellipsoid representation of [Nd⊂{Au_3_(L1^ethyl^)_3_}] (**4**) including the positional disorder in the peripheral ethyl groups. The thermal ellipsoids are set at a 50% probability level. Hydrogen atoms are omitted for clarity. A solvent mask was calculated and 54 electrons were found in a volume of 247 A^3^ in 4 voids per unit cell. This is consistent with the presence of 2.5 H_2_O per formula unit which account for 50 electrons per unit cell; Figure S5. Ellipsoid representation of [Sm⊂{Au_3_(L1^ethyl^)_3_}] (**5**). The thermal ellipsoids are set at a 50% probability level. Hydrogen atoms are omitted for clarity. A solvent mask was calculated and 55 electrons were found in a volume of 240 A^3^ in 4 voids per unit cell. This is consistent with the presence of 2.5 H_2_O per formula unit which account for 50 electrons per unit cell; Figure S6. Ellipsoid representation of [Eu⊂{Au_3_(L1^ethyl^)_3_}] (**6**). The thermal ellipsoids are set at a 50% probability level. Hydrogen atoms are omitted for clarity. A solvent mask was calculated and 46 electrons were found in a volume of 227 A^3^ in 3 voids per unit cell. This is consistent with the presence of 2.5 H_2_O per formula unit which account for 50 electrons per unit cell; Figure S7. Ellipsoid representation of [Gd⊂{Au_3_(L1^ethyl^)_3_}] (**7**). The thermal ellipsoids are set at a 50% probability level. Hydrogen atoms are omitted for clarity. A solvent mask was calculated and 65 electrons were found in a volume of 179 A^3^ in 2 voids per unit cell. This is consistent with the presence of 3.5 H_2_O per formula unit which account for 70 electrons per unit cell; Figure S8. Ellipsoid representation of [Tb⊂{Au_3_(L1^ethyl^)_3_}] (**8**) including the positional disorder in the peripheral ethyl groups. The thermal ellipsoids are set at a 50% probability level. Hydrogen atoms are omitted for clarity. A solvent mask was calculated and 48 electrons were found in a volume of 252 A^3^ in 3 voids per unit cell. This is consistent with the presence of 2.5 H_2_O per formula unit which account for 50 electrons per unit cell; Figure S9. Ellipsoid representation of [Dy⊂{Au_3_(L1^ethyl^)_3_}] (**9**). The thermal ellipsoids are set at a 50% probability level. Hydrogen atoms are omitted for clarity. A solvent mask was calculated and 43 electrons were found in a volume of 138 A^3^ in 1 void per unit cell. This is consistent with the presence of 2 H_2_O per formula unit which account for 40 electrons per unit cell; Figure S10. Ellipsoid representation of [Ho⊂{Au_3_(L1^ethyl^)_3_}] (**10**) including the positional disorder in the peripheral ethyl groups. The thermal ellipsoids are set at a 50% probability level. Hydrogen atoms are omitted for clarity. A solvent mask was calculated and 43 electrons were found in a volume of 282 A^3^ in 3 voids per unit cell. This is consistent with the presence of 2 H_2_O per formula unit which account for 40 electrons per unit cell; Figure S11. Ellipsoid representation of [Er⊂{Au_3_(L1^ethyl^)_3_}] (**11**) including the positional disorder in the peripheral N(ethyl)_2_ groups. The thermal ellipsoids are set at a 50% probability level. Hydrogen atoms are omitted for clarity; Figure S12. Ellipsoid representation of [Tm⊂{Au_3_(L1^ethyl^)_3_}] (**12**) including the positional disorder in the peripheral N(ethyl)_2_ groups. The thermal ellipsoids are set at a 50% probability level. Hydrogen atoms are omitted for clarity; Figure S13. Ellipsoid representation of [Yb⊂{Au_3_(L1^ethyl^)_3_}] (**13**) including the positional disorder in the peripheral ethyl groups. The thermal ellipsoids are set at a 50% probability level. Hydrogen atoms are omitted for clarity. A solvent mask was calculated and 33 electrons were found in a volume of 235 A^3^ in 3 voids per unit cell. This is consistent with the presence of 1.5 H_2_O per formula unit which account for 30 electrons per unit cell; Figure S14. Ellipsoid representation of [Lu⊂{Au_3_(L1^ethyl^)_3_}] (**14**). The thermal ellipsoids are set at a 50% probability level. Hydrogen atoms are omitted for clarity; Figure S15. Ellipsoid representation of [Sc⊂{Au_3_(L1^ethyl^)_3_}] (**15**) including the positional disorder in the peripheral ethyl groups. The thermal ellipsoids are set at a 50% probability level. Hydrogen atoms are omitted for clarity; Figure S16. Ellipsoid representation of [Y⊂{Au_3_(L1^ethyl^)_3_}] (**16**) × H_2_O. The thermal ellipsoids are set at a 50% probability level. Hydrogen atoms are omitted for clarity; Figure S17: Ellipsoid representation of [In⊂{Au_3_(L1^ethyl^)_3_}] (**17**) including the positional disorder in the peripheral ethyl groups. The thermal ellipsoids are set at a 50% probability level. Hydrogen atoms are omitted for clarity; Figure S18. Ellipsoid representation of [Ga⊂{Au_2_(L1^ethyl^)_2_}](NO_3_) (**18a**). The thermal ellipsoids are set at a 50% probability level. Hydrogen atoms are omitted for clarity. A solvent mask was calculated and 334 electrons were found in a volume of 2100 A^3^ in 1 void per unit cell. This is consistent with the presence of 2 CH_2_Cl_2_ per formula unit which account for 336 electrons per unit cell; Figure S19. Ellipsoid representation of [Ga⊂{Au_2_(L1^ethyl^)_2_}](BF_4_) (**18b**). The thermal ellipsoids are set at a 50% probability level. Hydrogen atoms are omitted for clarity; Figure S20. Ellipsoid representation of [(AuCl)_2_(H_2_L1^ethyl^)] (**19**). The thermal ellipsoids are set at a 50% probability level. Hydrogen atoms are omitted for clarity; Figure S21. Ellipsoid representation of [Zn⊂{Au_2_(L1^ethyl^)_2_}] (**21**). The thermal ellipsoids are set at a 50% probability level. Hydrogen atoms are omitted for clarity. A solvent mask was calculated and 220 electrons were found in a volume of 946 A^3^ in 1 void per unit cell. This is consistent with the presence of 6 H_2_O per formula unit which account for 240 electrons per unit cell; Figure S22. Ellipsoid representation of [Sc(H_2_O)_2_{Au(L1^ethyl^)_2_}] (**22**) including the positional disorder in the peripheral ethyl groups. The thermal ellipsoids are set at a 50% probability level. Hydrogen atoms are omitted for clarity; Figure S23. IR (KBr) spectrum of [La⊂{Au_3_(L1^ethyl^)_3_}] (**1**); Figure S24. ^1^H NMR spectrum of [La⊂{Au_3_(L1^ethyl^)_3_}] (**1**) in CDCl_3_; Figure S25. ^13^C NMR spectrum of [La⊂{Au_3_(L1^ethyl^)_3_}] (**1**) in CDCl_3_; Figure S26. ESI+ MS spectrum of [La⊂{Au_3_(L1^ethyl^)_3_}] (**1**); Figure S27. IR (KBr) spectrum of [Ce⊂{Au_3_(L1^ethyl^)_3_}] (**2**); Figure S28. ESI+ MS spectrum of [Ce⊂{Au_3_(L1^ethyl^)_3_}] (**2**); Figure S29. IR (ATR) spectrum of [Pr⊂{Au_3_(L1^ethyl^)_3_}] (**3**); Figure S30. ESI+ MS spectrum of [Pr⊂{Au_3_(L1^ethyl^)_3_}] (**3**); Figure S31. IR (KBr) spectrum of [Nd⊂{Au_3_(L1^ethyl^)_3_}] (**4**); Figure S32. ESI+ MS spectrum of [Nd⊂{Au_3_(L1^ethyl^)_3_}] (**4**); Figure S33. IR (KBr) spectrum of [Sm⊂{Au_3_(L1^ethyl^)_3_}] (**5**); Figure S34. ESI+ MS spectrum of [Sm⊂{Au_3_(L1^ethyl^)_3_}] (**5**); Figure S35. IR (KBr) spectrum of [Eu⊂{Au_3_(L1^ethyl^)_3_}] (**6**); Figure S36. ESI+ MS spectrum of [Eu⊂{Au_3_(L1^ethyl^)_3_}] (**6**); Figure S37. IR (KBr) spectrum of [Gd⊂{Au_3_(L1^ethyl^)_3_}] (**7**); Figure S38. ESI+ MS spectrum of [Gd⊂{Au_3_(L1^ethyl^)_3_}] (**7**); Figure S39. IR (ATR) spectrum of [Tb⊂{Au_3_(L1^ethyl^)_3_}] (**8**); Figure S40. ESI+ MS spectrum of [Tb⊂{Au_3_(L1^ethyl^)_3_}] (**8**); Figure S41. IR (KBr) spectrum of [Dy⊂{Au_3_(L1^ethyl^)_3_}] (**9**); Figure S42. ESI+ MS spectrum of [Dy⊂{Au_3_(L1^ethyl^)_3_}] (**9**); Figure S43. IR (ATR) spectrum of [Ho⊂{Au_3_(L1^ethyl^)_3_}] (**10**); Figure S44. ESI+ MS spectrum of [Ho⊂{Au_3_(L1^ethyl^)_3_}] (**10**); Figure S45. IR (KBr) spectrum of [Er⊂{Au_3_(L1^ethyl^)_3_}] (**11**); Figure S46. ESI+ MS spectrum of [Er⊂{Au_3_(L1^ethyl^)_3_}] (**11**); Figure S47. IR (ATR) spectrum of [Tm⊂{Au_3_(L1^ethyl^)_3_}] (**12**); Figure S48. ESI+ MS spectrum of [Tm⊂{Au_3_(L1^ethyl^)_3_}] (**12**); Figure S49. IR (KBr) spectrum of [Yb⊂{Au_3_(L1^ethyl^)_3_}] (**13**); Figure S50. ESI+ MS spectrum of [Yb⊂{Au_3_(L1^ethyl^)_3_}] (**13**); Figure S51. IR (KBr) spectrum of [Lu⊂{Au_3_(L1^ethyl^)_3_}] (**14**); Figure S52. ESI+ MS spectrum of [Lu⊂{Au_3_(L1^ethyl^)_3_}] (**14**); Figure S53. IR (KBr) spectrum of [Sc⊂{Au_3_(L1^ethyl^)_3_}] (**15**); Figure S54. ESI+ MS spectrum of [Sc⊂{Au_3_(L1^ethyl^)_3_}] (**15**); Figure S55. ^1^H NMR spectrum of [Sc⊂{Au_3_(L1^ethyl^)_3_}] (**15**) in CDCl_3_; Figure S56. ^13^C NMR spectrum of [Sc⊂{Au_3_(L1^ethyl^)_3_}] (**15**) in CDCl_3_; Figure S57. ^45^Sc NMR spectrum of [Sc⊂{Au_3_(L1^ethyl^)_3_}] (**15**) in CD_2_Cl_2_; Figure S58. IR (ATR) spectrum of [Y⊂{Au_3_(L1^ethyl^)_3_}] (**16**); Figure S59. ESI MS spectrum of [Y⊂{Au_3_(L1^ethyl^)_3_}] (**16**); Figure S60. ^1^H NMR spectrum of [Y⊂{Au_3_(L1^ethyl^)_3_}] (**16**) in CDCl_3_; Figure S61. IR (KBr) spectrum of [In⊂{Au_3_(L1^ethyl^)_3_}] (**17**); Figure S62. ESI+ MS spectrum of [In⊂{Au_3_(L1^ethyl^)_3_}] (**17**); Figure S63. ^1^H NMR spectrum of [In⊂{Au_3_(L1^ethyl^)_3_}] (**17**) in CDCl_3_; Figure S64. ^13^C NMR spectrum of [In⊂{Au_3_(L1^ethyl^)_3_}] (**17**) in CDCl_3_; Figure S65. IR (KBr) spectrum of [Ga⊂{Au_2_(L1^ethyl^)_2_}](NO_3_) (**18a**); Figure S66. ESI+ MS spectrum of [Ga⊂{Au_2_(L1^ethyl^)_2_}](NO_3_) (**18a**); Figure S67. ^1^H NMR spectrum of [Ga⊂{Au_2_(L1^ethyl^)_2_}](NO_3_) (**18a**) in DMSO-D_6_; Figure S68. IR (KBr) spectrum of [{AuCl}_2_(H_2_L1^ethyl^)] (**19**); Figure S69. ESI MS spectrum of [{AuCl}_2_(H_2_L1^ethyl^)] (**19**); Figure S70. ^1^H NMR spectrum of [{AuCl}_2_(H_2_L1^ethyl^)] (**19**). in CDCl_3_; Figure S71. ^13^C NMR spectrum of [{AuCl}_2_(H_2_L1^ethyl^)] (**19**). in CDCl_3_; Figure S72. IR (KBr) spectrum of [{Au(PPh_3_}_2_(L1^ethyl^)] (**20**); Figure S73. ESI+ MS spectrum of [{Au(PPh_3_}_2_(L1^ethyl^)] (**20**); Figure S74. IR (KBr) spectrum of [Zn⊂{Au_2_(L1^ethyl^)_2_}] (**21**); Figure S75. ESI+ MS spectrum of [Zn⊂{Au_2_(L1^ethyl^)_2_}] (**21**).
