# Supplementary material for: Gold-Based Coronands as Hosts for M3+ Metal Ions: Ring Size Matters
Source: Molecules. 2023 Jul 14;28(14):5421. doi: 10.3390/molecules28145421 (PMC10385047; doi:10.3390/molecules28145421)
Supplement: Supplementary file 1 [file molecules-28-05421-s001.zip › molecules-2499508-supplementary.pdf]

Supporting Information of the paper entitled:

Gold-based Coronands as Hosts for M^{3+} Metal ions: Ring Size Matters

Suelen Ferreira Sucena [1], Türkan Ilgin Demirer [1], Anna Baitullina [1], Adelheid Hagenbach [1], Jacqueline Grewe [1], Sarah Spreckelmeyer [2], Juliane März [3], Astrid Barkleit [3], Pedro Ivo da S. Maia [4], Hung Huy Nguyen [5], and Ulrich Abram [1*]

[1] Institute of Chemistry and Biochemistry, Freie Universität Berlin, Fabeckstr. 34/36, 14195 Berlin, Germany.

[2] Department of Nuclear Medicine and Berlin Institute of Health, Charité - Universitätsmedizin Berlin, corporate member of Freie Universität Berlin, Humboldt-Universität zu Berlin, Augustenburger Platz 1, 13353, Berlin, Germany.

[3] Institute of Resource Ecology, Helmholtz-Zentrum Dresden-Rossendorf (HZDR), Bautzner Landstr. 400, 01328 Dresden, Germany.

[4] Núcleo de Desenvolvimento de Compostos Bioativos (NDCBio), Universidade Federal do Triângulo Mineiro, Av. Dr. Randolfo Borges 1400, 38025-440 Uberaba, MG, Brazil.

[5] Department of Inorganic Chemistry, VNU University of Science, 19 Le Thanh Tong, Hanoi, Vietnam.

Table of content

Crystallographic data 6

Table S1: Crystallographic data and data collection parameters 6

Table S1: Crystallographic data and data collection parameters (continued) 7

Table S1: Crystallographic data and data collection parameters (continued) 8

Table S1: Crystallographic data and data collection parameters (continued) 9

Table S1: Crystallographic data and data collection parameters (continued) 10

Table S1: Crystallographic data and data collection parameters (continued) 11

Table S1: Crystallographic data and data collection parameters (continued) 12

Table S1: Crystallographic data and data collection parameters (continued) 13

Figure S1. Ellipsoid representation of [La⊂{Au_3(L1^{ethyl})$_3$}] (**1**) including the positional disorder in the peripheral ethyl groups. The thermal ellipsoids are set at a 50% probability level. Hydrogen atoms are omitted for clarity. A solvent mask was calculated and 53 electrons were found in a volume of 218 A^3 in 3 voids per unit cell. This is consistent with the presence of 2.5 H_2O per formula unit which account for 50 electrons per unit cell. 14

Figure S2. Ellipsoid representation of [Ce⊂{Au_3(L1^{ethyl})$_3$}] (**2**) including the positional disorder in the peripheral ethyl groups. The thermal ellipsoids are set at a 50% probability level. Hydrogen atoms are omitted for clarity. 14

Figure S3: Ellipsoid representation of [Pr⊂{Au_3(L1^{ethyl})$_3$}] (**3**) including the positional disorder in the peripheral ethyl groups. The thermal ellipsoids are set at a 50% probability level. Hydrogen atoms are omitted for clarity. 15

Figure S4: Ellipsoid representation of [Nd⊂{Au_3(L1^{ethyl})$_3$}] (**4**) including the positional disorder in the peripheral ethyl groups. The thermal ellipsoids are set at a 50% probability level. Hydrogen atoms are omitted for clarity. A solvent mask was calculated and 54 electrons were found in a volume of 247 A^3 in 4 voids per unit cell. This is consistent with the presence of 2.5 H_2O per formula unit which account for 50 electrons per unit cell. 15

Figure S5: Ellipsoid representation of [Sm⊂{Au_3(L1^{ethyl})$_3$}] (**5**). The thermal ellipsoids are set at a 50% probability level. Hydrogen atoms are omitted for clarity. A solvent mask was calculated and 55 electrons were found in a volume of 240 A^3 in 4 voids per unit cell. This is consistent with the presence of 2.5 H_2O per formula unit which account for 50 electrons per unit cell. 16

Figure S6: Ellipsoid representation of [Eu⊂{Au_3(L1^{ethyl})$_3$}] (**6**). The thermal ellipsoids are set at a 50% probability level. Hydrogen atoms are omitted for clarity. A solvent mask was calculated and 46 electrons were found in a volume of 227 A^3 in 3 voids per unit cell. This is consistent with the presence of 2.5 H_2O per formula unit which account for 50 electrons per unit cell. 16

Figure S7: Ellipsoid representation of [Gd⊂{Au_3(L1^{ethyl})$_3$}] (**7**). The thermal ellipsoids are set at a 50% probability level. Hydrogen atoms are omitted for clarity. A solvent mask was calculated and 65 electrons were found in a volume of 179 A^3 in 2 voids per unit cell. This is consistent with the presence of 3.5 H_2O per formula unit which account for 70 electrons per unit cell. 17

Figure S8: Ellipsoid representation of [Tb⊂{Au_3($L1^{ethyl}$)$_3$}] (**8**) including the positional disorder in the peripheral ethyl groups. The thermal ellipsoids are set at a 50% probability level. Hydrogen atoms are omitted for clarity. A solvent mask was calculated and 48 electrons were found in a volume of 252 A^3 in 3 voids per unit cell. This is consistent with the presence of 2.5 H_2O per formula unit which account for 50 electrons per unit cell. 17

Figure S9: Ellipsoid representation of [Dy⊂{Au_3($L1^{ethyl}$)$_3$}] (**9**). The thermal ellipsoids are set at a 50% probability level. Hydrogen atoms are omitted for clarity. A solvent mask was calculated and 43 electrons were found in a volume of 138 A^3 in 1 void per unit cell. This is consistent with the presence of 2 H_2O per formula unit which account for 40 electrons per unit cell. 18

Figure S10: Ellipsoid representation of [Ho⊂{Au_3($L1^{ethyl}$)$_3$}] (**10**) including the positional disorder in the peripheral ethyl groups. The thermal ellipsoids are set at a 50% probability level. Hydrogen atoms are omitted for clarity. A solvent mask was calculated and 43 electrons were found in a volume of 282 A^3 in 3 voids per unit cell. This is consistent with the presence of 2 H_2O per formula unit which account for 40 electrons per unit cell. 18

Figure S11: Ellipsoid representation of [Er⊂{Au_3($L1^{ethyl}$)$_3$}] (**11**) including the positional disorder in the peripheral N(ethyl)$_2$ groups. The thermal ellipsoids are set at a 50% probability level. Hydrogen atoms are omitted for clarity. 19

Figure S12: Ellipsoid representation of [Tm⊂{Au_3($L1^{ethyl}$)$_3$}] (**12**) including the positional disorder in the peripheral N(ethyl)$_2$ groups. The thermal ellipsoids are set at a 50% probability level. Hydrogen atoms are omitted for clarity. 19

Figure S13: Ellipsoid representation of [Yb⊂{Au_3($L1^{ethyl}$)$_3$}] (**13**) including the positional disorder in the peripheral ethyl groups. The thermal ellipsoids are set at a 50% probability level. Hydrogen atoms are omitted for clarity. A solvent mask was calculated and 33 electrons were found in a volume of 235 A^3 in 3 voids per unit cell. This is consistent with the presence of 1.5 H_2O per formula unit which account for 30 electrons per unit cell. 20

Figure S14: Ellipsoid representation of [Lu⊂{Au_3($L1^{ethyl}$)$_3$}] (**14**). The thermal ellipsoids are set at a 50% probability level. Hydrogen atoms are omitted for clarity. 20

Figure S15: Ellipsoid representation of [Sc⊂{Au_3($L1^{ethyl}$)$_3$}] (**15**) including the positional disorder in the peripheral ethyl groups. The thermal ellipsoids are set at a 50% probability level. Hydrogen atoms are omitted for clarity. 21

Figure S16: Ellipsoid representation of [Y⊂{Au_3($L1^{ethyl}$)$_3$}] (**16**) x H_2O. The thermal ellipsoids are set at a 50% probability level. Hydrogen atoms are omitted for clarity. 21

Figure S17: Ellipsoid representation of [In⊂{Au_3($L1^{ethyl}$)$_3$}] (**17**) including the positional disorder in the peripheral ethyl groups. The thermal ellipsoids are set at a 50% probability level. Hydrogen atoms are omitted for clarity. 22

Figure S18: Ellipsoid representation of [Ga⊂{Au_2($L1^{ethyl}$)$_2$}](NO_3) (**18a**). The thermal ellipsoids are set at a 50% probability level. Hydrogen atoms are omitted for clarity. A solvent mask was calculated and 334 electrons were found in a volume of 2100 A^3 in 1 void per unit cell. This is consistent with the presence of 2 CH_2Cl_2 per formula unit which account for 336 electrons per unit cell. 22

Figure S19: Ellipsoid representation of [Ga⊂{Au_2($L1^{ethyl}$)$_2$}](BF_4) (**18b**). The thermal ellipsoids are set at a 50% probability level. Hydrogen atoms are omitted for clarity. 23

Figure S20: Ellipsoid representation of [$(AuCl)_2(H_2L1^{ethyl})$] (**19**). The thermal ellipsoids are set at a 50% probability level. Hydrogen atoms are omitted for clarity. 23

Figure S21: Ellipsoid representation of [Zn⊂{$Au_2(L1^{ethyl})_2$}] (**21**). The thermal ellipsoids are set at a 50% probability level. Hydrogen atoms are omitted for clarity. A solvent mask was calculated and 220 electrons were found in a volume of 946 A^3 in 1 void per unit cell. This is consistent with the presence of 6 H_2O per formula unit which account for 240 electrons per unit cell. 24

Figure S22: Ellipsoid representation of [Sc$(H_2O)_2${Au$(L1^{ethyl})_2$}] (**22**) including the positional disorder in the peripheral ethyl groups. The thermal ellipsoids are set at a 50% probability level. Hydrogen atoms are omitted for clarity. 24

Spectroscopic data 25

Figure S23: IR (KBr) spectrum of [La⊂{$Au_3(L1^{ethyl})_3$}] (**1**). 25

Figure S24: ^{1}H NMR spectrum of [La⊂{$Au_3(L1^{ethyl})_3$}] (**1**) in $CDCl_3$. 25

Figure S25: ^{13}C NMR spectrum of [La⊂{$Au_3(L1^{ethyl})_3$}] (**1**) in $CDCl_3$. 26

Figure S26: ESI+ MS spectrum of [La⊂{$Au_3(L1^{ethyl})_3$}] (**1**). 26

Figure S27: IR (KBr) spectrum of [Ce⊂{$Au_3(L1^{ethyl})_3$}] (**2**). 27

Figure S28: ESI+ MS spectrum of [Ce⊂{$Au_3(L1^{ethyl})_3$}] (**2**). 27

Figure S29: IR (ATR) spectrum of [Pr⊂{$Au_3(L1^{ethyl})_3$}] (**3**). 28

Figure S30: ESI+ MS spectrum of [Pr⊂{$Au_3(L1^{ethyl})_3$}] (**3**). 28

Figure S31: IR (KBr) spectrum of [Nd⊂{$Au_3(L1^{ethyl})_3$}] (**4**). 29

Figure S32: ESI+ MS spectrum of [Nd⊂{$Au_3(L1^{ethyl})_3$}] (**4**). 29

Figure S33: IR (KBr) spectrum of [Sm⊂{$Au_3(L1^{ethyl})_3$}] (**5**). 30

Figure S34: ESI+ MS spectrum of [Sm⊂{$Au_3(L1^{ethyl})_3$}] (**5**). 30

Figure S35: IR (KBr) spectrum of [Eu⊂{$Au_3(L1^{ethyl})_3$}] (**6**). 31

Figure S36: ESI+ MS spectrum of [Eu⊂{$Au_3(L1^{ethyl})_3$}] (**6**). 31

Figure S37: IR (KBr) spectrum of [Gd⊂{$Au_3(L1^{ethyl})_3$}] (**7**). 32

Figure S38: ESI+ MS spectrum of [Gd⊂{$Au_3(L1^{ethyl})_3$}] (**7**). 32

Figure S39: IR (ATR) spectrum of [Tb⊂{$Au_3(L1^{ethyl})_3$}] (**8**). 33

Figure S40: ESI+ MS spectrum of [Tb⊂{$Au_3(L1^{ethyl})_3$}] (**8**). 33

Figure S41: IR (KBr) spectrum of [Dy⊂{$Au_3(L1^{ethyl})_3$}] (**9**). 34

Figure S42: ESI+ MS spectrum of [Dy⊂{$Au_3(L1^{ethyl})_3$}] (**9**). 34

Figure S43: IR (ATR) spectrum of [Ho⊂{$Au_3(L1^{ethyl})_3$}] (**10**). 35

Figure S44: ESI+ MS spectrum of [Ho⊂{$Au_3(L1^{ethyl})_3$}] (**10**). 35

Figure S45: IR (KBr) spectrum of [Er⊂{$Au_3(L1^{ethyl})_3$}] (**11**). 36

Figure S46: ESI+ MS spectrum of [Er⊂{$Au_3(L1^{ethyl})_3$}] (**11**). 36

Figure S47: IR (ATR) spectrum of [Tm⊂{$Au_3(L1^{ethyl})_3$}] (**12**). 37

Figure S48: ESI+ MS spectrum of [Tm⊂{$Au_3(L1^{ethyl})_3$}] (**12**). 37

Figure S49: IR (KBr) spectrum of [Yb⊂{$Au_3(L1^{ethyl})_3$}] (**13**). 38

Figure S50: ESI+ MS spectrum of [Yb⊂{Au_3(L1^{ethyl})$_3$}] (**13**). 38
Figure S51: IR (KBr) spectrum of [Lu⊂{Au_3(L1^{ethyl})$_3$}] (**14**). 39
Figure S52: ESI+ MS spectrum of [Lu⊂{Au_3(L1^{ethyl})$_3$}] (**14**). 39
Figure S53: IR (KBr) spectrum of [Sc⊂{Au_3(L1^{ethyl})$_3$}] (**15**). 40
Figure S54: ESI+ MS spectrum of [Sc⊂{Au_3(L1^{ethyl})$_3$}] (**15**). 40
Figure S55: ^{1}H NMR spectrum of [Sc⊂{Au_3(L1^{ethyl})$_3$}] (**15**) in $CDCl_3$. 41
Figure S56: ^{13}C NMR spectrum of [Sc⊂{Au_3(L1^{ethyl})$_3$}] (**15**) in $CDCl_3$ 41
Figure S57: ^{45}Sc NMR spectrum of [Sc⊂{Au_3(L1^{ethyl})$_3$}] (**15**) in CD_2Cl_2. 42
Figure S58: IR (ATR) spectrum of [Y⊂{Au_3(L1^{ethyl})$_3$}] (**16**). 42
Figure S59: ESI MS spectrum of [Y⊂{Au_3(L1^{ethyl})$_3$}] (**16**). 43
Figure S60: ^{1}H NMR spectrum of [Y⊂{Au_3(L1^{ethyl})$_3$}] (**16**) in $CDCl_3$. 43
Figure S61: IR (KBr) spectrum of [In⊂{Au_3(L1^{ethyl})$_3$}] (**17**). 44
Figure S62: ESI+ MS spectrum of [In⊂{Au_3(L1^{ethyl})$_3$}] (**17**). 44
Figure S63: ^{1}H NMR spectrum of [In⊂{Au_3(L1^{ethyl})$_3$}] (**17**) in $CDCl_3$. 45
Figure S64: ^{13}C NMR spectrum of [In⊂{Au_3(L1^{ethyl})$_3$}] (**17**) in $CDCl_3$. 45
Figure S65: IR (KBr) spectrum of [Ga⊂{Au_2(L1^{ethyl})$_2$}](NO_3) (**18a**). 46
Figure S66: ESI+ MS spectrum of [Ga⊂{Au_2(L1^{ethyl})$_2$}](NO_3) (**18a**). 46
Figure S67: ^{1}H NMR spectrum of [Ga⊂{Au_2(L1^{ethyl})$_2$}](NO_3) (**18a**) in DMSO-D_6. 47
Figure S68: IR (KBr) spectrum of [{AuCl}$_2$(H_2L1^{ethyl})] (**19**). 47
Figure S69: ESI MS spectrum of [{AuCl}$_2$(H_2L1^{ethyl})] (**19**). 48
Figure S70: ^{1}H NMR spectrum of [{AuCl}$_2$(H_2L1^{ethyl})] (**19**). in $CDCl_3$. 48
Figure S71: ^{13}C NMR spectrum of [{AuCl}$_2$(H_2L1^{ethyl})] (**19**). in $CDCl_3$. 49
Figure S72: IR (KBr) spectrum of [{Au(PPh_3}$_2$(L1^{ethyl})] (**20**). 49
Figure S73: ESI+ MS spectrum of [{Au(PPh_3}$_2$(L1^{ethyl})] (**20**). 50
Figure S74: IR (KBr) spectrum of [Zn⊂{Au_2(L1^{ethyl})$_2$}] (**21**). 50
Figure S75: ESI+ MS spectrum of [Zn⊂{Au_2(L1^{ethyl})$_2$}] (**21**). 51

Crystallographic data

Table S1: Crystallographic data and data collection parameters

	[La⊂{Au_3(L1^{ethyl})$_3$}] (**1**)	[Ce⊂{Au_3(L1^{ethyl})$_3$}] (**2**)	[Pr⊂{Au_3(L1^{ethyl})$_3$}] (**3**)
Empirical formula	$C_{51}H_{69}Au_3LaN_{15}O_6S_6$	$C_{51}H_{69}Au_3CeN_{15}O_6S_6$	$C_{51}H_{69}Au_3PrN_{15}O_6S_6$
Formula weight	1910.38	1911.59	1912.38
Temperature/K	100	150	200
Crystal system	Trigonal	Trigonal	Trigonal
Space group	P-3	P-3	P-3
a/Å	16.412(1)	16.3263(5)	16.460(2)
b/Å	16.412(1)	16.3263(5)	16.460(2)
c/Å	14.592(8)	14.6155(5)	14.671(8)
α/°	90	90	90
β/°	90	90	90
γ/°	120	120	120
Volume/Å^3	3403(2)	3373.8(2)	3442(1)
Z	2	2	2
ρ_{calc}g / cm^3	1.864	1.882	1.845
μ / mm^{-1}	7.298	7.405	7.304
F(000)	1836.0	1838.0	1840.0
Crystal size / mm^3	0.45 × 0.17 × 0.08	0.15 × 0.11 × 0.10	0.20 × 0.20 × 0.15
Radiation	MoKα (λ = 0.71073)	MoKα (λ = 0.71073)	MoKα (λ = 0.71073)
2Θ range for data collection/°	5.698 to 49.984	5.574 to 55.812	7.442 to 50.99
Index ranges	-19 ≤ h ≤ 17, -19 ≤ k ≤ 19, -14 ≤ l ≤ 17	-21 ≤ h ≤ 21, -21 ≤ k ≤ 21, -19 ≤ l ≤ 19	-19 ≤ h ≤ 11, -18 ≤ k ≤ 18, -15 ≤ l ≤ 17
Reflections collected	18962	29918	7498
Independent reflections	3992 [R_{int} = 0.0540, R_{sigma} = 0.0388]	5198 [R_{int} = 0.0712, R_{sigma} = 0.0505]	4033 [R_{int} = 0.1019, R_{sigma} = 0.1447]
Data/restraints/parameters	3992/0/214	5198/11/216	4033/10/238
Goodness-of-fit on F^2	1.047	1.034	0.874
Final R indexes [I>=2σ (I)]	R_1 = 0.0455, wR_2 = 0.1077	R_1 = 0.0485, wR_2 = 0.1126	R_1 = 0.0644, wR_2 = 0.1462
Final R indexes [all data]	R_1 = 0.0557, wR_2 = 0.1169	R_1 = 0.0679, wR_2 = 0.1220	R_1 = 0.1225, wR_2 = 0.1653
Largest diff. peak/hole / e Å^{-3}	2.12/-3.35	2.39/-2.17	1.53/-2.01
Diffractometer	Bruker APEX	Bruker APEX	STOE IPDS
CCDC access code	2269218	2269219	2269220

Table S1: Crystallographic data and data collection parameters (continued)

	[Nd⊂{Au_3(L1^{ethyl})$_3$}] (**4**)	[Sm⊂{Au_3(L1^{ethyl})$_3$}] (**5**)	[Eu⊂{Au_3(L1^{ethyl})$_3$}] (**6**)
Empirical formula	$C_{51}H_{69}Au_3NdN_{15}O_6S_6$	$C_{51}H_{69}Au_3SmN_{15}O_6S_6$	$C_{51}H_{69}Au_3EuN_{15}O_6S_6$
Formula weight	1915.71	1921.82	1923.43
Temperature/K	100	100	100
Crystal system	Trigonal	Trigonal	Trigonal
Space group	P-3	P-3	P-3
a/Å	16.354(2)	16.2775(8)	16.251(2)
b/Å	16.354(2)	16.2775(8)	16.251(2)
c/Å	14.621(1)	14.5918(6)	14.599(1)
α/°	90	90	90
β/°	90	90	90
γ/°	120	120	120
Volume/Å^3	3386.5(9)	3403(2)	3338.9(7)
Z	2	2	2
ρ_{calc}g / cm^3	1.842	1.906	1.913
μ / mm^{-1}	7.471	7.658	7.740
F(000)	1842.0	1846.0	1848.0
Crystal size / mm^3	0.35 × 0.06 × 0.05	0.22 × 0.15 × 0.06	0.25 × 0.08 × 0.05
Radiation	MoKα (λ = 0.71073)	MoKα (λ = 0.71073)	MoKα (λ = 0.71073)
2Θ range for data collection/°	5.572 to 49.954	5.584 to 49.000	5.014 to 48.988
Index ranges	-19 ≤ h ≤ 19, -19 ≤ k ≤ 19, -17 ≤ l ≤ 15	-18 ≤ h ≤ 18, -18 ≤ k ≤ 18, -17 ≤ l ≤ 17	-18 ≤ h ≤ 16, -18 ≤ k ≤ 18, -17 ≤ l ≤ 13
Reflections collected	19758	29390	15727
Independent reflections	3979 [R_{int} = 0.0489, R_{sigma} = 0.0332]	3694 [R_{int} = 0.0607, R_{sigma} = 0.0328]	3631 [R_{int} = 0.0402, R_{sigma} = 0.0344]
Data/restraints/parameters	3979/3/262	3694/10/189	3631/2/195
Goodness-of-fit on F^2	1.130	1.198	1.208
Final R indexes [I>=2σ (I)]	R_1 = 0.0393, wR_2 = 0.0678	R_1 = 0.0496, wR_2 = 0.1037	R_1 = 0.0569, wR_2 = 0.1256
Final R indexes [all data]	R_1 = 0.0529, wR_2 = 0.0720	R_1 = 0.0595, wR_2 = 0.1086	R_1 = 0.0668, wR_2 = 0.1336
Largest diff. peak/hole / e Å^{-3}	2.20/-2.27	2.82/-2.71	2.80/-2.68
Diffractometer	Bruker APEX	Bruker APEX	Bruker APEX
CCDC access code	2269221	2269222	2269223

Table S1: Crystallographic data and data collection parameters (continued)

	[Gd⊂{Au_3(L1^{ethyl})$_3$}] (**7**)	[Tb⊂{Au_3(L1^{ethyl})$_3$}] (**8**)	[Dy⊂{Au_3(L1^{ethyl})$_3$}] (**9**)
Empirical formula	$C_{51}H_{69}Au_3GdN_{15}O_6S_6$	$C_{51}H_{69}Au_3TbN_{15}O_6S_6$	$C_{51}H_{69}Au_3DyN_{15}O_6S_6$
Formula weight	1928.72	1930.39	1933.97
Temperature/K	100	293	133
Crystal system	Trigonal	Trigonal	Trigonal
Space group	P-3	P-3	P-3
a/Å	16.250(1)	16.236(1)	16.212(1)
b/Å	16.250(1)	16.236(1)	16.212(1)
c/Å	14.596(1)	14.588(8)	14.601(1)
α/°	90	90	90
β/°	90	90	90
γ/°	120	120	120
Volume/Å^3	3337.9(5)	3330.1(5)	3323.4(5)
Z	2	2	2
ρ_{calc}g / cm^3	1.919	1.925	1.864
μ / mm^{-1}	7.796	7.880	7.954
F(000)	1850.0	1852.0	1864.0
Crystal size / mm^3	0.26 × 0.11 × 0.05	0.26 × 0.12 × 0.08	0.29 × 0.13 × 0.08
Radiation	MoKα (λ = 0.71073)	MoKα (λ = 0.71073)	MoKα (λ = 0.71073)
2Θ range for data collection/°	5.014 to 48.996	5.018 to 49.976	5.58 to 48.988
Index ranges	-18 ≤ h ≤ 18, -18 ≤ k ≤ 18, -16 ≤ l ≤ 17	-19 ≤ h ≤ 19, -19 ≤ k ≤ 19, -17 ≤ l ≤ 17	-18 ≤ h ≤ 18, -18 ≤ k ≤ 18, -17 ≤ l ≤ 17
Reflections collected	56033	62999	25421
Independent reflections	3708 [R_{int} = 0.0472, R_{sigma} = 0.0186]	3905 [R_{int} = 0.0407, R_{sigma} = 0.0146]	3652 [R_{int} = 0.0668, R_{sigma} = 0.0396]
Data/restraints/parameters	3708/4/219	3905/1/258	3652/4/219
Goodness-of-fit on F^2	1.214	1.107	1.214
Final R indexes [I>=2σ (I)]	R_1 = 0.0395, wR_2 = 0.0829	R_1 = 0.0217, wR_2 = 0.0486	R_1 = 0.0560, wR_2 = 0.1165
Final R indexes [all data]	R_1 = 0.0429, wR_2 = 0.0845	R_1 = 0.0235, wR_2 = 0.0496	R_1 = 0.0671, wR_2 = 0.1207
Largest diff. peak/hole / e Å^{-3}	2.12/-3.35	1.09/-2.21	2.91/-2.83
Diffractometer	Bruker APEX	Bruker APEX	Bruker APEX
CCDC access code	2269224	2269225	2269226

Table S1: Crystallographic data and data collection parameters (continued)

	[Ho⊂{$Au_3(L1^{ethyl})_3$}] (**10**)	[Er⊂{$Au_3(L1^{ethyl})_3$}] (**11**)	[Tm⊂{$Au_3(L1^{ethyl})_3$}] (**12**)
Empirical formula	$C_{51}H_{69}Au_3HoN_{15}O_6S_6$	$C_{51}H_{69}Au_3ErN_{15}O_6S_6$	$C_{51}H_{69}Au_3TmN_{15}O_6S_6$
Formula weight	1936.40	1938.73	1940.40
Temperature/K	100	200	100
Crystal system	Trigonal	Trigonal	Monoclinic
Space group	P-3	P-3	C2/c
a/Å	16.2209(9)	16.344(5)	22.780(2)
b/Å	16.2209(9)	16.344(5)	13.556(1)
c/Å	14.592(8)	14.835(4)	20.961(2)
α/°	90	90	90
β/°	90	90	96.340(3)
γ/°	120	120	90
Volume/$Å^3$	3332.1(4)	3432(2)	6433.2(9)
Z	2	2	4
ρ_{calc}g / cm^3	1.930	1.876	2.003
μ / mm^{-1}	8.002	7.839	8.438
F(000)	1856.0	1858.0	3720.0
Crystal size / mm^3	0.19 × 0.17 × 0.09	0.20 × 0.20 × 0.15	0.23 × 0.23 × 0.15
Radiation	MoKα (λ = 0.71073)	MoKα (λ = 0.71073)	MoKα (λ = 0.71073)
2Θ range for data collection/°	5.022 to 54.356	7.42 to 51.00	5.014 to 54.306
Index ranges	-20 ≤ h ≤ 20, -20 ≤ k ≤ 20, -18 ≤ l ≤ 18	-19 ≤ h ≤ 12, -19 ≤ k ≤ 19, -17 ≤ l ≤ 17	-29 ≤ h ≤ 29, -17 ≤ k ≤ 17, -26 ≤ l ≤ 26
Reflections collected	79051	14646	124085
Independent reflections	4926[R_{int} = 0.0630, R_{sigma} = 0.0235]	4254 [R_{int} = 0.1111, R_{sigma} = 0.0992]	7104 [R_{int} = 0.0813, R_{sigma} = 0.0267]
Data/restraints/parameters	4926/4/256	4254/3/240	7104/38/369
Goodness-of-fit on F^2	1.217	0.845	1.033
Final R indexes [I>=2σ (I)]	R_1 = 0.0320, wR_2 = 0.0668	R_1 = 0.0386, wR_2 = 0.0606	R_1 = 0.0297, wR_2 = 0.0675
Final R indexes [all data]	R_1 = 0.0381, wR_2 = 0.0688	R_1 = 0.0755, wR_2 = 0.0766	R_1 = 0.0323, wR_2 = 0.0693
Largest diff. peak/hole / e $Å^{-3}$	1.64/-2.46	1.11/-1.00	2.57/-3.10
Diffractometer	Bruker APEX	STOE IPDS	Bruker APEX
CCDC access code	2269227	2269228	2269014

Table S1: Crystallographic data and data collection parameters (continued)

	[Yb⊂{Au_3(L1^{ethyl})$_3$}] (**13**)	[Lu⊂{Au_3(L1^{ethyl})$_3$}] (**14**)	[Sc⊂{Au_3(L1^{ethyl})$_3$}] (**15**)
Empirical formula	$C_{51}H_{69}Au_3YbN_{15}O_6S_6$	$C_{51}H_{69}Au_3LuN_{15}O_6S_6$	$C_{51}H_{69}Sc_3LaN_{15}O_6S_6$
Formula weight	1944.51	1946.44	1816.43
Temperature/K	100	293	100
Crystal system	Trigonal	Triclinic	Triclinic
Space group	P-3	P-1	P-1
a/Å	16.116(1)	13.652(1)	13.5162(9)
b/Å	16.116(1)	14.813(1)	14.7445(9)
c/Å	14.557(1)	16.537(1)	16.3531)
α/°	90	89.14(1)	89.303(2)
β/°	90	79.67(1)	79.801(2)
γ/°	120	79.83(1)	79.260(3)
Volume/Å^3	3274.2(6)	3237.9(4)	3150(3)
Z	2	2	2
ρ_{calc}g / cm^3	1.972	1.996	1.915
μ / mm^{-1}	8.363	8.537	7.324
F(000)	1836.0	1864.0	1764.0
Crystal size / mm^3	0.18 × 0.06 × 0.05	0.30 × 0.30 × 0.20	0.20 × 0.17 × 0.10
Radiation	MoKα (λ = 0.71073)	MoKα (λ = 0.71073)	MoKα (λ = 0.71073)
2Θ range for data collection/°	5.056 to 48.91	6.574 to 49.000	4.472 to 61.162
Index ranges	-18 ≤ h ≤ 18, -14 ≤ k ≤ 18, -16 ≤ l ≤ 16	-15 ≤ h ≤ 15, -17 ≤ k ≤ 17, -18 ≤ l ≤ 19	-19 ≤ h ≤ 19, -21 ≤ k ≤ 21, -23 ≤ l ≤ 23
Reflections collected	15100	21960	182794
Independent reflections	3608 [R_{int} = 0.0607, R_{sigma} = 0.0521]	10671 [R_{int} = 0.0908, R_{sigma} = 0.0767]	19345 [R_{int} = 0.0560, R_{sigma} = 0.0273]
Data/restraints/parameters	3608/35/193	10671/27/740	19345/0/739
Goodness-of-fit on F^2	1.164	1.005	1.059
Final R indexes [I>=2σ (I)]	R_1 = 0.0647, wR_2 = 0.1417	R_1 = 0.0436, wR_2 = 0.1060	R_1 = 0.0208, wR_2 = 0.0439
Final R indexes [all data]	R_1 = 0.0751, wR_2 = 0.1468	R_1 = 0.0535, wR_2 = 0.1108	R_1 = 0.0264, wR_2 = 0.0455
Largest diff. peak/hole / e Å^{-3}	4.09/-3.65	1.55/-2.51	0.93/-1.76
Diffractometer	Bruker APEX	STOE IPDS	Bruker APEX
CCDC access code	2269015	2269016	2269017

Table S1: Crystallographic data and data collection parameters (continued)

	$[Y\subset\{Au_3(L1^{ethyl})_3\}]$ x H_2O (**16**)	$[In\subset\{Au_3(L1^{ethyl})_3\}]$ (**17**)	$[Ga\subset\{Au_2(L1^{ethyl})_2\}](NO_3)$ (**18a**)
Empirical formula	$C_{51}H_{671}Au_3YN_{15}O_7S_6$	$C_{51}H_{69}Au_3InN_{15}O_6S_6$	$C_{34}H_{46}Au_2GaN_{11}O_7S_6$
Formula weight	1878.39	1886.29	1312.71
Temperature/K	293	100	100
Crystal system	Monoclinic	Monoclinic	Monoclinic
Space group	$P2_1/c$	C2/c	$P2_1/c$
a/Å	18.28(3)	22.773(2)	10.0200(7)
b/Å	18.13(1)	13.501(1)	19.210(1)
c/Å	22.69(4)	20.836(2)	30.944(2)
α/°	90	90	90
β/°	110.84(5)	95.34(1)	96.068(2)
γ/°	90	90	90
Volume/Å^3	7029(2)	3403(2)	5923.0(8)
Z	4	4	4
ρ_{calc}g / cm^3	1.775	1.964	1.472
μ / mm^{-1}	7.292	7.470	5.578
F(000)	3640.0	3640.0	2544.0
Crystal size / mm^3	0.18 × 0.13 × 0.05	0.28 × 0.16 × 0.16	0.42 × 0.33 × 0.04
Radiation	MoKα (λ = 0.71073)	MoKα (λ = 0.71073)	MoKα (λ = 0.71073)
2Θ range for data collection/°	6.684 to 49.998	5.07 to 49.998	4.502 to 48.996
Index ranges	-21 ≤ h ≤ 21, -20 ≤ k ≤ 21, -22 ≤ l ≤ 26	-27 ≤ h ≤ 27, -16 ≤ k ≤ 16, -44 ≤ l ≤ 24	-11 ≤ h ≤ 11, -22 ≤ k ≤ 21, -36 ≤ l ≤ 35
Reflections collected	48084	71535	61212
Independent reflections	12347 [R_{int} = 0.1768, R_{sigma} = 0.1770]	5621 [R_{int} = 0.0490, R_{sigma} = 0.0205]	9850 [R_{int} = 0.0415, R_{sigma} = 0.0274]
Data/restraints/parameters	12347/10/593	5621/23/375	9850/0/541
Goodness-of-fit on F^2	0.853	1.021	1.033
Final R indexes [I>=2σ (I)]	R_1 = 0.0596, wR_2 = 0.1140	R_1 = 0.0224, wR_2 = 0.0532	R_1 = 0.0293, wR_2 = 0.0780
Final R indexes [all data]	R_1 = 0.1392, wR_2 = 0.1348	R_1 = 0.0238, wR_2 = 0.0541	R_1 = 0.0318, wR_2 = 0.0793
Largest diff. peak/hole / e Å^{-3}	1.67/-1.53	1.58/-2.02	1.88/-1.30
Diffractometer	STOE IPDS	Bruker APEX	Bruker APEX
CCDC access code	2269018	2269019	2269020

Table S1: Crystallographic data and data collection parameters (continued)

	$[Ga\subset\{Au_2(L1^{ethyl})_2\}](BF_4)$ (**18b**)	$[(AuCl)_2(H_2L^{ethyl})]$ (**19**)	$[\{Au(PPh_3)\}_2(L1^{ethyl})]$ (**20**)
Empirical formula	$C_{34}H_{46}Au_2GaBF_4N_{10}O_4S_4$	$C_{17}H_{25}Au_2Cl2N_5O_2S_2$	$C_{53}H_{53}Au_2P_2N_5O_2S_2$
Formula weight	1337.51	860.37	1311.99
Temperature/K	293	100	200
Crystal system	Monoclinic	Monoclinic	Monoclinic
Space group	$P2_1$	$P2_1/c$	$P2_1/n$
a/Å	10.125(2)	7.840(1)	9.0940(6)
b/Å	19.214(4)	21.273(3)	21.766(1)
c/Å	14.846(3)	15.049(3)	13.1519(9)
α/°	90	90	90
β/°	97.23(3)	99.02(1)	104.760(5)
γ/°	90	90	90
Volume/Å^3	2865(1)	2478.8(7)	2517.4(3)
Z	2	4	2
ρ_{calc}g / cm^3	1.550	2.305	1.731
μ / mm^{-1}	5.772	12.231	6.013
F(000)	1292.0	1608.0	1284.0
Crystal size / mm^3	0.23 × 0.11 × 0.02	0.18 × 0.06 × 0.05	0.26 × 0.18 × 0.18
Radiation	MoKα (λ = 0.71073)	MoKα (λ = 0.71073)	MoKα (λ = 0.71073)
2Θ range for data collection/°	3.484 to 51.998	4.71 to 57.314	6.676 to 51.998
Index ranges	-12 ≤ h ≤ 12, -23 ≤ k ≤ 21, -18 ≤ l ≤ 18	-10 ≤ h ≤ 9, -28 ≤ k ≤ 28, -20 ≤ l ≤ 20	-11 ≤ h ≤ 11, -26 ≤ k ≤ 26, -16 ≤ l ≤ 12
Reflections collected	25921	85691	14876
Independent reflections	10417 [R_{int} = 0.1144, R_{sigma} = 0.1520]	6281 [R_{int} = 0.0886, R_{sigma} = 0.0436]	4927 [R_{int} = 0.0338, R_{sigma} = 0.0289]
Data/restraints/parameters	10417/14/541	6281/0/274	4927/340/344
Goodness-of-fit on F^2	0.816	1.043	1.321
Final R indexes [I>=2σ (I)]	R_1 = 0.0517, wR_2 = 0.0981	R_1 = 0.0318, wR_2 = 0.0348	R_1 = 0.0679, wR_2 = 0.1473
Final R indexes [all data]	R_1 = 0.1012, wR_2 = 0.1130	R_1 = 0.0603, wR_2 = 0.0381	R_1 = 0.0751, wR_2 = 0.1497
Largest diff. peak/hole / e Å^{-3}	0.57/-1.19	0.84/-1.20	2.52/-1.87
Diffractometer	STOE IPDS	Bruker APEX	STOE IPDS
CCDC access code	2269021	2269022	2269023

Table S1: Crystallographic data and data collection parameters (continued)

	[Zn⊂{$Au_2(L1^{ethyl})_2$}] (**21**)	[Sc(H_2O)$_2${Au($L1^{ethyl}$)$_2$}] (**22**)
Empirical formula	$C_{34}H_{46}Au_2ZnN_{10}O_4S_4$	$C_{34}H_{49}AuScN_{10}O_6S_4$
Formula weight	1246.35	1064.00
Temperature/K	100	104
Crystal system	Monoclinic	Triclinicl
Space group	$P2_1/c$	P-1
a/Å	15.2417(4)	14.328(1)
b/Å	22.4929(6)	19.061(2)
c/Å	14.3165(4)	20.604(2)
α/°	90	73.209(2)
β/°	101.096(1)	88.754(2)
γ/°	90	87.069(2)
Volume/$Å^3$	4816.4(2)	5379.9(7)
Z	4	4
ρ_{calc}g / cm^3	1.719	1.314
μ / mm^{-1}	6.788	3.048
F(000)	2416.0	2140.0
Crystal size / mm^3	0.32 × 0.30 × 0.25	0.16 × 0.12 × 0.02
Radiation	MoKα (λ = 0.71073)	MoKα (λ = 0.71073)
2Θ range for data collection/°	4.532 to 52.858	4.532 to 50.50
Index ranges	-18 ≤ h ≤ 19, -28 ≤ k ≤ 28, -17 ≤ l ≤ 17	-17 ≤ h ≤ 17, -22 ≤ k ≤ 22, -24 ≤ l ≤ 24
Reflections collected	51459	96730
Independent reflections	9864 [R_{int} = 0.0422, R_{sigma} = 0.0294]	19445 [R_{int} = 0.0733, R_{sigma} = 0.0662]
Data/restraints/parameters	9864/0/497	19445/81/988
Goodness-of-fit on F^2	1.026	1.035
Final R indexes [I>=2σ (I)]	R_1 = 0.0251, wR_2 = 0.0469	R_1 = 0.0733, wR_2 = 0.1373
Final R indexes [all data]	R_1 = 0.0344, wR_2 = 0.0493	R_1 = 0.1095, wR_2 = 0.1543
Largest diff. peak/hole / e $Å^{-3}$	1.70/-1.36	3.05/-1.77
Diffractometer	Bruker APEX	Bruker APEX
CCDC access code	2269024	2269025

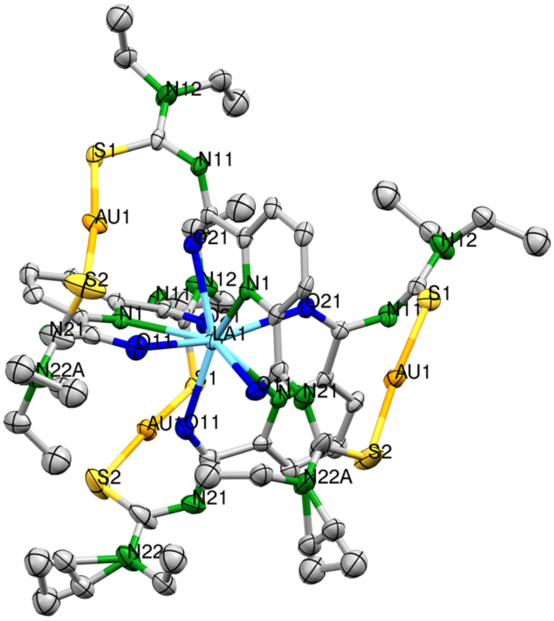

Figure S1. Ellipsoid representation of [La$\subset${$Au_3(L1^{ethyl})_3$}] (**1**) including the positional disorder in the peripheral ethyl groups. The thermal ellipsoids are set at a 50% probability level. Hydrogen atoms are omitted for clarity. A solvent mask was calculated and 53 electrons were found in a volume of 218 A^3 in 3 voids per unit cell. This is consistent with the presence of 2.5 H_2O per formula unit which account for 50 electrons per unit cell.

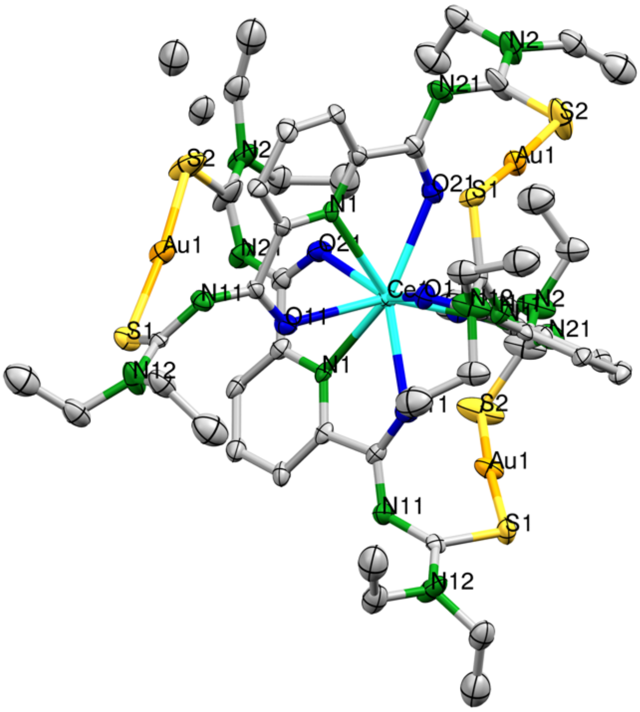

Figure S2. Ellipsoid representation of [Ce$\subset${$Au_3(L1^{ethyl})_3$}] (**2**) including the positional disorder in the peripheral ethyl groups. The thermal ellipsoids are set at a 50% probability level. Hydrogen atoms are omitted for clarity.

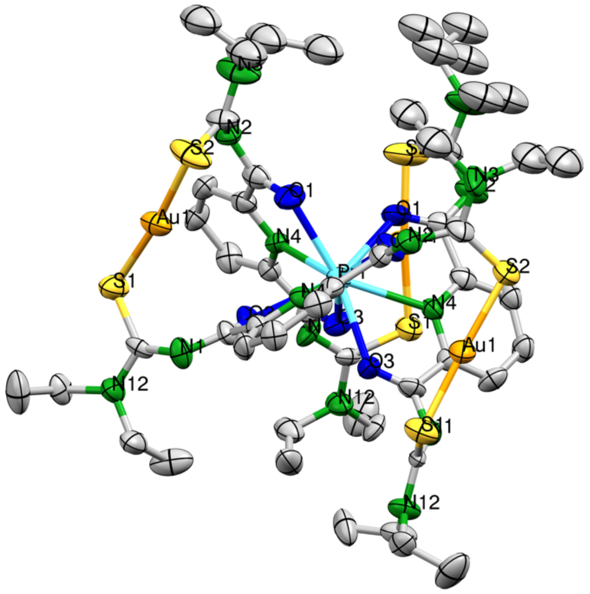

Figure S3: Ellipsoid representation of $[Pr\subset\{Au_3(L1^{ethyl})_3\}]$ (**3**) including the positional disorder in the peripheral ethyl groups. The thermal ellipsoids are set at a 50% probability level. Hydrogen atoms are omitted for clarity.

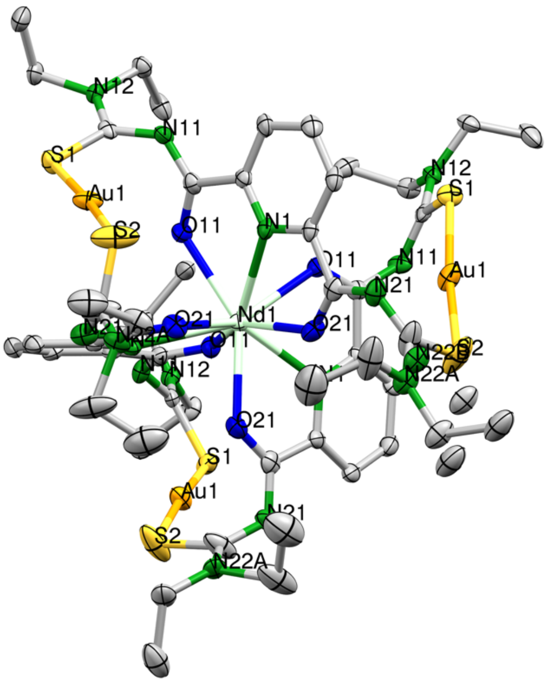

Figure S4: Ellipsoid representation of $[Nd\subset\{Au_3(L1^{ethyl})_3\}]$ (**4**) including the positional disorder in the peripheral ethyl groups. The thermal ellipsoids are set at a 50% probability level. Hydrogen atoms are omitted for clarity. A solvent mask was calculated and 54 electrons were found in a volume of 247 A^3 in 4 voids per unit cell. This is consistent with the presence of 2.5 H_2O per formula unit which account for 50 electrons per unit cell.

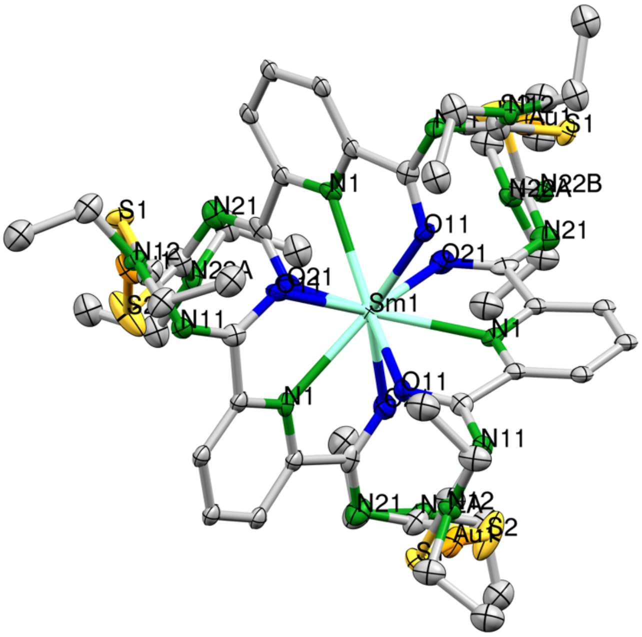

Figure S5: Ellipsoid representation of $[Sm\subset\{Au_3(L1^{ethyl})_3\}]$ (**5**). The thermal ellipsoids are set at a 50% probability level. Hydrogen atoms are omitted for clarity. A solvent mask was calculated and 55 electrons were found in a volume of 240 A^3 in 4 voids per unit cell. This is consistent with the presence of 2.5 H_2O per formula unit which account for 50 electrons per unit cell.

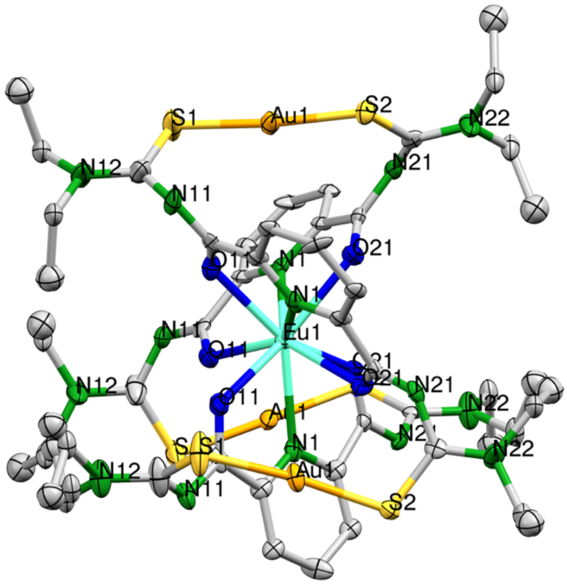

Figure S6: Ellipsoid representation of $[Eu\subset\{Au_3(L1^{ethyl})_3\}]$ (**6**). The thermal ellipsoids are set at a 50% probability level. Hydrogen atoms are omitted for clarity. A solvent mask was calculated and 46 electrons were found in a volume of 227 A^3 in 3 voids per unit cell. This is consistent with the presence of 2.5 H_2O per formula unit which account for 50 electrons per unit cell.

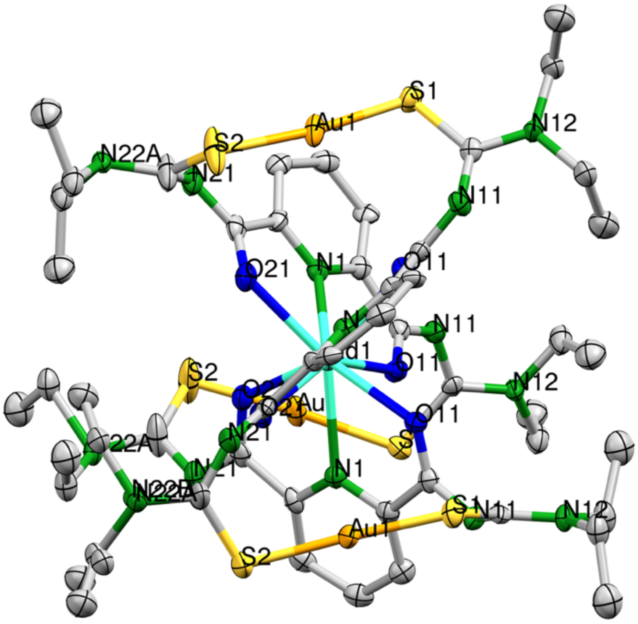

Figure S7: Ellipsoid representation of $[Gd\subset\{Au_3(L1^{ethyl})_3\}]$ (**7**). The thermal ellipsoids are set at a 50% probability level. Hydrogen atoms are omitted for clarity. A solvent mask was calculated and 65 electrons were found in a volume of 179 A^3 in 2 voids per unit cell. This is consistent with the presence of 3.5 H_2O per formula unit which account for 70 electrons per unit cell.

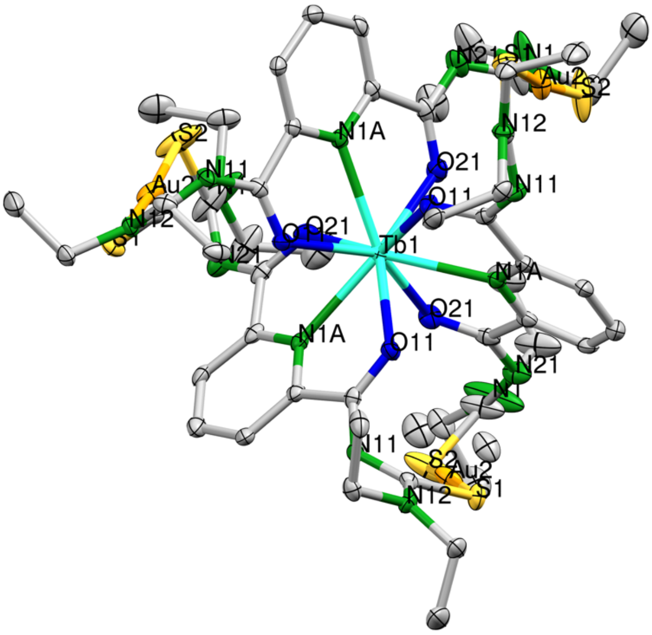

Figure S8: Ellipsoid representation of $[Tb\subset\{Au_3(L1^{ethyl})_3\}]$ (**8**) including the positional disorder in the peripheral ethyl groups. The thermal ellipsoids are set at a 50% probability level. Hydrogen atoms are omitted for clarity. A solvent mask was calculated and 48 electrons were found in a volume of 252 A^3 in 3 voids per unit cell. This is consistent with the presence of 2.5 H_2O per formula unit which account for 50 electrons per unit cell.

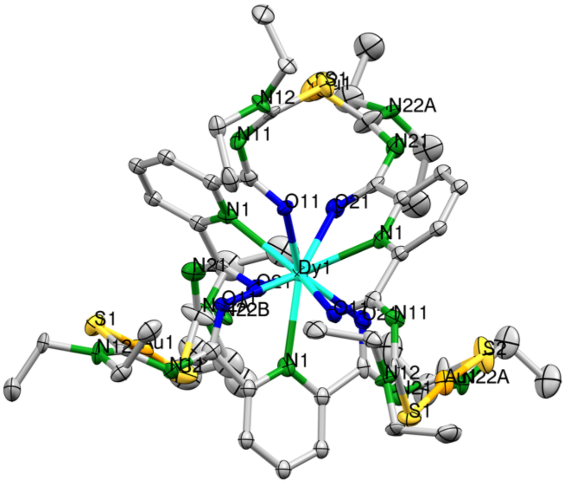

Figure S9: Ellipsoid representation of [Dy⊂{Au_3($L1^{ethyl}$)$_3$}] (**9**). The thermal ellipsoids are set at a 50% probability level. Hydrogen atoms are omitted for clarity. A solvent mask was calculated and 43 electrons were found in a volume of 138 A^3 in 1 void per unit cell. This is consistent with the presence of 2 H_2O per formula unit which account for 40 electrons per unit cell.

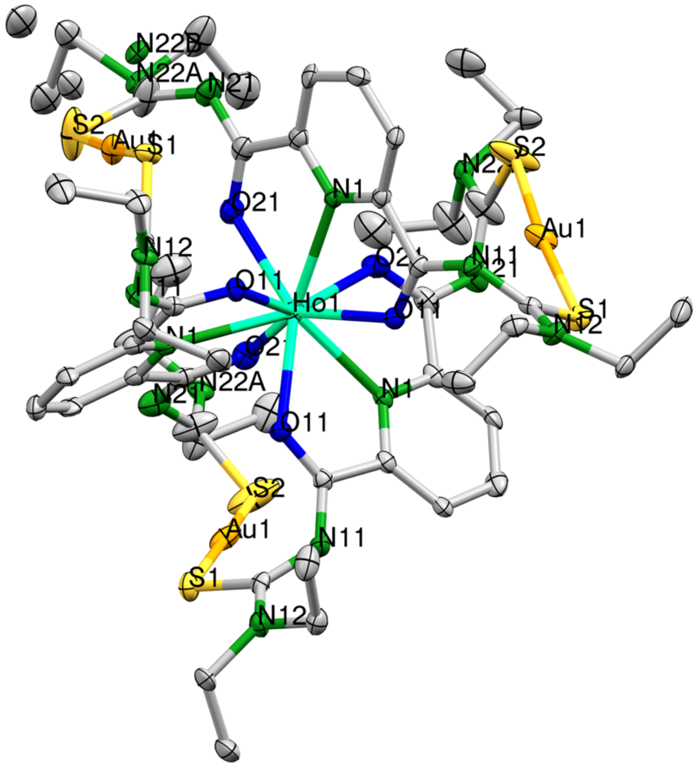

Figure S10: Ellipsoid representation of [Ho⊂{Au_3($L1^{ethyl}$)$_3$}] (**10**) including the positional disorder in the peripheral ethyl groups. The thermal ellipsoids are set at a 50% probability level. Hydrogen atoms are omitted for clarity. A solvent mask was calculated and 43 electrons were found in a volume of 282 A^3 in 3 voids per unit cell. This is consistent with the presence of 2 H_2O per formula unit which account for 40 electrons per unit cell.

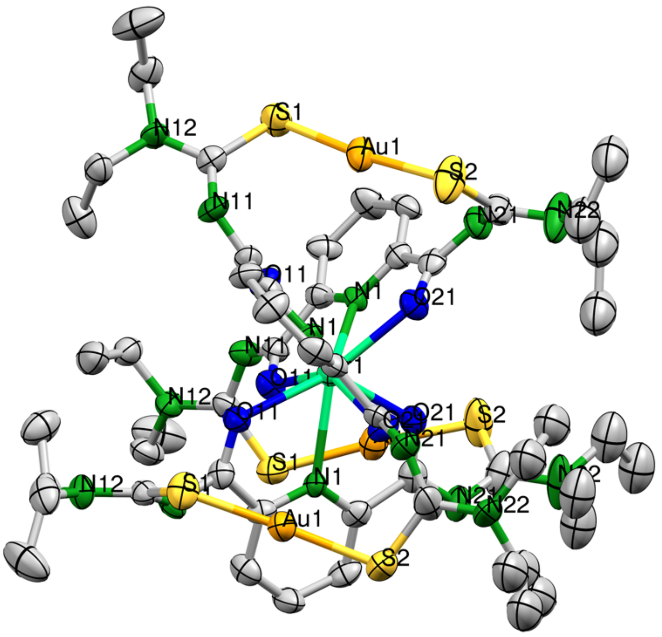

Figure S11: Ellipsoid representation of [Er⊂{Au_3(L1^{ethyl})$_3$}] (**11**) including the positional disorder in the peripheral N(ethyl)$_2$ groups. The thermal ellipsoids are set at a 50% probability level. Hydrogen atoms are omitted for clarity.

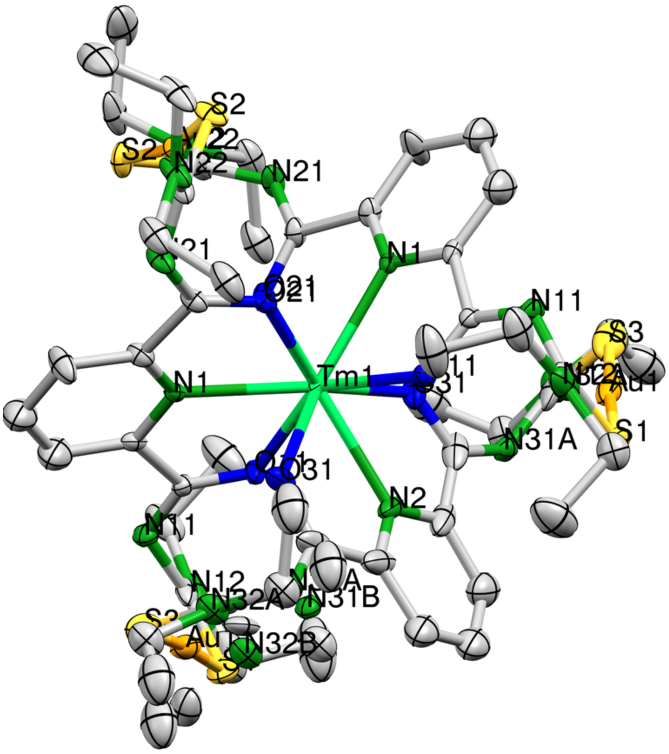

Figure S12: Ellipsoid representation of [Tm⊂{Au_3(L1^{ethyl})$_3$}] (**12**) including the positional disorder in the peripheral N(ethyl)$_2$ groups. The thermal ellipsoids are set at a 50% probability level. Hydrogen atoms are omitted for clarity.

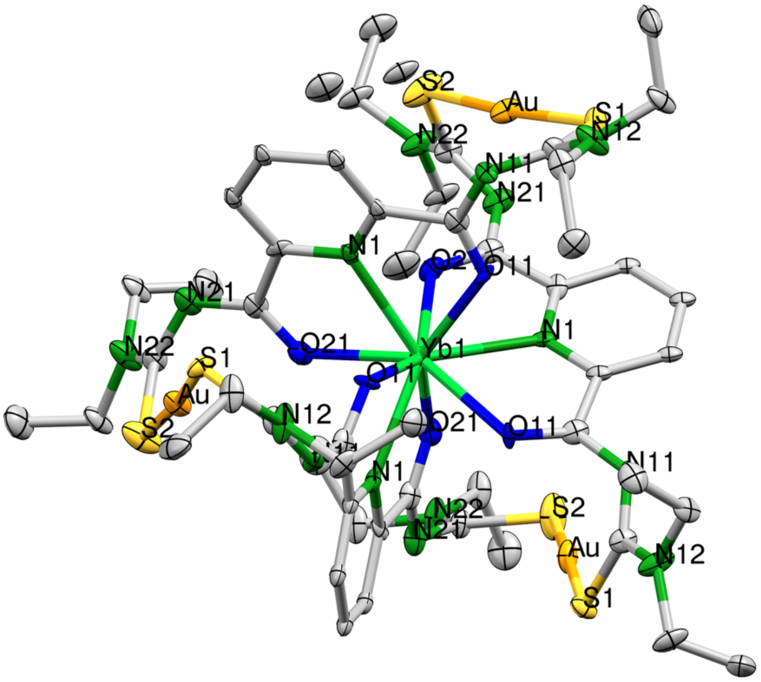

Figure S13: Ellipsoid representation of $[Yb\subset\{Au_3(L1^{ethyl})_3\}]$ (**13**) including the positional disorder in the peripheral ethyl groups. The thermal ellipsoids are set at a 50% probability level. Hydrogen atoms are omitted for clarity. A solvent mask was calculated and 33 electrons were found in a volume of 235 A^3 in 3 voids per unit cell. This is consistent with the presence of 1.5 H_2O per formula unit which account for 30 electrons per unit cell.

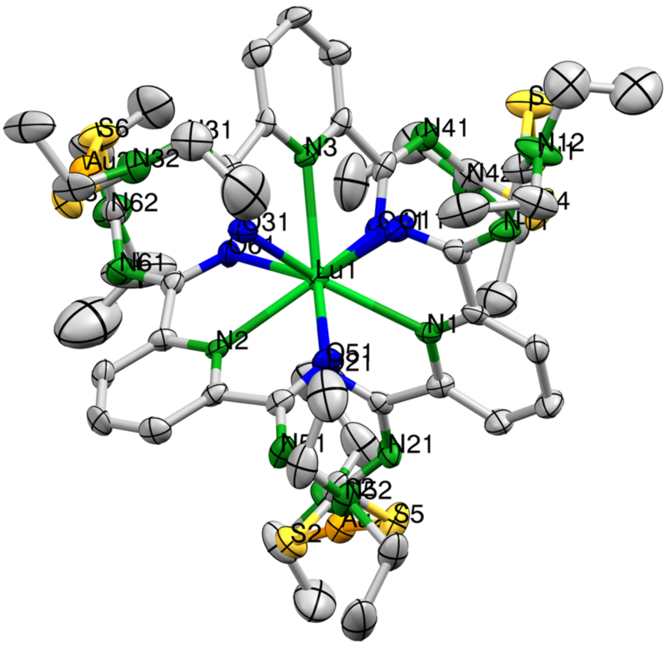

Figure S14: Ellipsoid representation of $[Lu\subset\{Au_3(L1^{ethyl})_3\}]$ (**14**). The thermal ellipsoids are set at a 50% probability level. Hydrogen atoms are omitted for clarity.

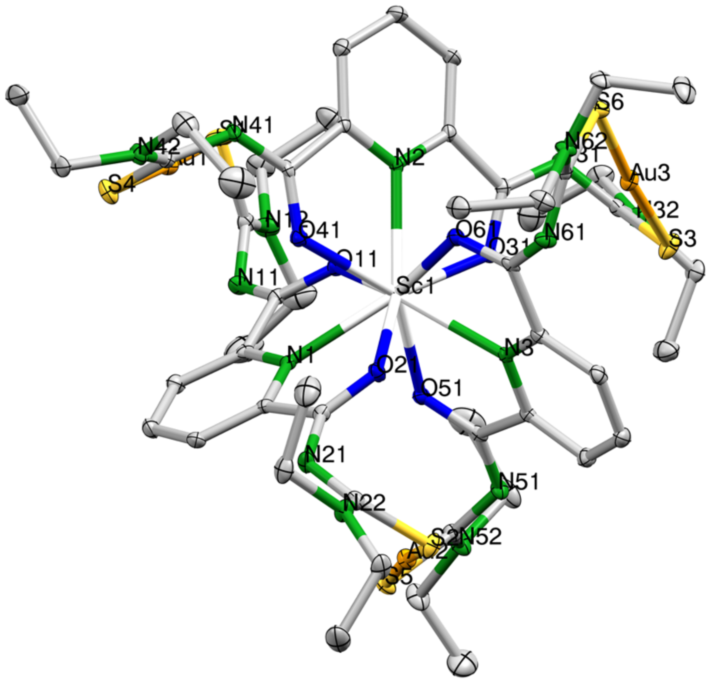

Figure S15: Ellipsoid representation of $[Sc\subset\{Au_3(L1^{ethyl})_3\}]$ (**15**) including the positional disorder in the peripheral ethyl groups. The thermal ellipsoids are set at a 50% probability level. Hydrogen atoms are omitted for clarity.

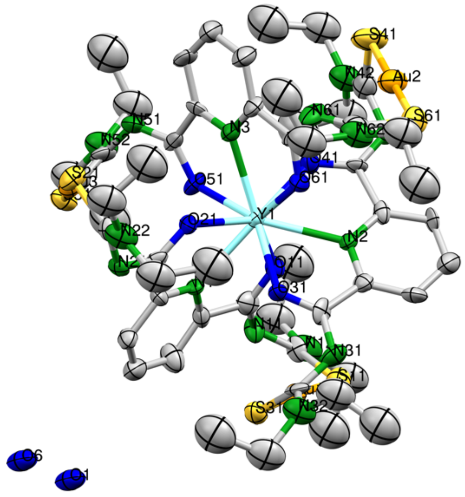

Figure S16: Ellipsoid representation of $[Y\subset\{Au_3(L1^{ethyl})_3\}]$ (**16**) x H_2O. The thermal ellipsoids are set at a 50% probability level. Hydrogen atoms are omitted for clarity.

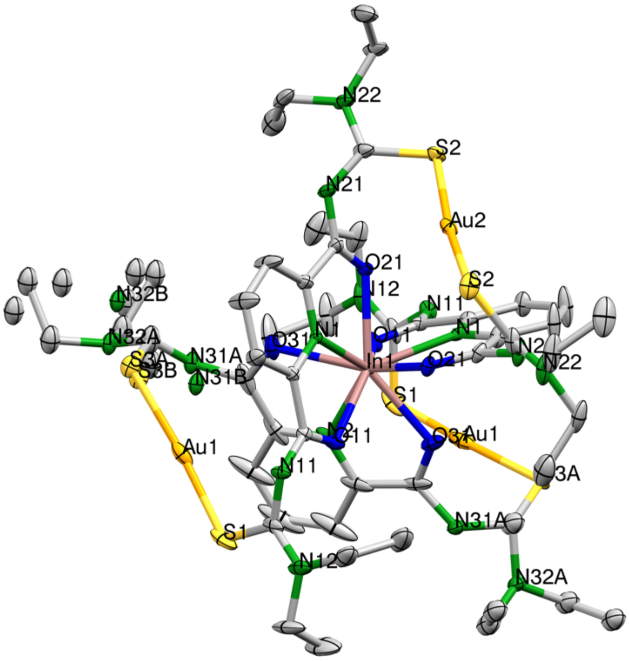

Figure S17: Ellipsoid representation of [In⊂{$Au_3(L1^{ethyl})_3$}] (**17**) including the positional disorder in the peripheral ethyl groups. The thermal ellipsoids are set at a 50% probability level. Hydrogen atoms are omitted for clarity.

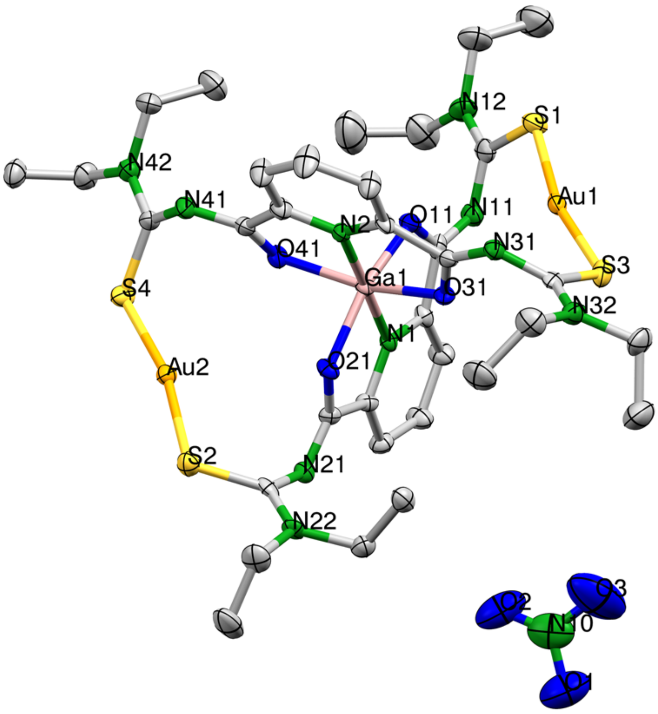

Figure S18: Ellipsoid representation of [Ga⊂{$Au_2(L1^{ethyl})_2$}](NO_3) (**18a**). The thermal ellipsoids are set at a 50% probability level. Hydrogen atoms are omitted for clarity. A solvent mask was calculated and 334 electrons were found in a volume of 2100 A^3 in 1 void per unit cell. This is consistent with the presence of 2 CH_2Cl_2 per formula unit which account for 336 electrons per unit cell.

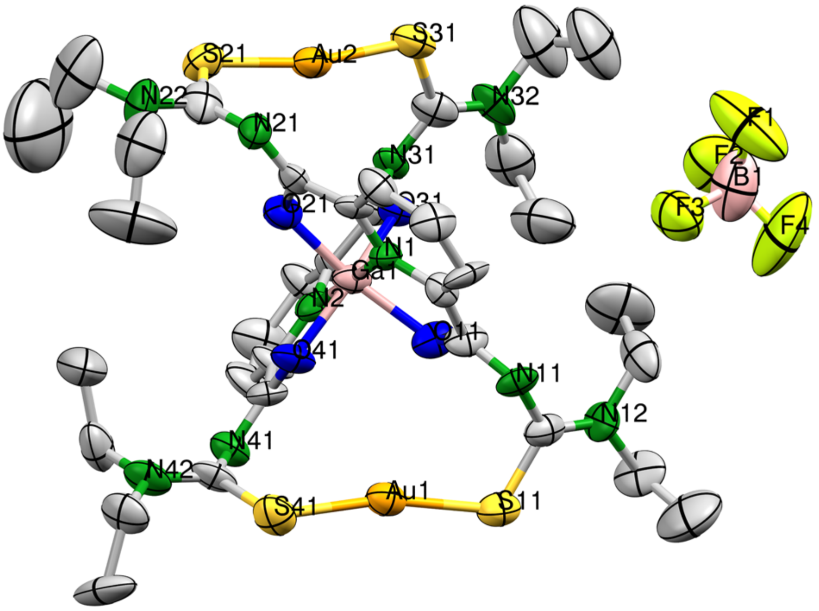

Figure S19: Ellipsoid representation of $[Ga\subset\{Au_2(L1^{ethyl})_2\}](BF_4)$ (**18b**). The thermal ellipsoids are set at a 50% probability level. Hydrogen atoms are omitted for clarity.

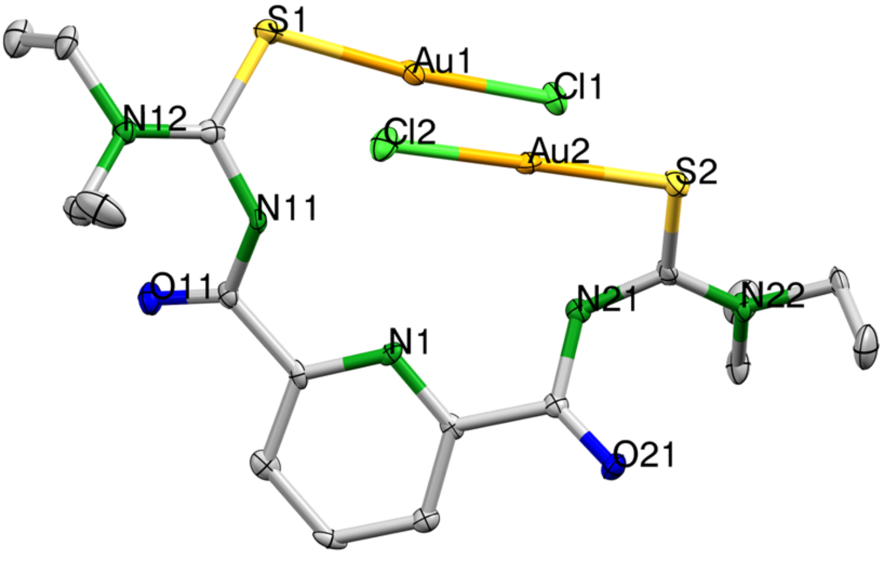

Figure S20: Ellipsoid representation of $[(AuCl)_2(H_2L1^{ethyl})]$ (**19**). The thermal ellipsoids are set at a 50% probability level. Hydrogen atoms are omitted for clarity.

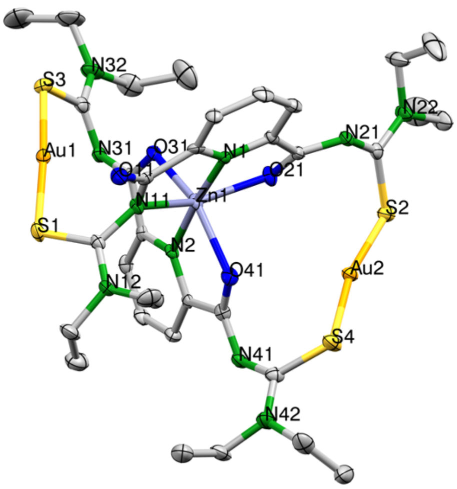

Figure S21: Ellipsoid representation of $[Zn\subset\{Au_2(L1^{ethyl})_2\}]$ (**21**). The thermal ellipsoids are set at a 50% probability level. Hydrogen atoms are omitted for clarity. A solvent mask was calculated and 220 electrons were found in a volume of 946 A^3 in 1 void per unit cell. This is consistent with the presence of 6 H_2O per formula unit which account for 240 electrons per unit cell.

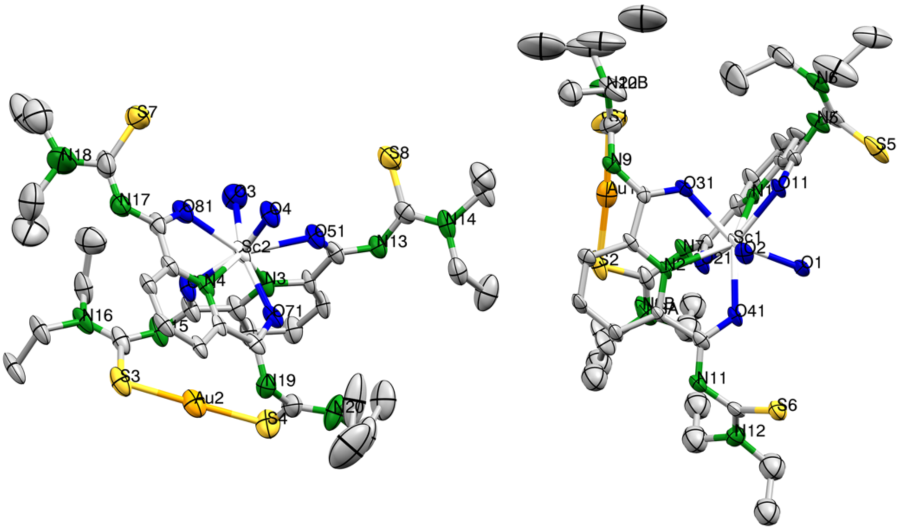

Figure S22: Ellipsoid representation of $[Sc(H_2O)_2\{Au(L1^{ethyl})_2\}]$ (**22**) including the positional disorder in the peripheral ethyl groups. The thermal ellipsoids are set at a 50% probability level. Hydrogen atoms are omitted for clarity.

Spectroscopic data

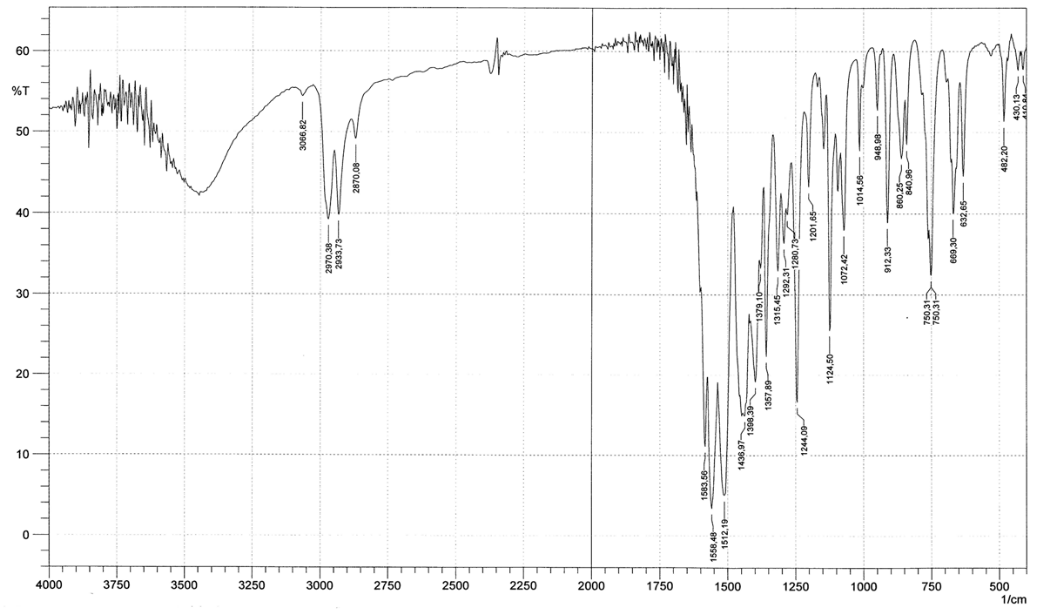

Figure S23: IR (KBr) spectrum of [La⊂{Au_3(L1^{ethyl})$_3$}] (**1**).

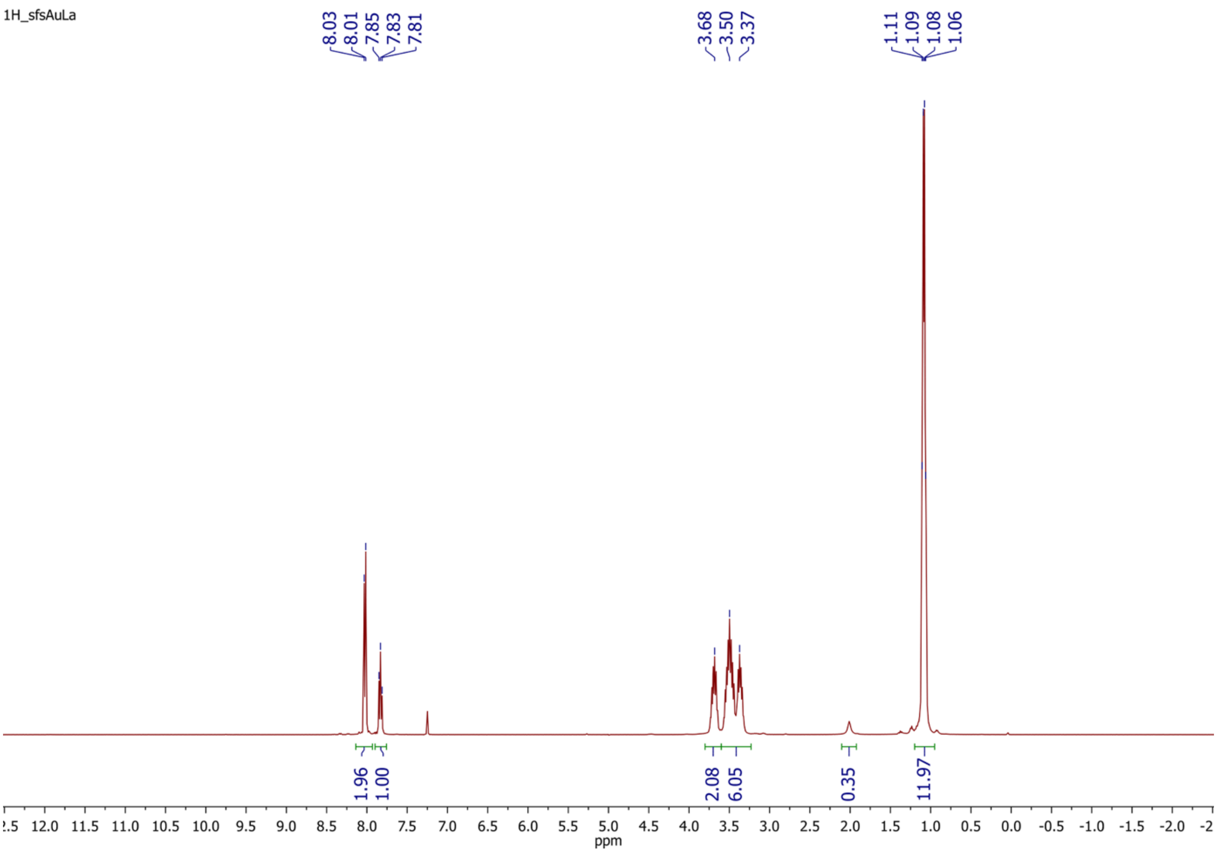

Figure S24: ^{1}H NMR spectrum of [La⊂{Au_3(L1^{ethyl})$_3$}] (**1**) in $CDCl_3$.

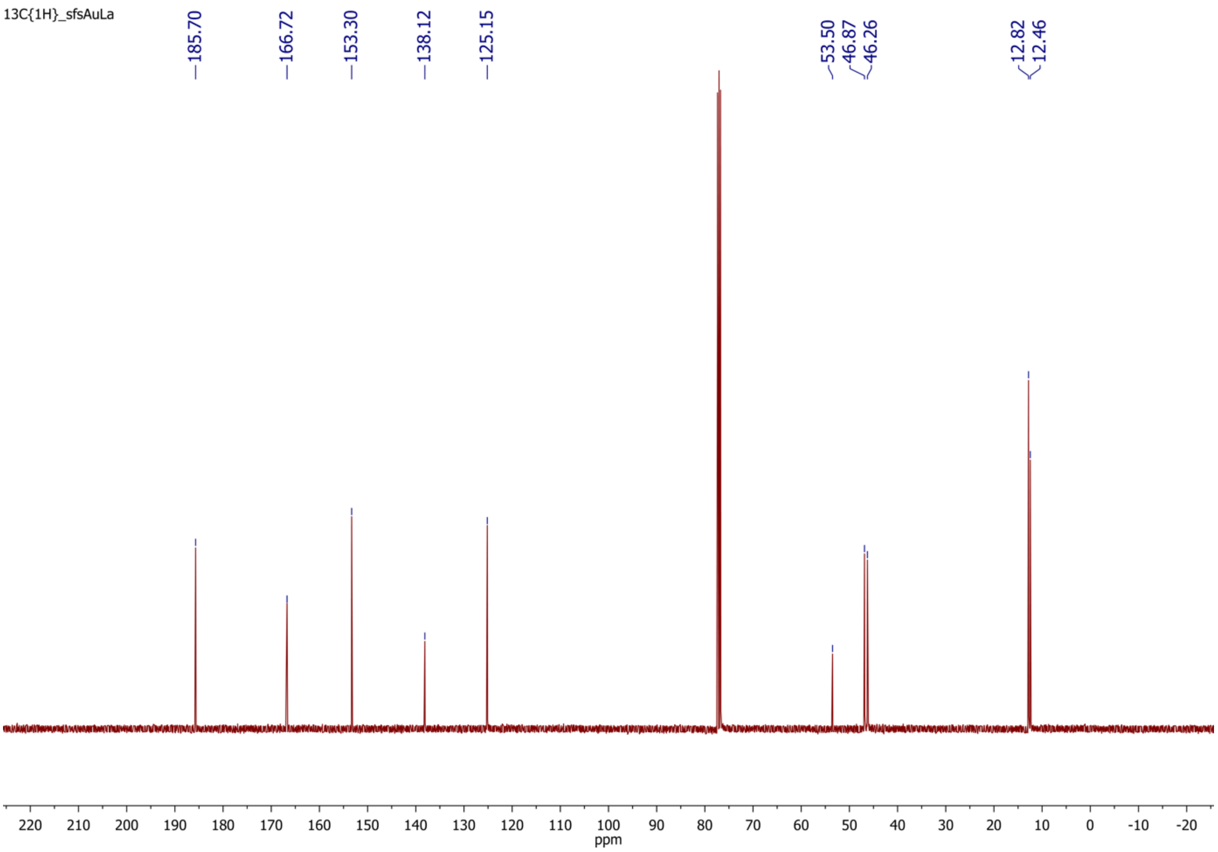

Figure S25: ^{13}C NMR spectrum of [La⊂{Au_3(L1^{ethyl})$_3$}] (**1**) in $CDCl_3$.

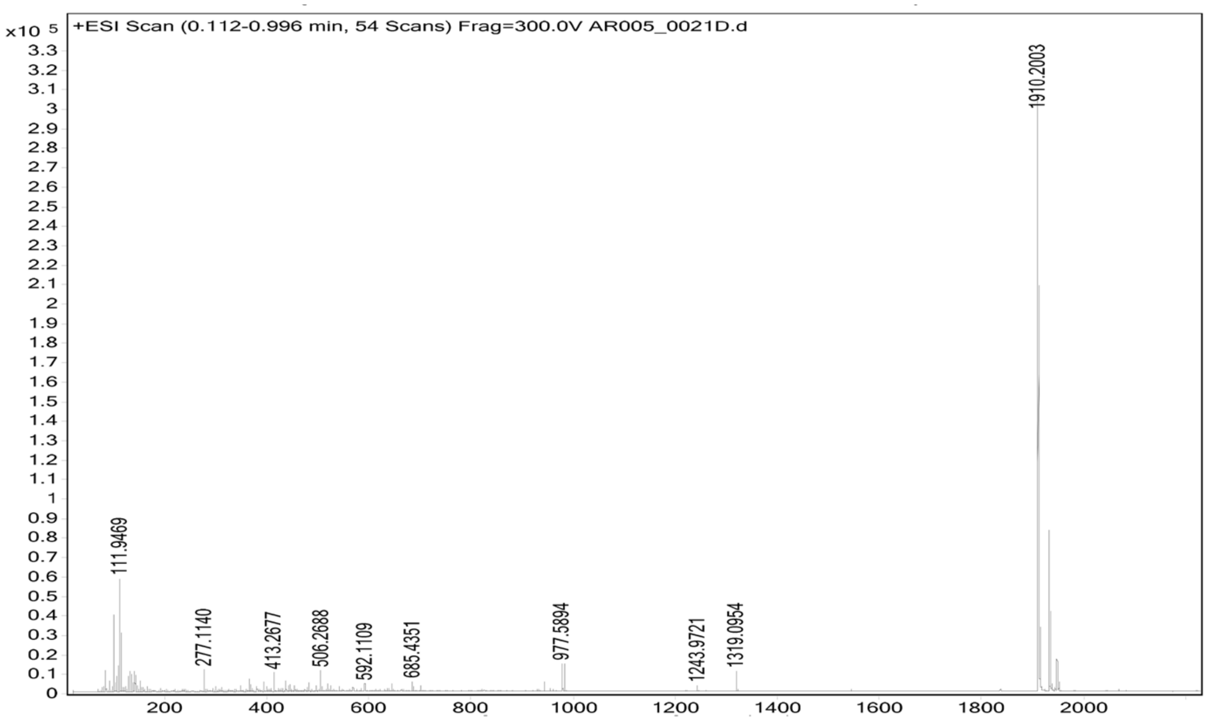

Figure S26: ESI+ MS spectrum of [La⊂{Au_3(L1^{ethyl})$_3$}] (**1**).

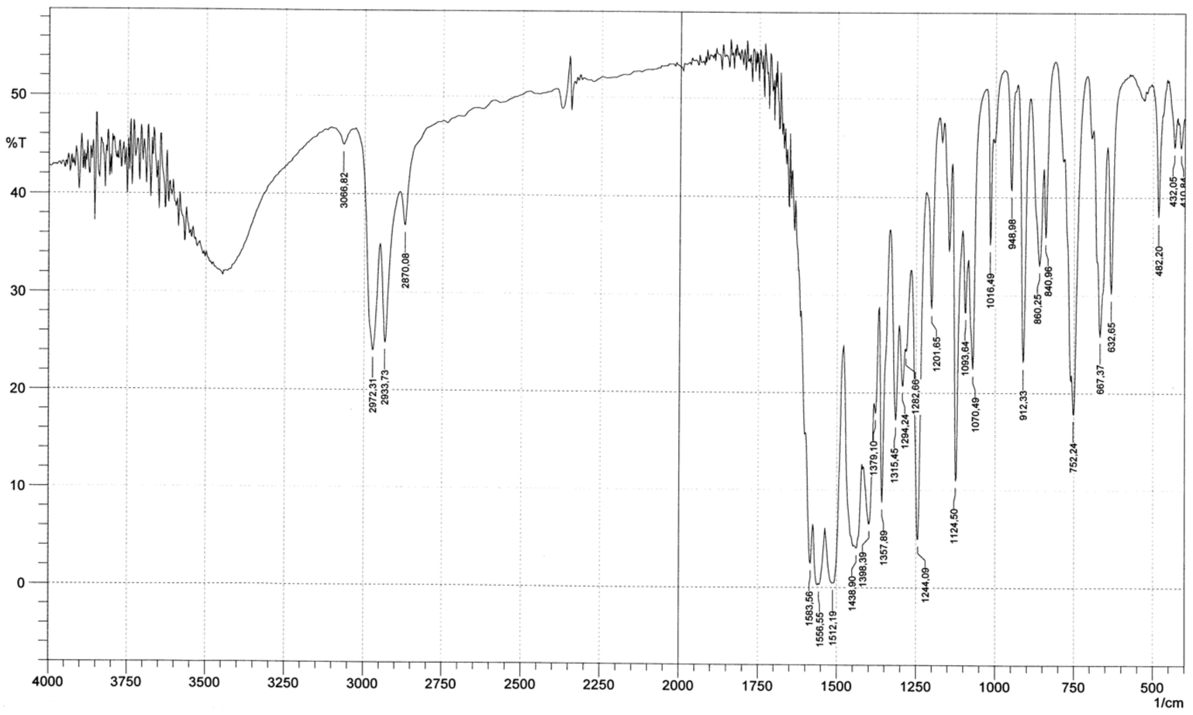

Figure S27: IR (KBr) spectrum of [Ce⊂{Au_3($L1^{ethyl}$)$_3$}] (**2**).

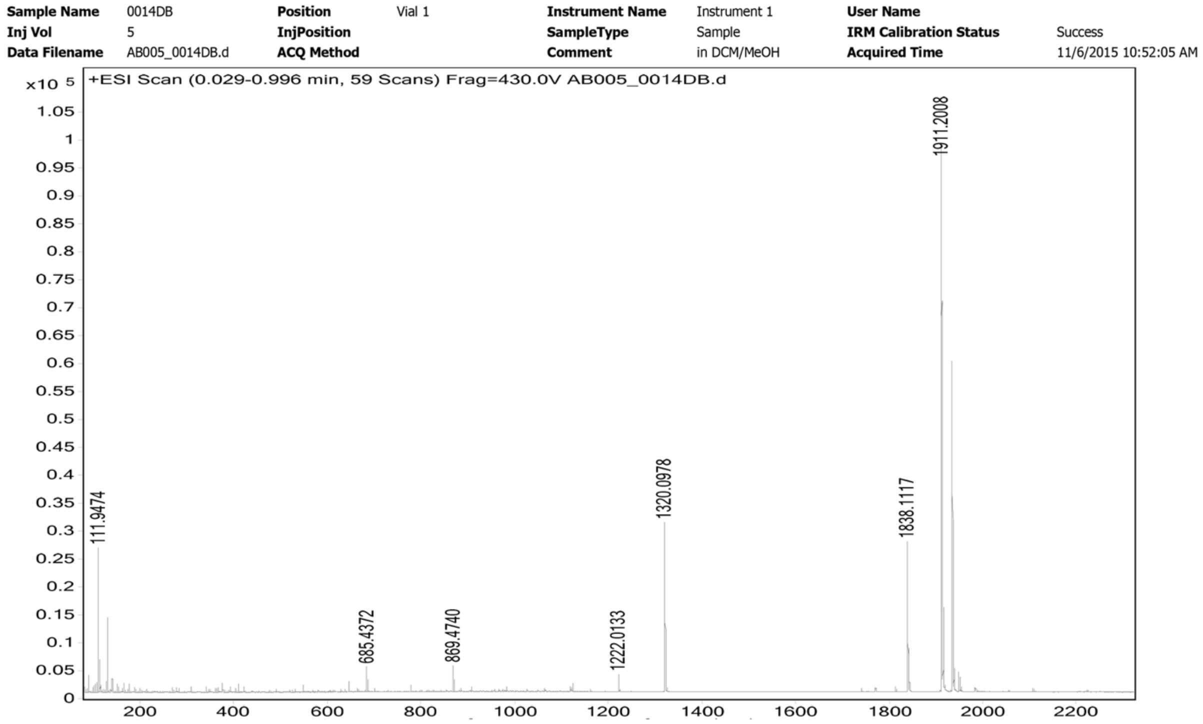

Figure S28: ESI+ MS spectrum of [Ce⊂{Au_3($L1^{ethyl}$)$_3$}] (**2**).

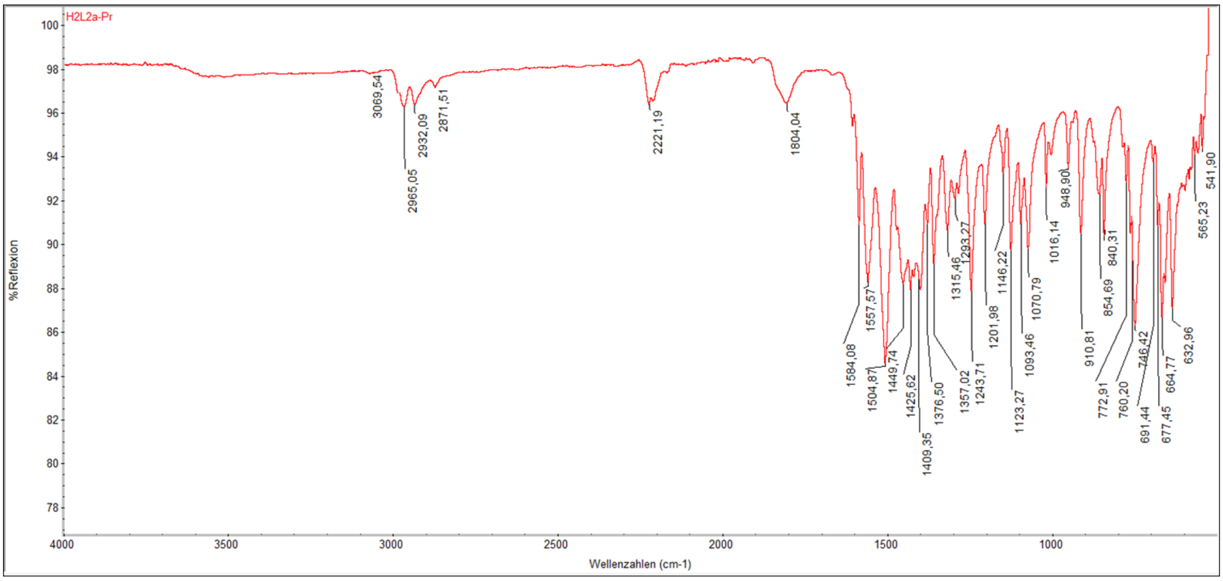

Figure S29: IR (ATR) spectrum of [Pr⊂{$Au_3(L1^{ethyl})_3$}] (**3**).

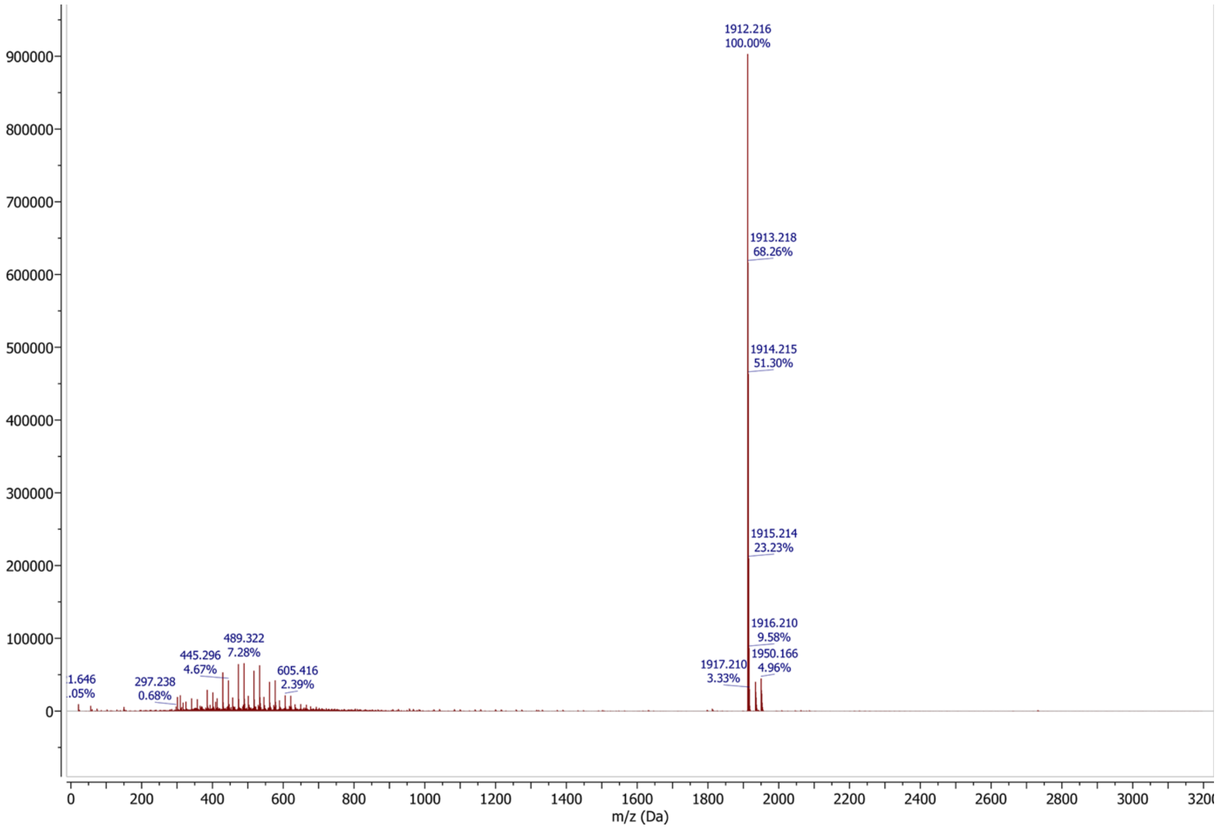

Figure S30: ESI+ MS spectrum of [Pr⊂{$Au_3(L1^{ethyl})_3$}] (**3**).

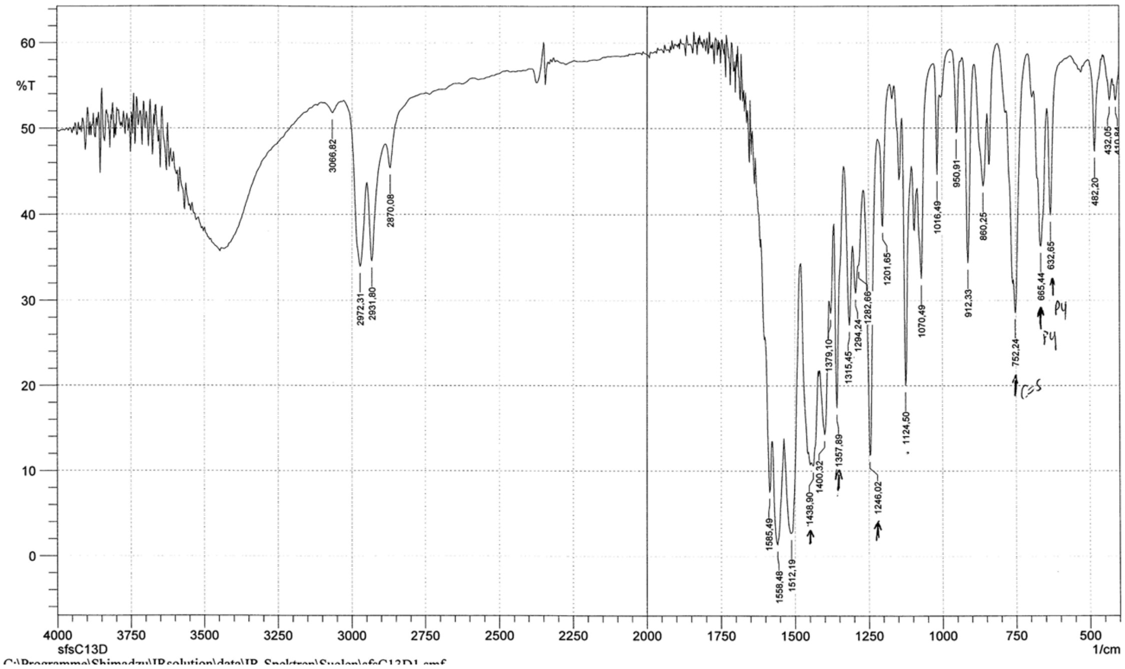

Figure S31: IR (KBr) spectrum of [Nd⊂{Au_3(L1^{ethyl})$_3$}] (**4**).

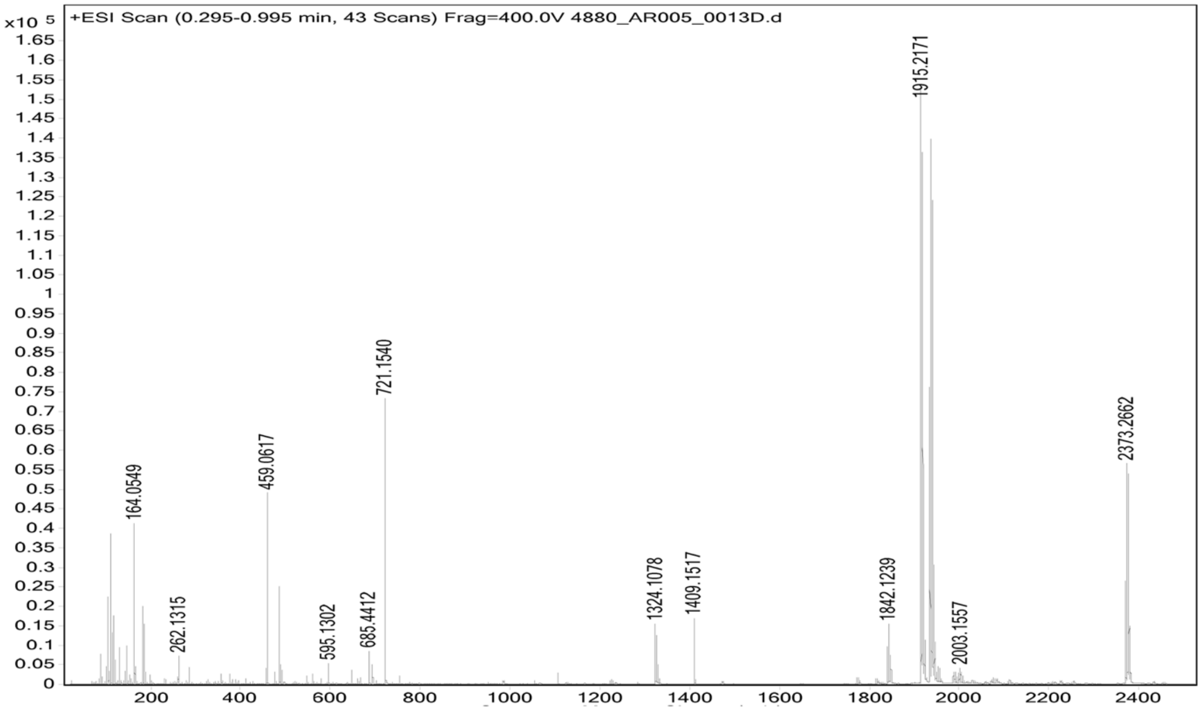

Figure S32: ESI+ MS spectrum of [Nd⊂{Au_3(L1^{ethyl})$_3$}] (**4**).

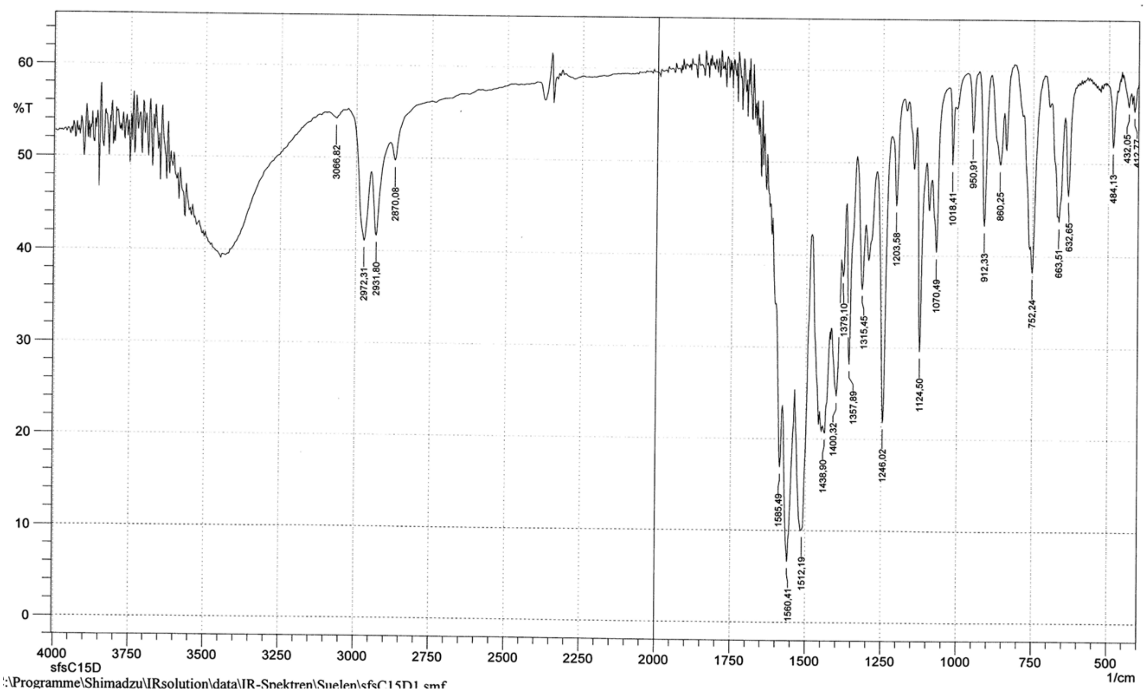

Figure S33: IR (KBr) spectrum of [Sm⊂{Au$_3$(L1^{ethyl})$_3$}] (**5**).

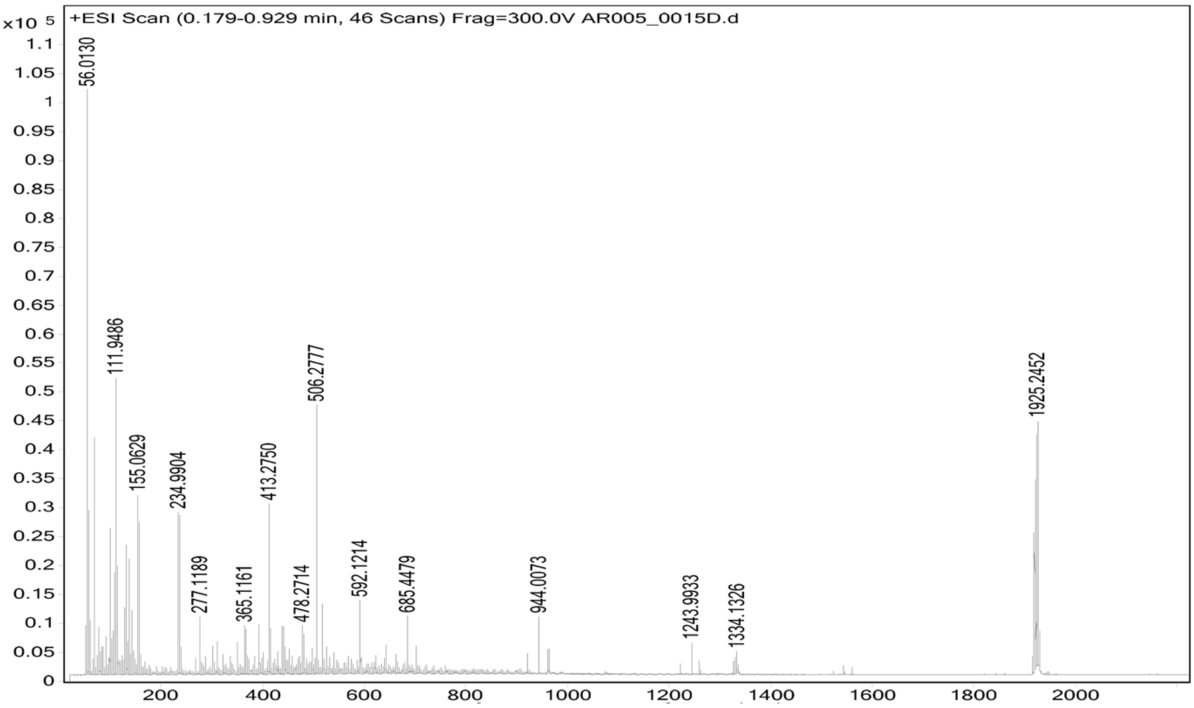

Figure S34: ESI+ MS spectrum of [Sm⊂{Au$_3$(L1^{ethyl})$_3$}] (**5**).

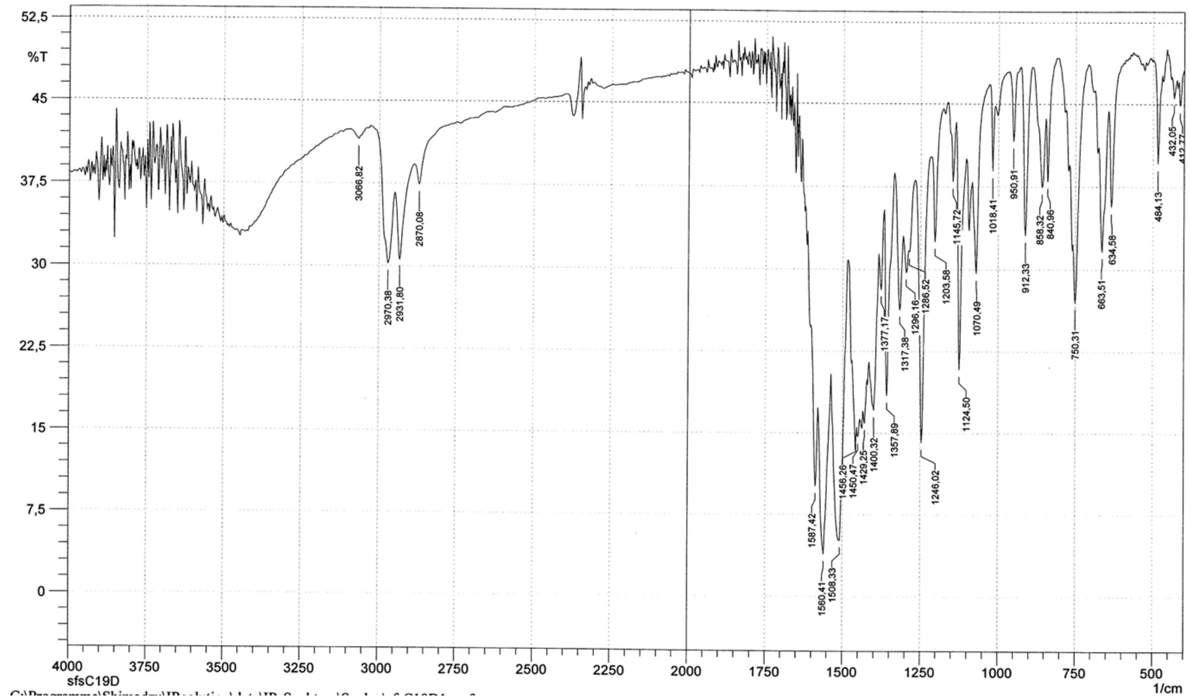

Figure S35: IR (KBr) spectrum of [Eu⊂{$Au_3(L1^{ethyl})_3$}] (**6**).

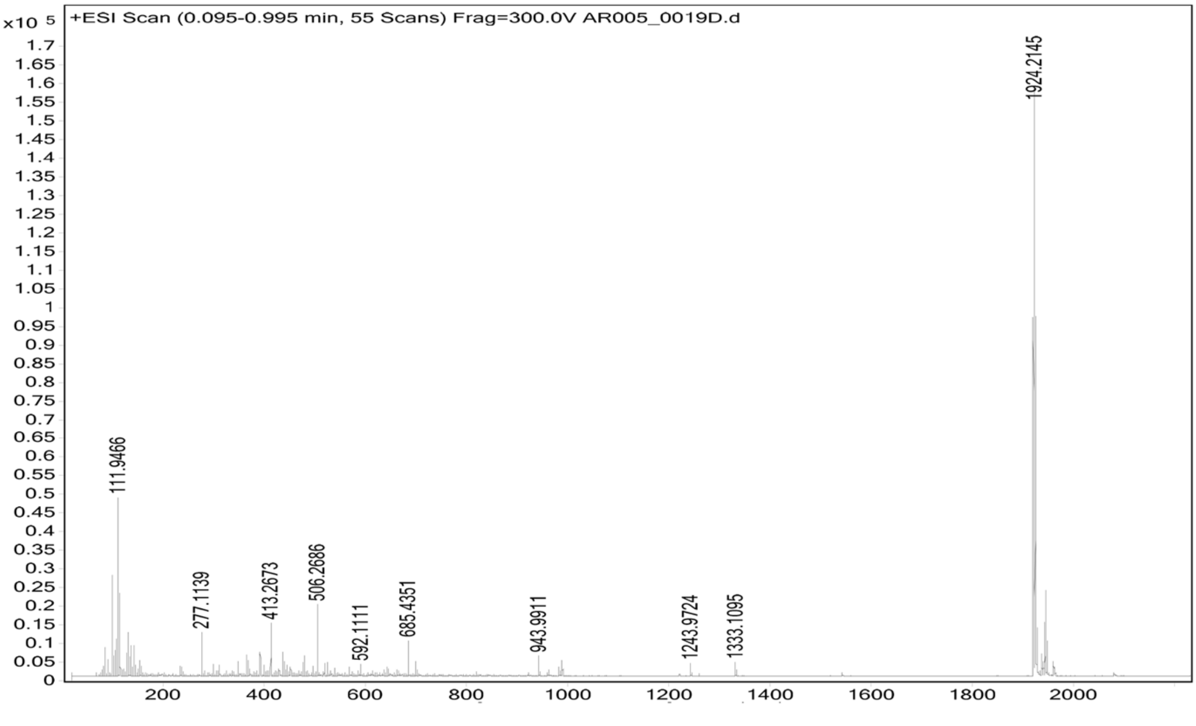

Figure S36: ESI+ MS spectrum of [Eu⊂{$Au_3(L1^{ethyl})_3$}] (**6**).

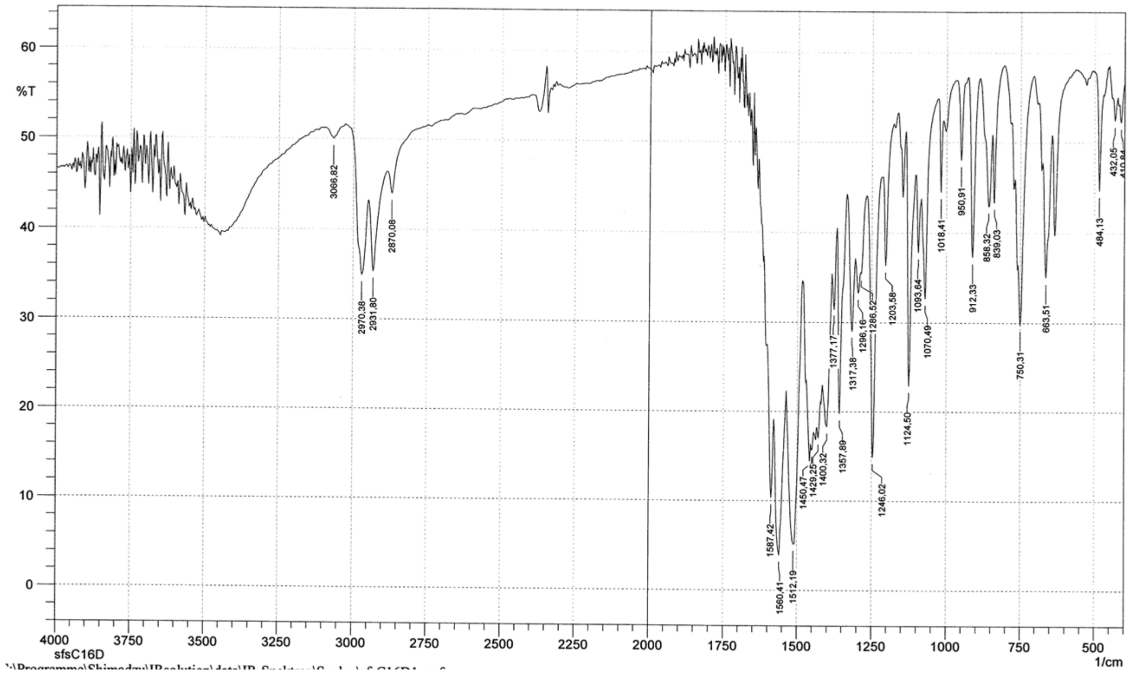

Figure S37: IR (KBr) spectrum of [Gd⊂{$Au_3(L1^{ethyl})_3$}] (**7**).

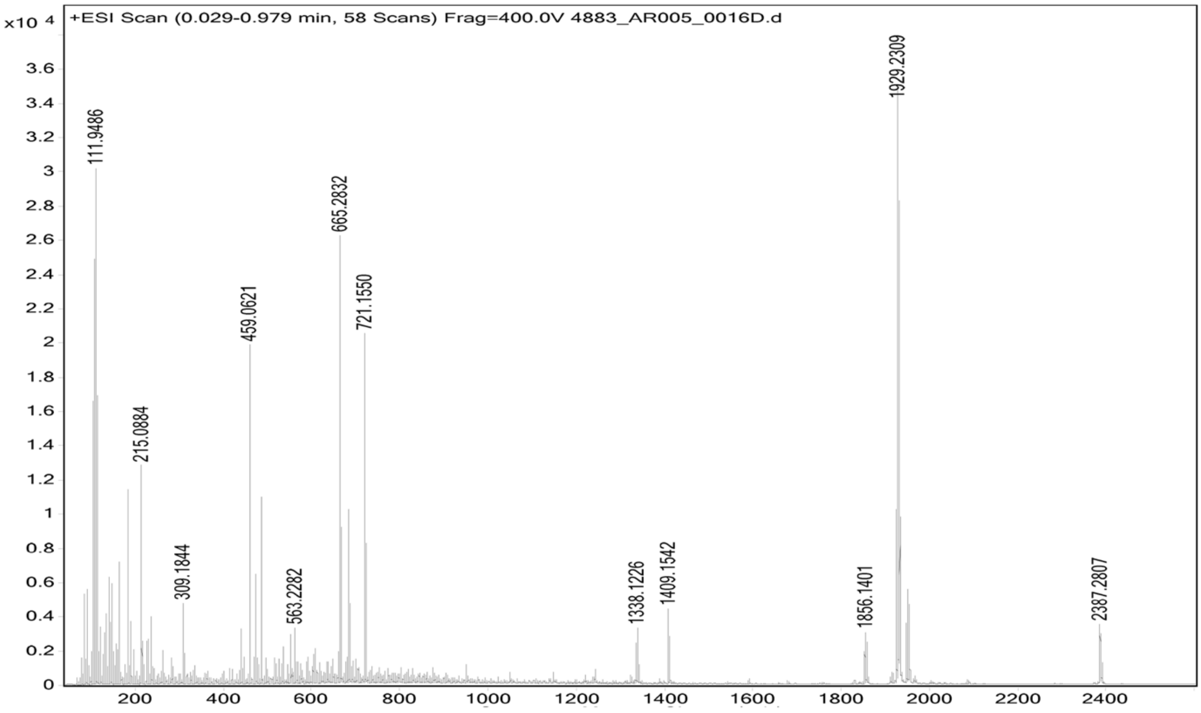

Figure S38: ESI+ MS spectrum of [Gd⊂{$Au_3(L1^{ethyl})_3$}] (**7**).

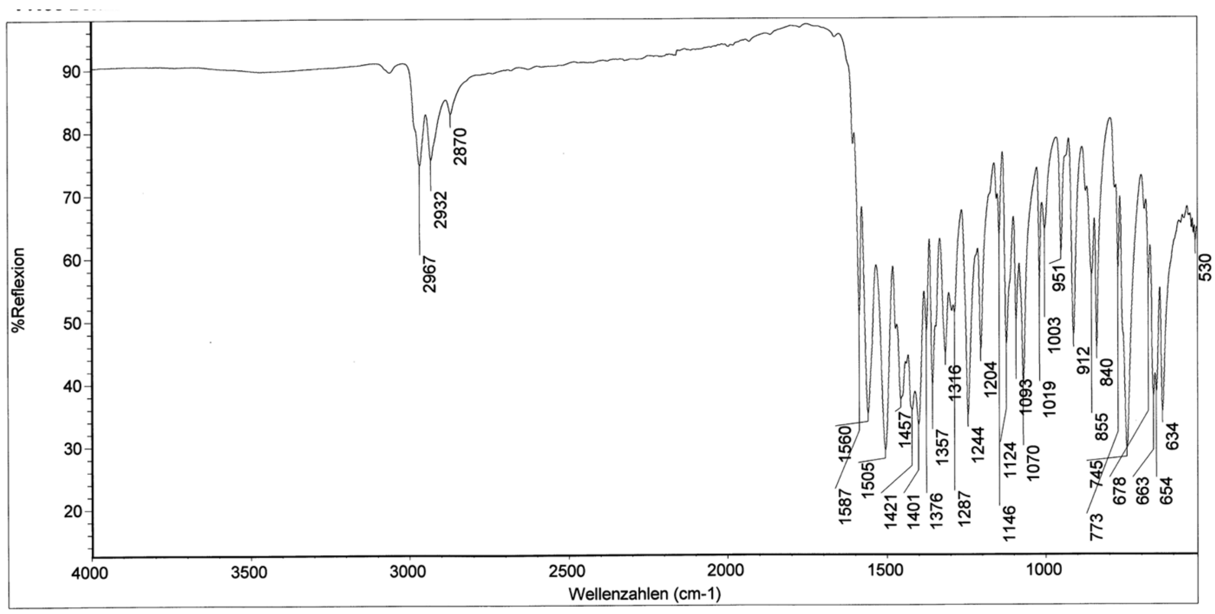

Figure S39: IR (ATR) spectrum of [Tb⊂{Au_3(L1^{ethyl})$_3$}] (**8**).

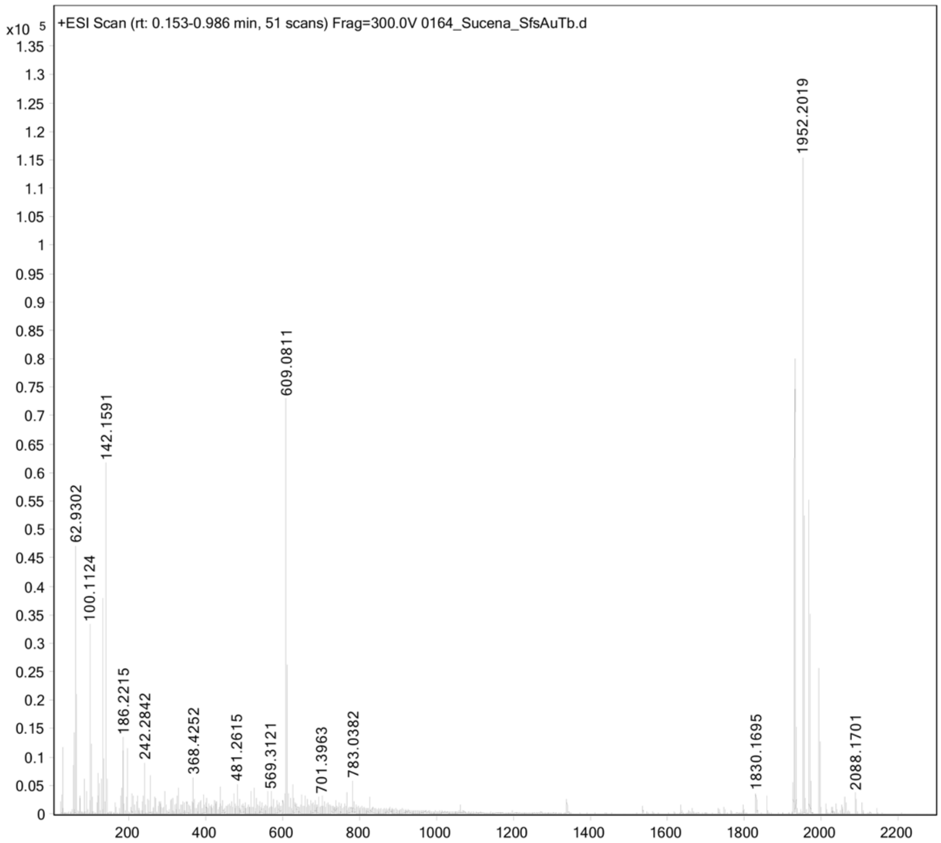

Figure S40: ESI+ MS spectrum of [Tb⊂{Au_3(L1^{ethyl})$_3$}] (**8**).

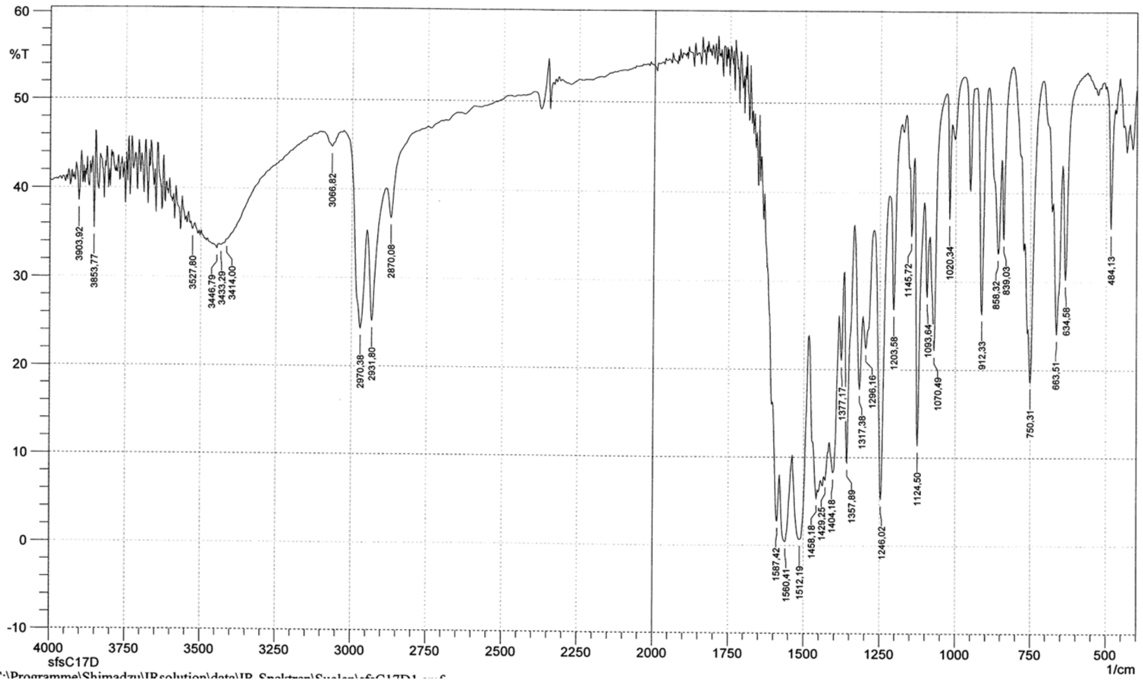

Figure S41: IR (KBr) spectrum of [Dy⊂{Au_3($L1^{ethyl}$)$_3$}] (**9**).

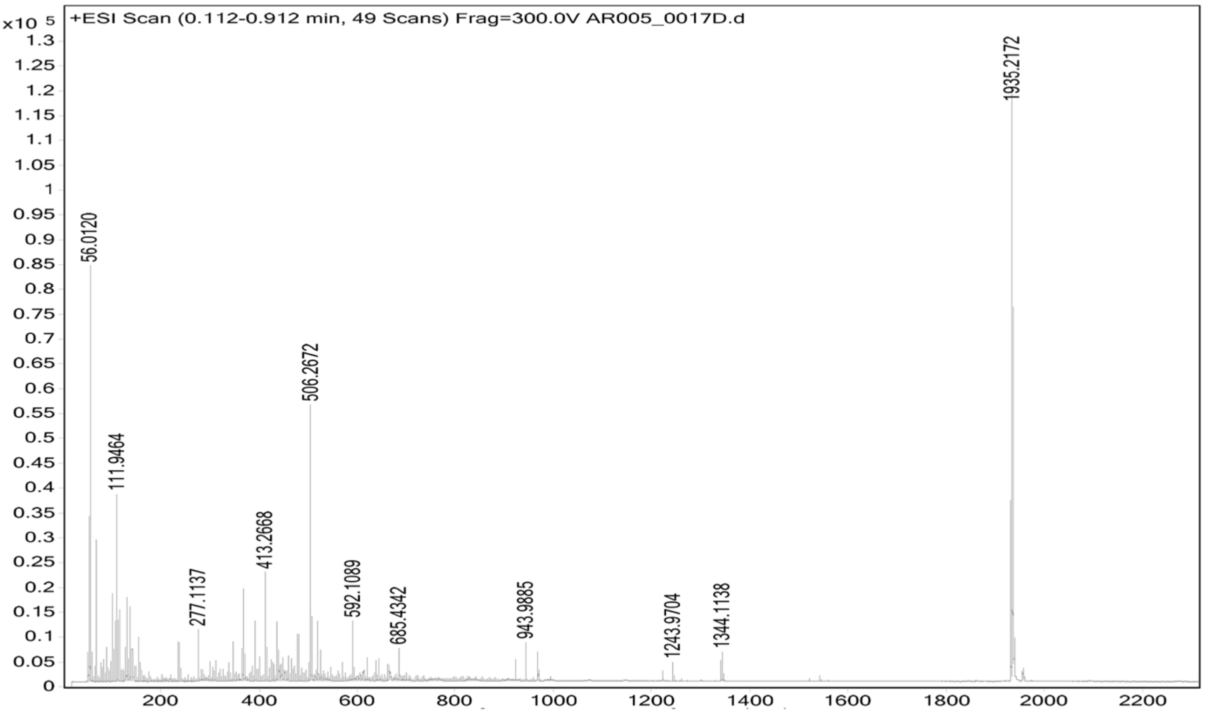

Figure S42: ESI+ MS spectrum of [Dy⊂{Au_3($L1^{ethyl}$)$_3$}] (**9**).

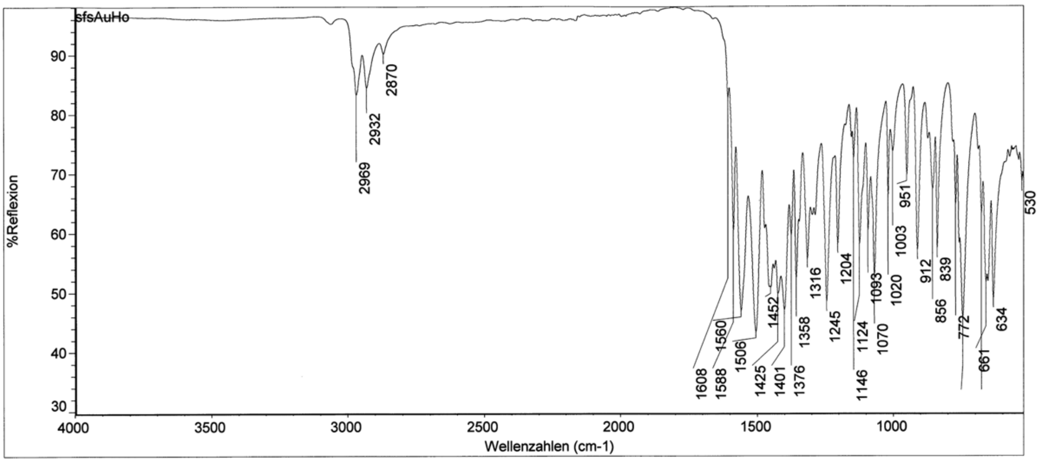

Figure S43: IR (ATR) spectrum of [Ho⊂{$Au_3(L1^{ethyl})_3$}] (**10**).

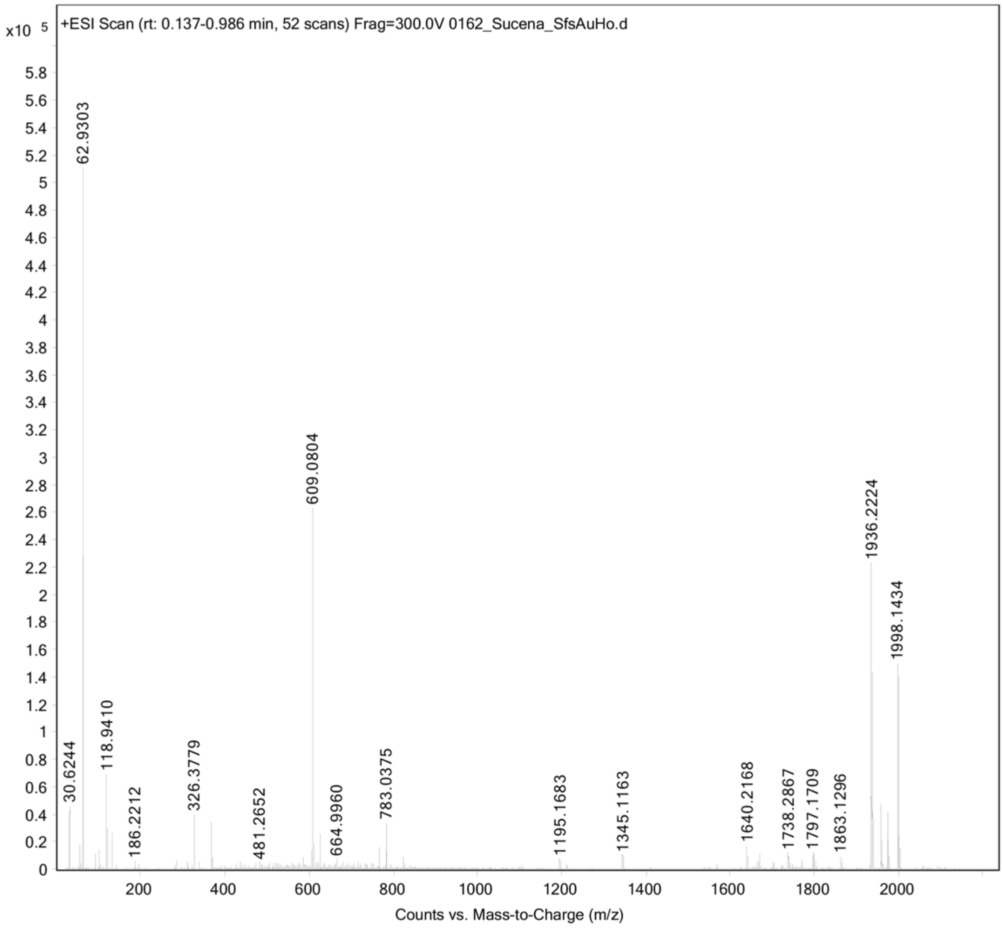

Figure S44: ESI+ MS spectrum of [Ho⊂{$Au_3(L1^{ethyl})_3$}] (**10**).

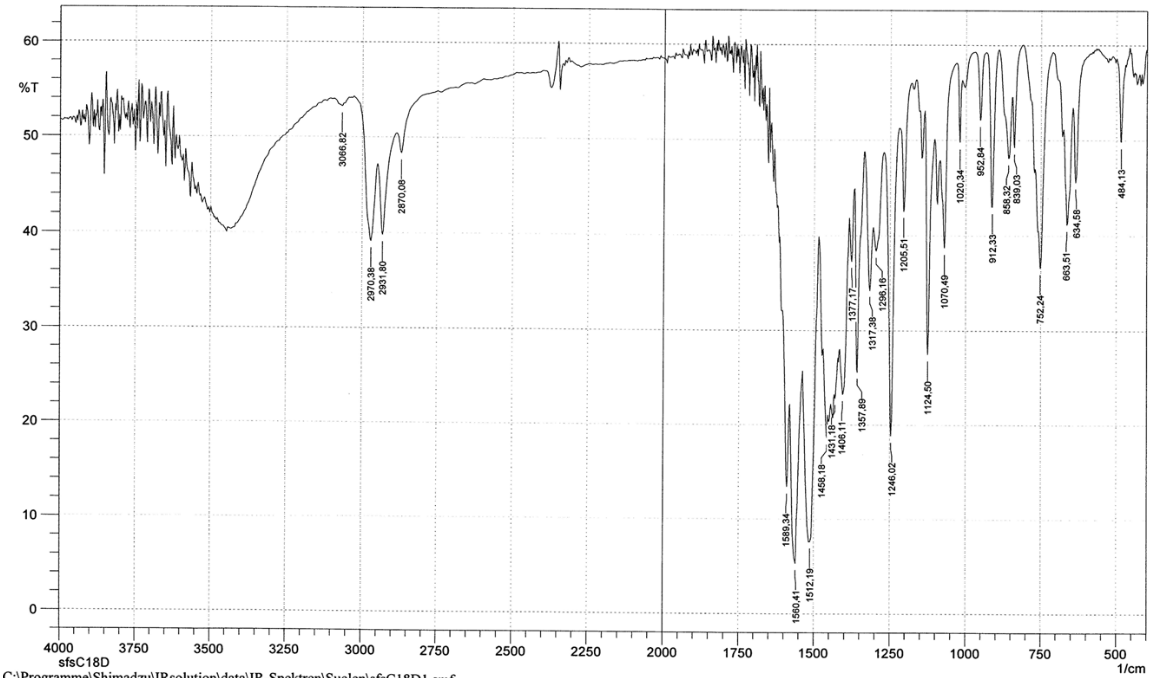

Figure S45: IR (KBr) spectrum of [Er⊂{Au_3(L1^{ethyl})$_3$}] (**11**).

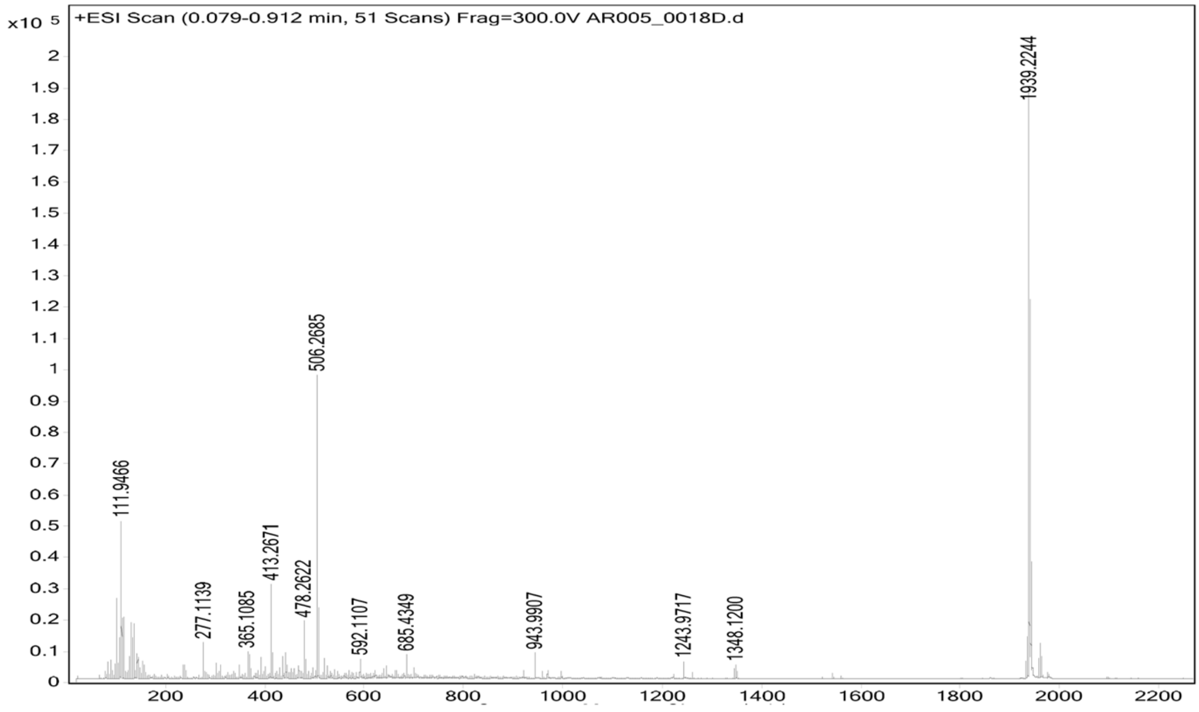

Figure S46: ESI+ MS spectrum of [Er⊂{Au_3(L1^{ethyl})$_3$}] (**11**).

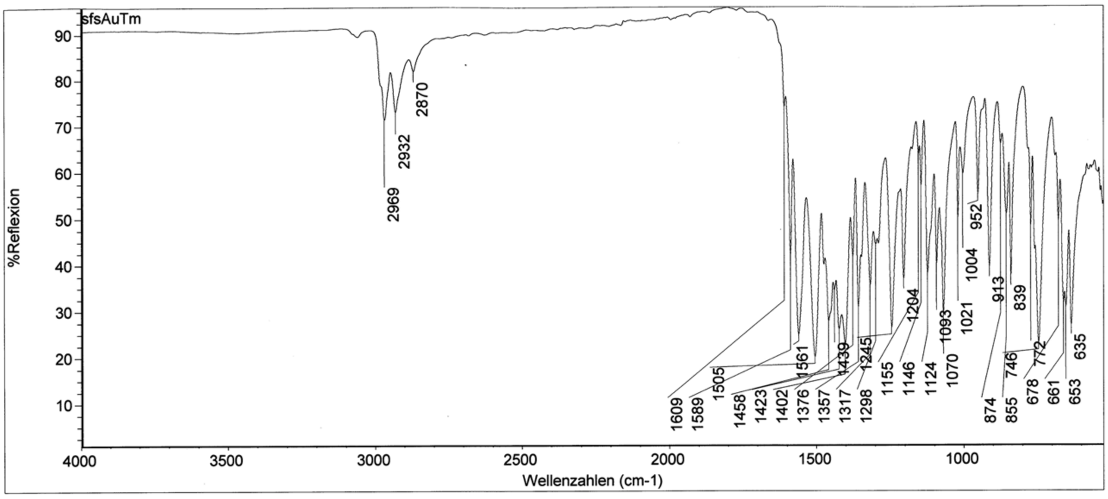

Figure S47: IR (ATR) spectrum of [Tm⊂{Au_3(L1^{ethyl})$_3$}] (**12**).

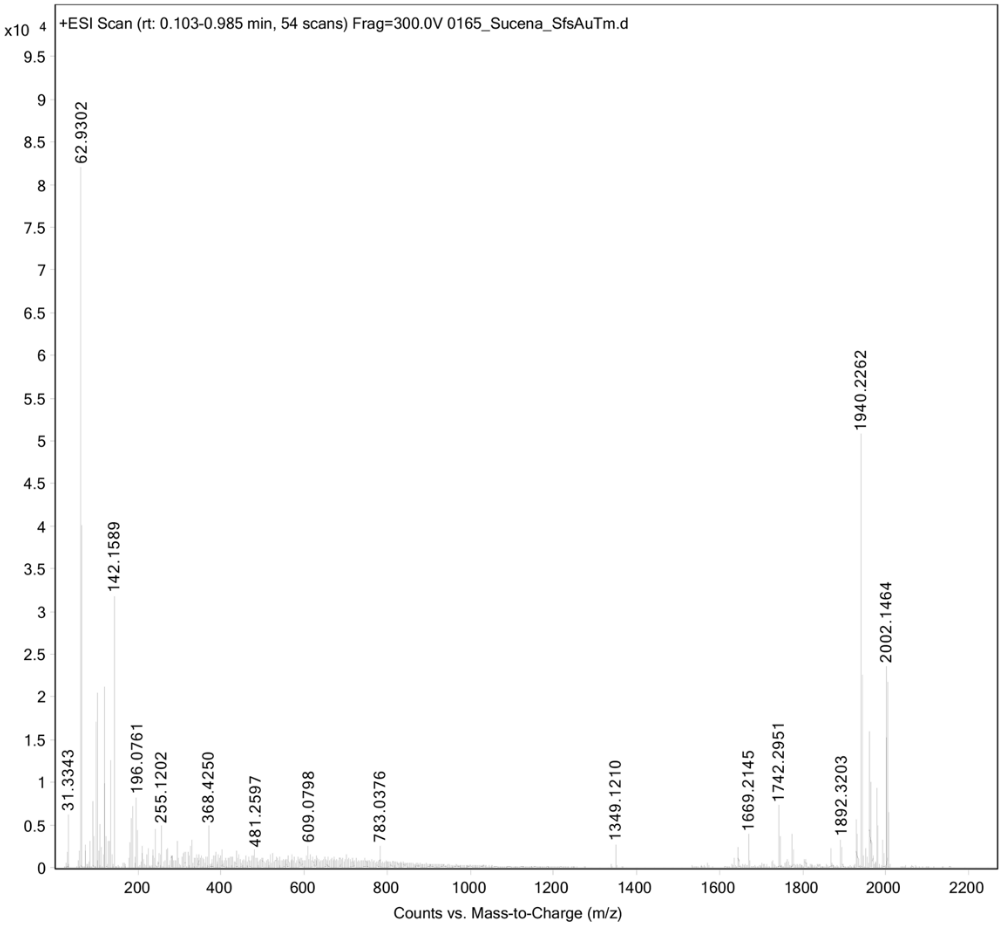

Figure S48: ESI+ MS spectrum of [Tm⊂{Au_3(L1^{ethyl})$_3$}] (**12**).

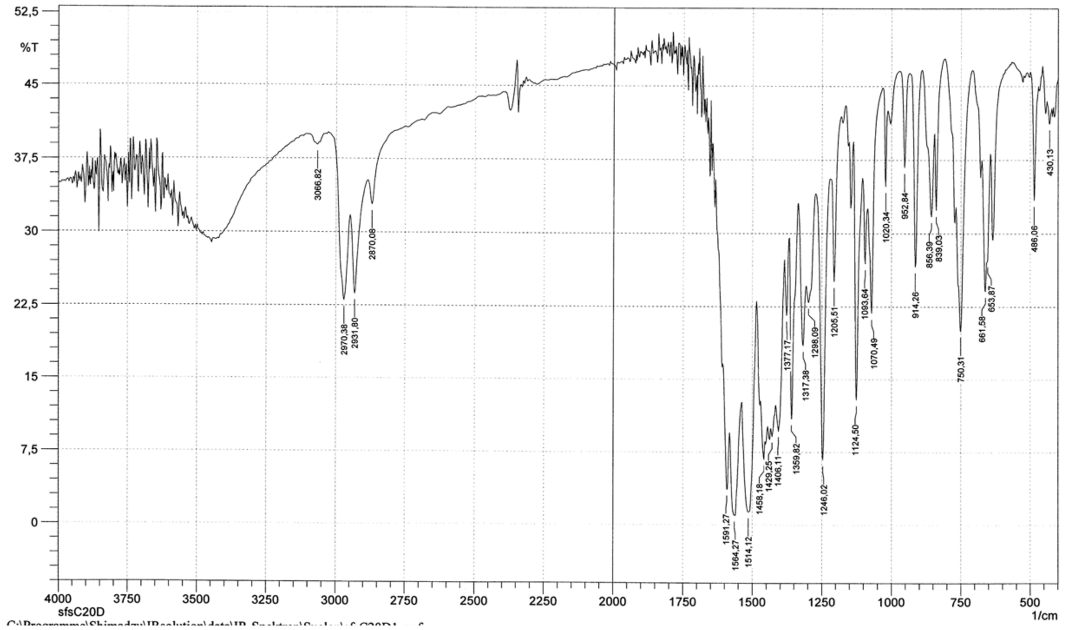

Figure S49: IR (KBr) spectrum of [Yb⊂{Au_3($L1^{ethyl}$)$_3$}] (**13**).

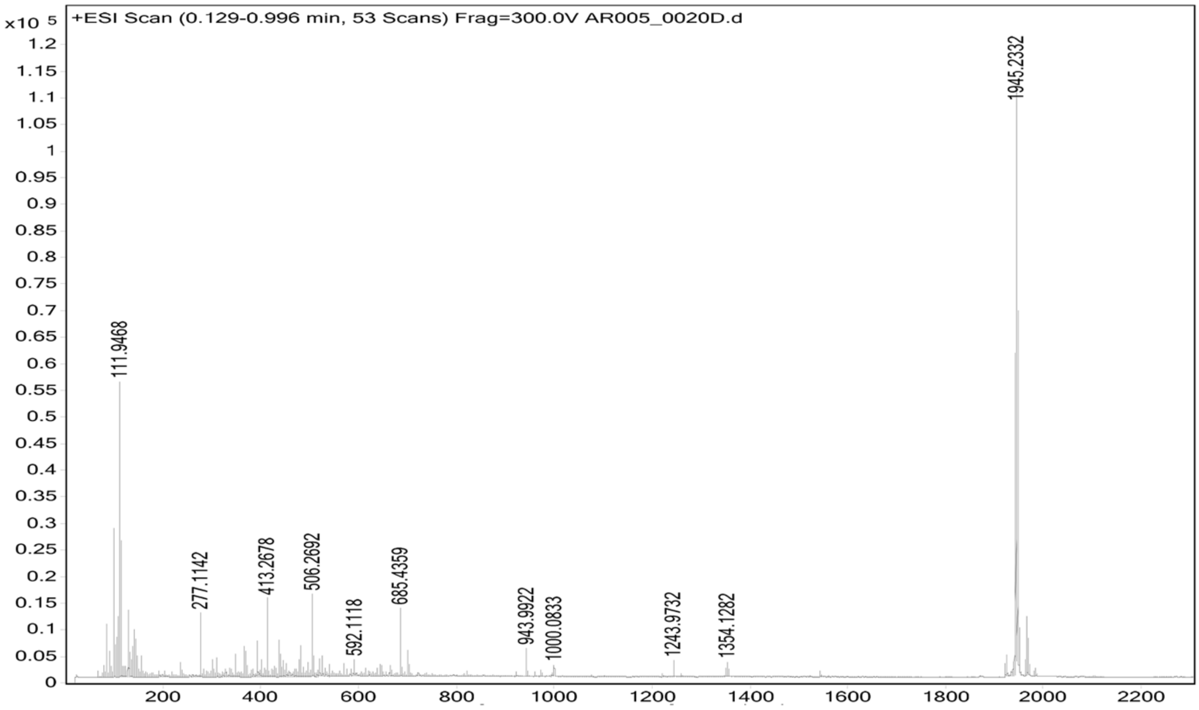

Figure S50: ESI+ MS spectrum of [Yb⊂{Au_3($L1^{ethyl}$)$_3$}] (**13**).

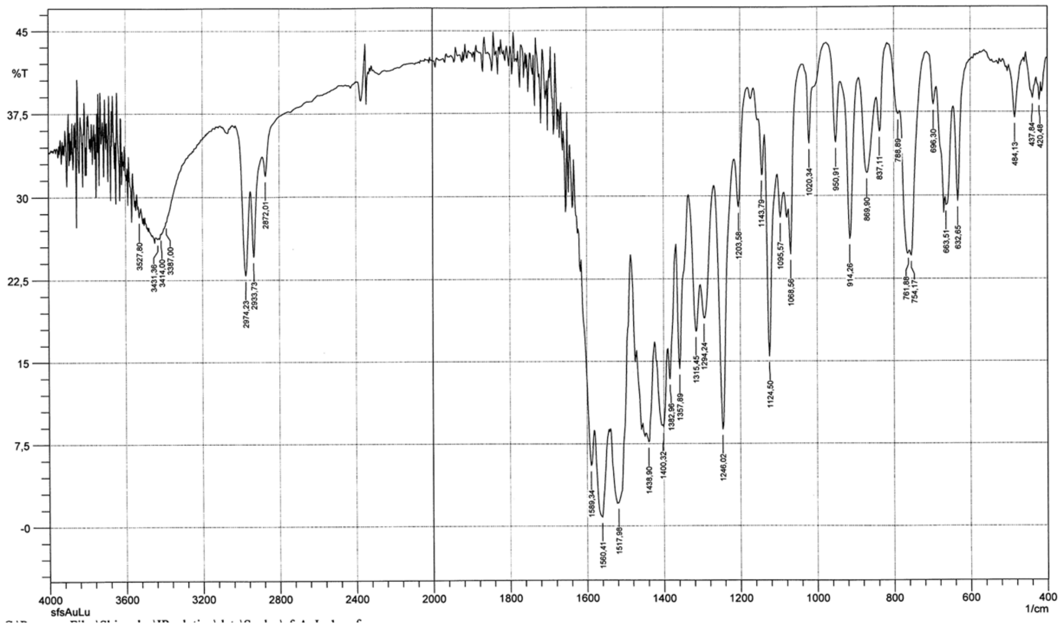

Figure S51: IR (KBr) spectrum of [Lu⊂{Au_3($L1^{ethyl}$)$_3$}] (**14**).

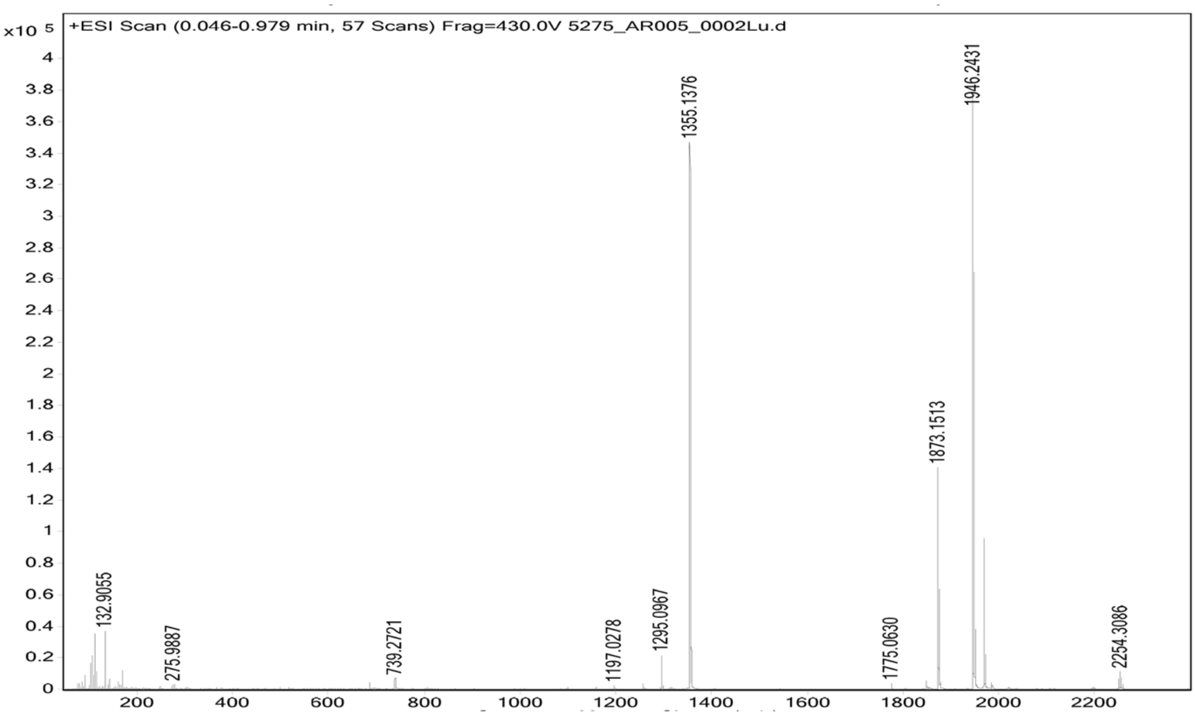

Figure S52: ESI+ MS spectrum of [Lu⊂{Au_3($L1^{ethyl}$)$_3$}] (**14**).

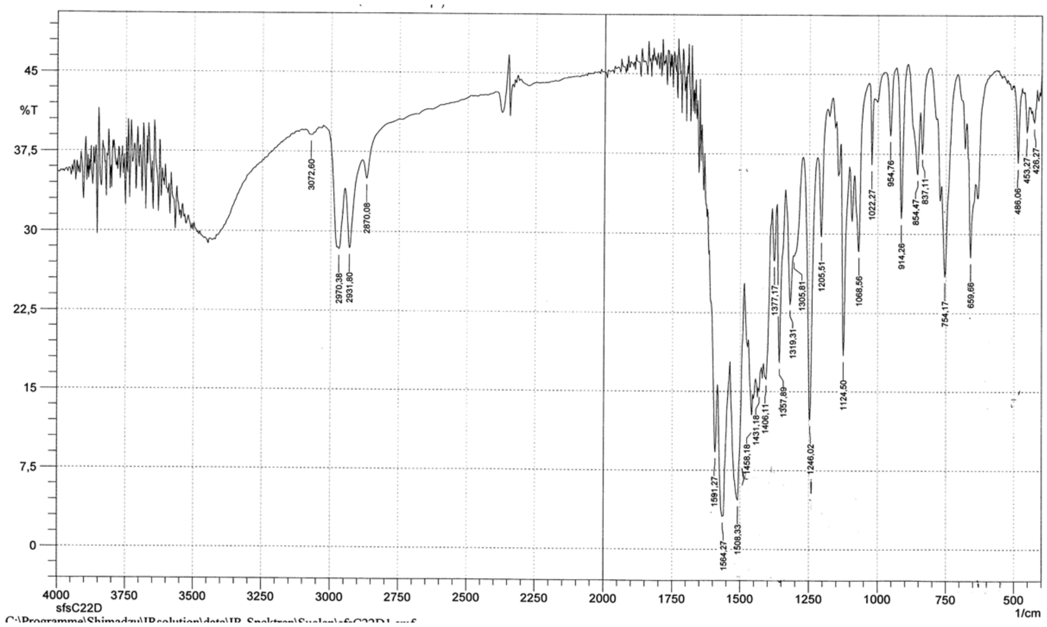

Figure S53: IR (KBr) spectrum of [Sc⊂{$Au_3(L1^{ethyl})_3$}] (**15**).

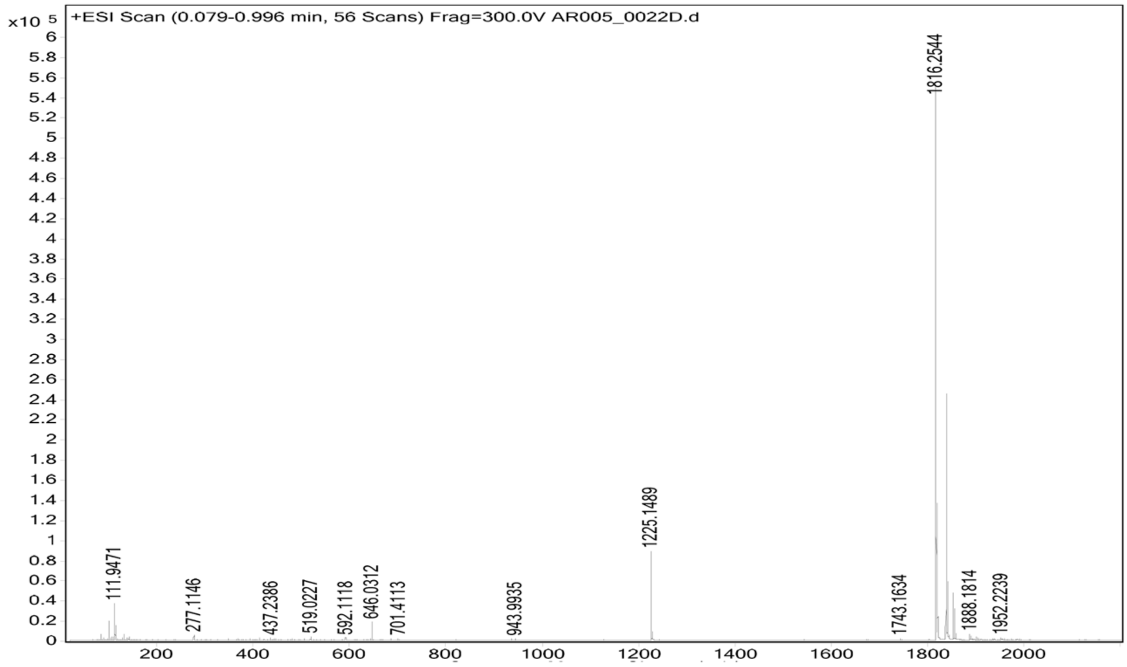

Figure S54: ESI+ MS spectrum of [Sc⊂{$Au_3(L1^{ethyl})_3$}] (**15**).

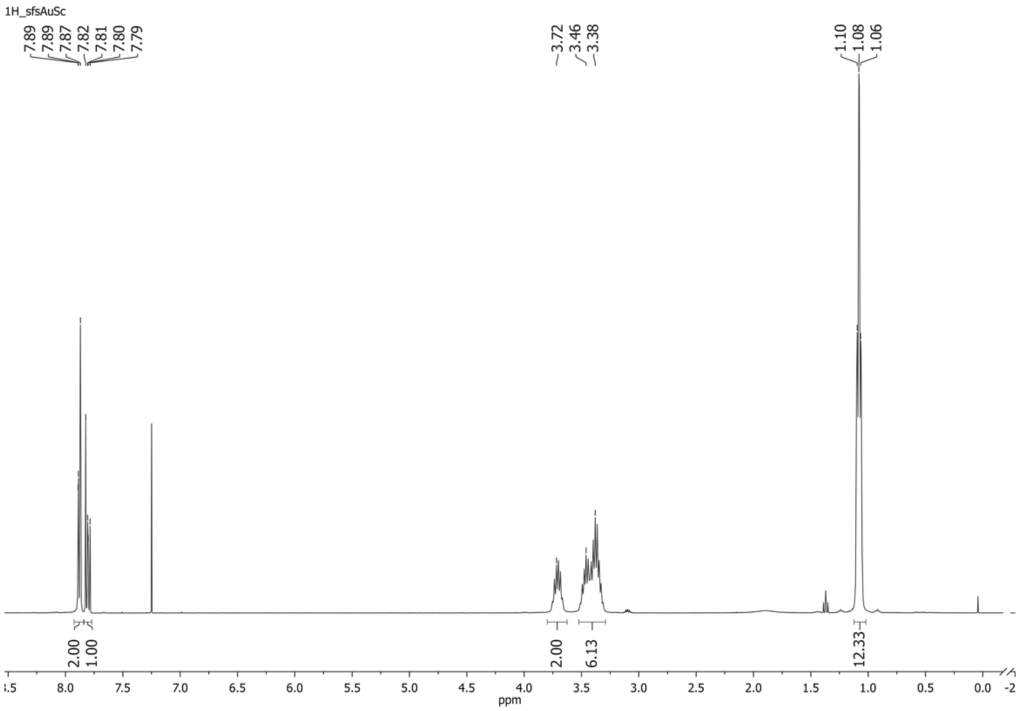

Figure S55: ^{1}H NMR spectrum of [Sc⊂{Au_3(L1^{ethyl})$_3$}] (**15**) in $CDCl_3$.

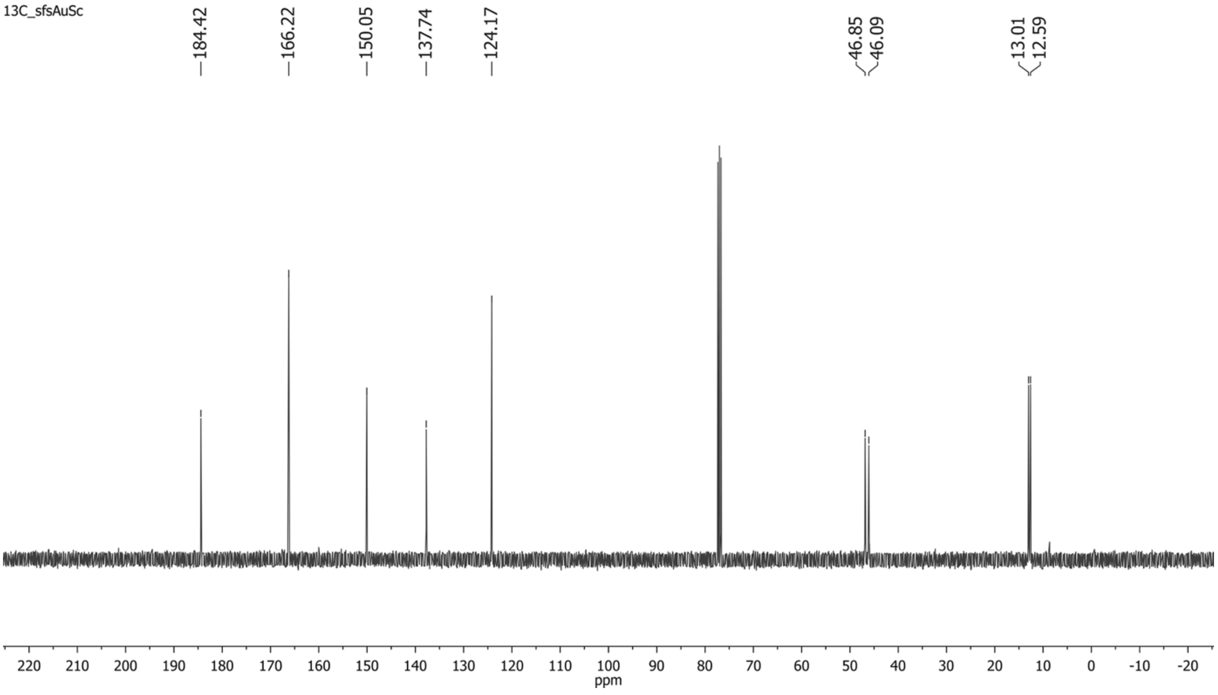

Figure S56: ^{13}C NMR spectrum of [Sc⊂{Au_3(L1^{ethyl})$_3$}] (**15**) in $CDCl_3$

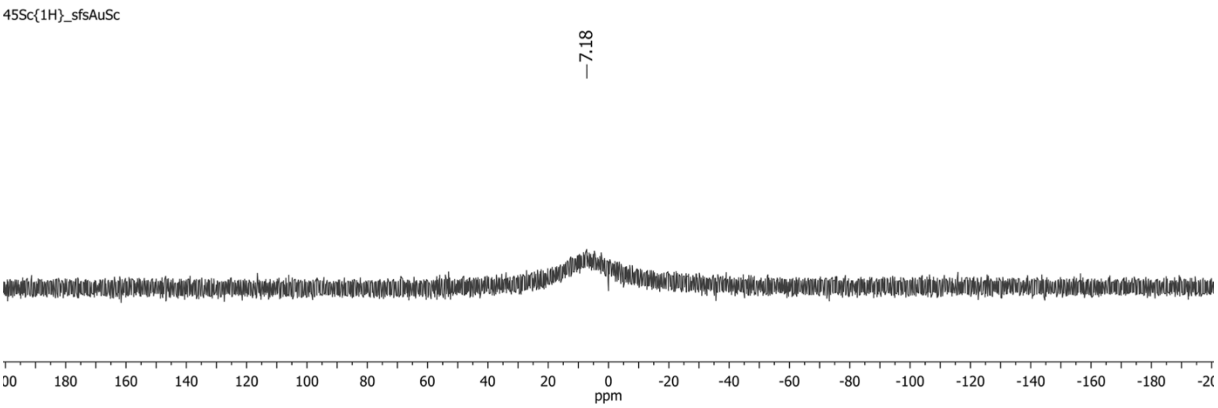

Figure S57: ^{45}Sc NMR spectrum of [Sc⊂{Au_3(L1^{ethyl})$_3$}] (**15**) in CD_2Cl_2.

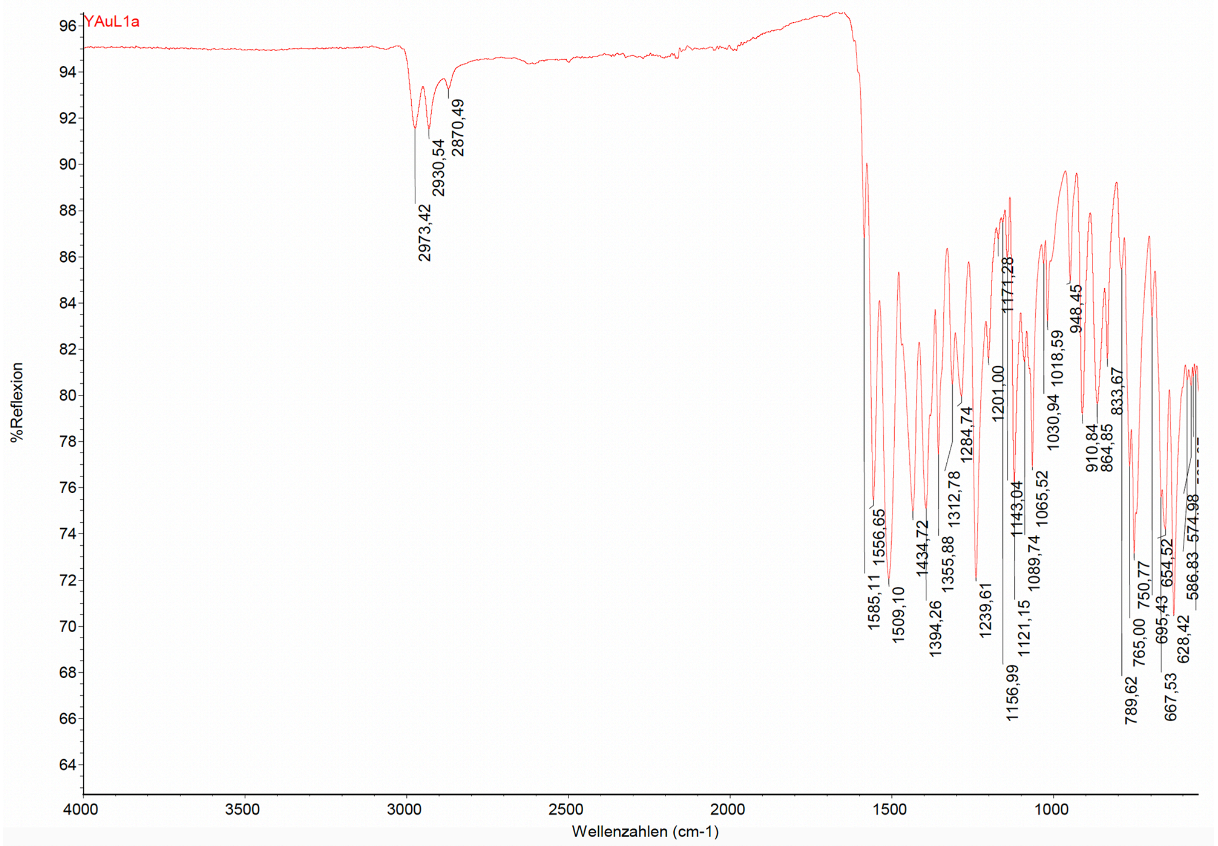

Figure S58: IR (ATR) spectrum of [Y⊂{Au_3(L1^{ethyl})$_3$}] (**16**).

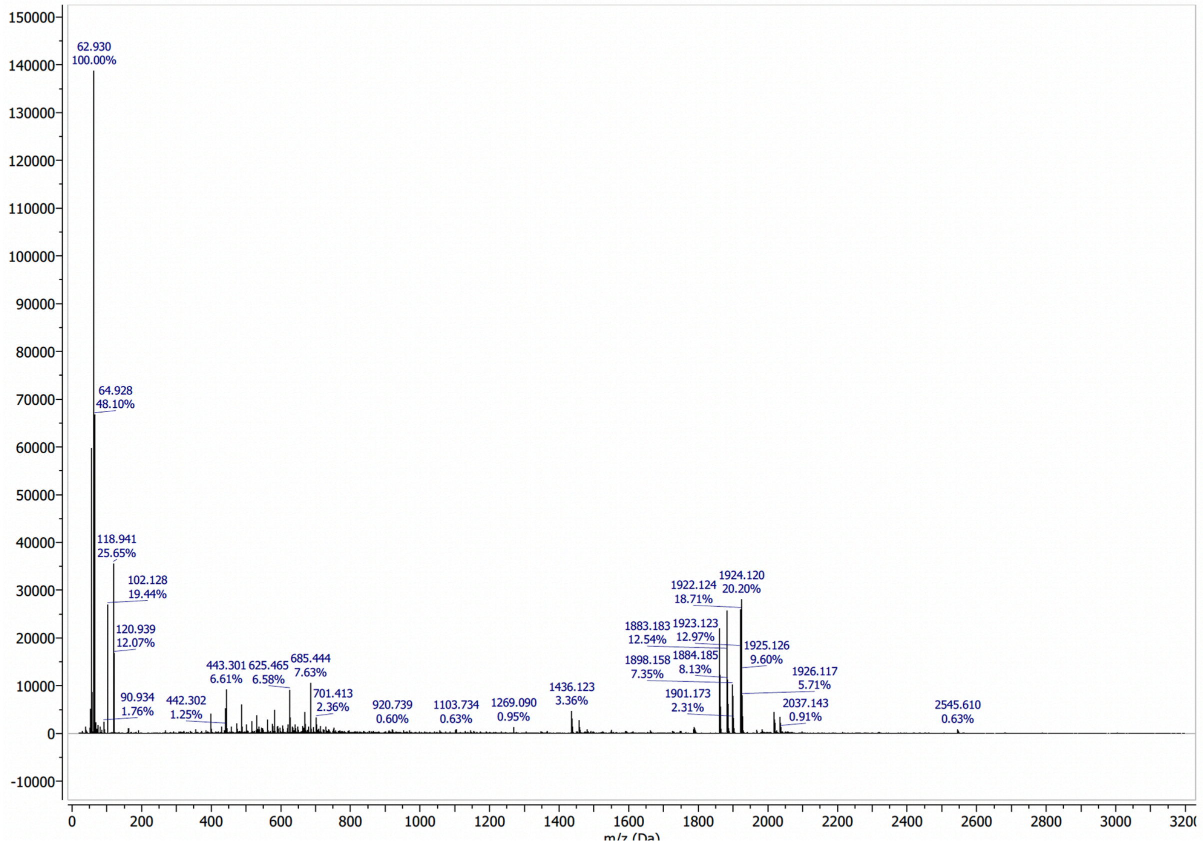

Figure S59: ESI MS spectrum of [Y⊂{Au_3(L1^{ethyl})$_3$}] (**16**).

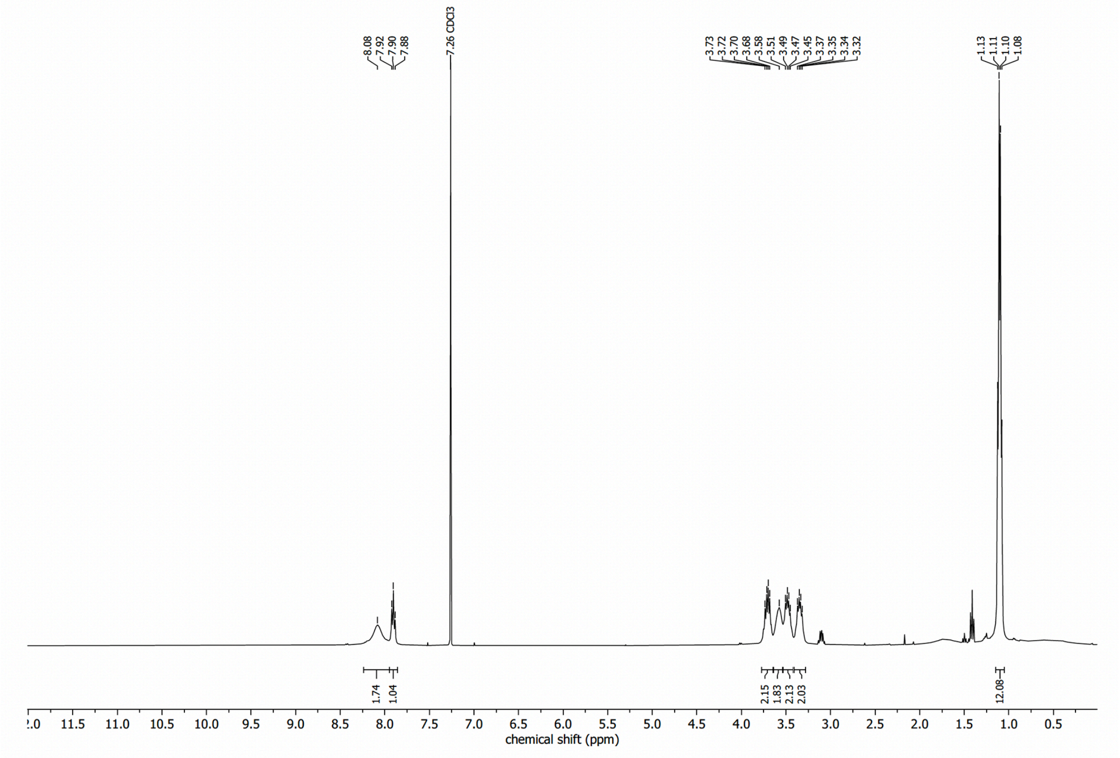

Figure S60: ^{1}H NMR spectrum of [Y⊂{Au_3(L1^{ethyl})$_3$}] (**16**) in $CDCl_3$.

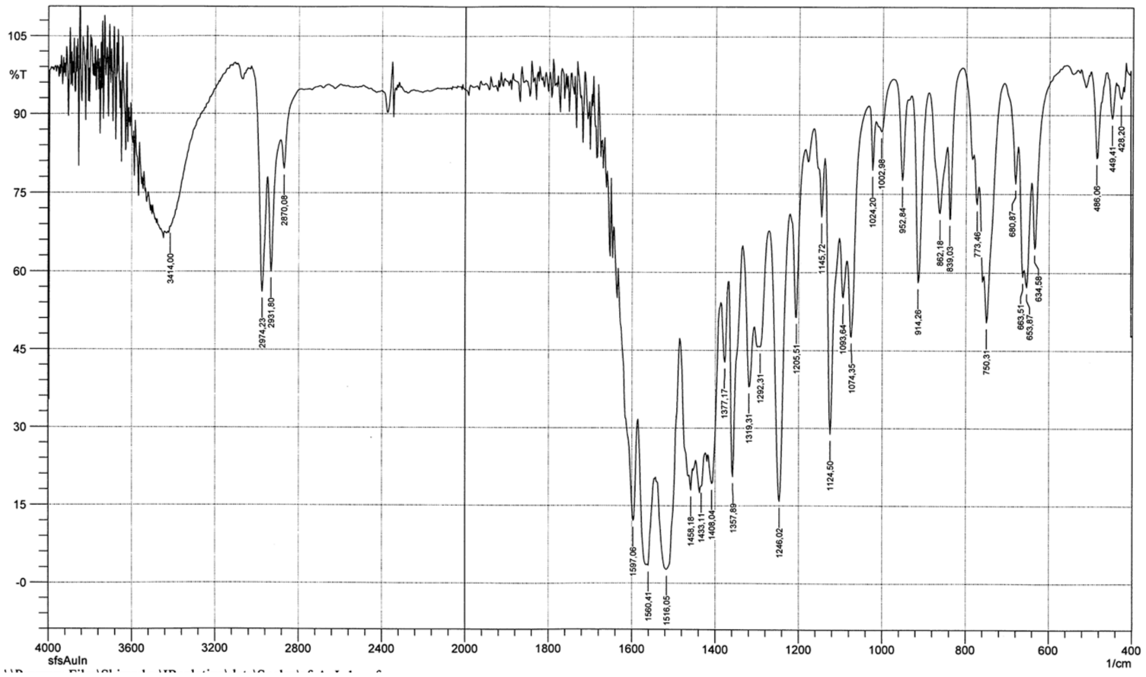

Figure S61: IR (KBr) spectrum of [In⊂{Au3(L1ethyl)3}] (17).

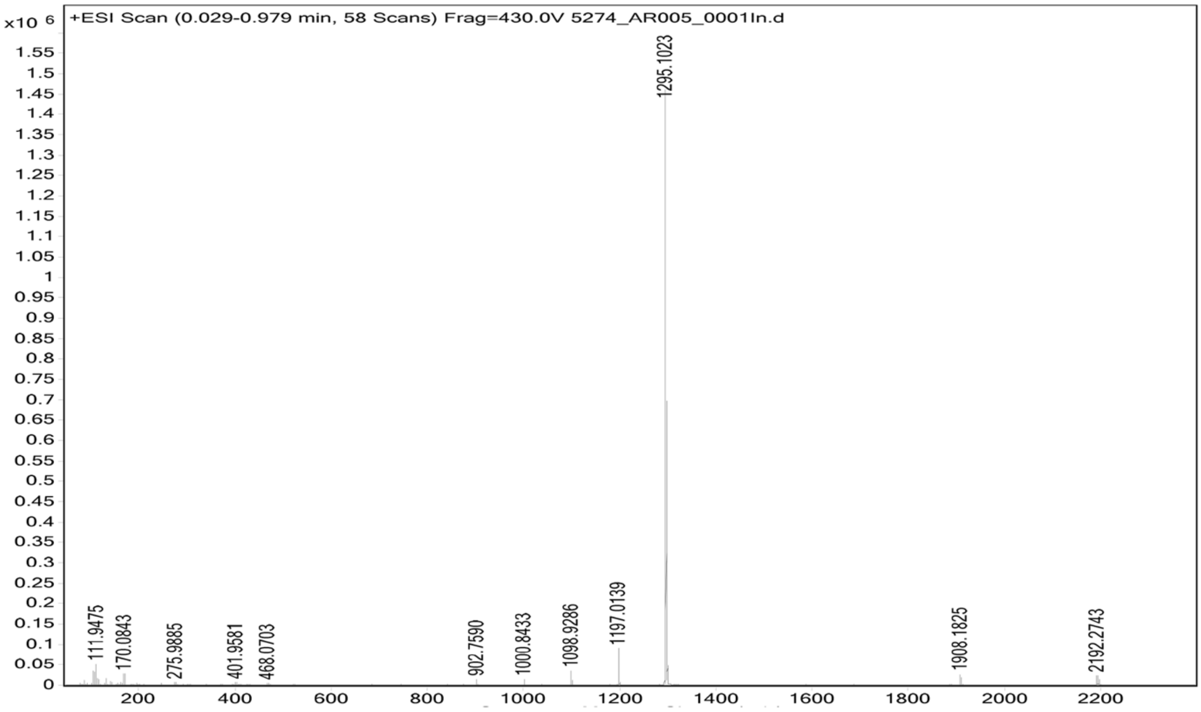

Figure S62: ESI+ MS spectrum of [In⊂{Au_3(L1^{ethyl})$_3$}] (**17**).

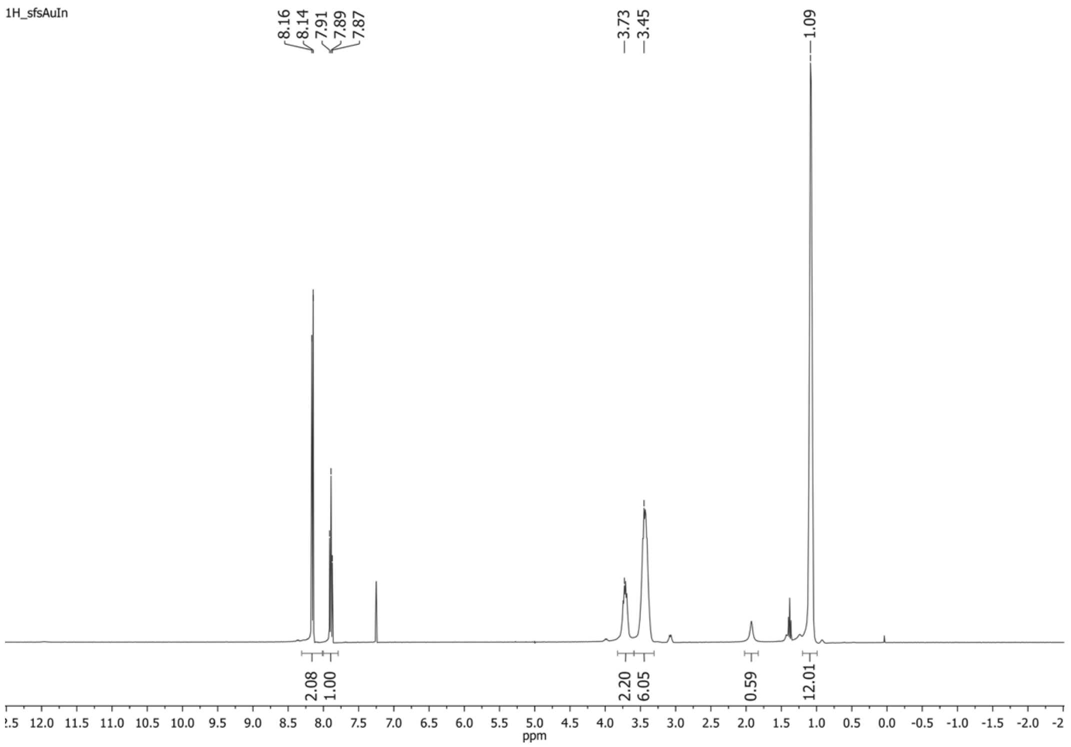

Figure S63: ^{1}H NMR spectrum of [In⊂{Au$_3$(L1^{ethyl})$_3$}] (**17**) in $CDCl_3$.

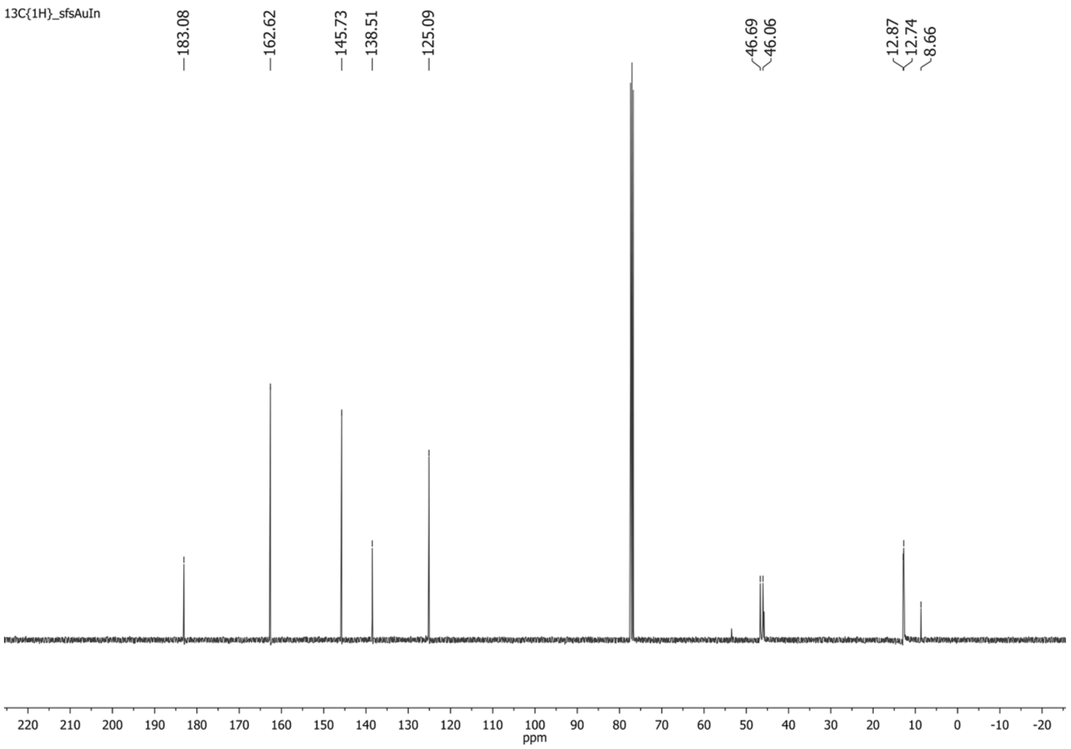

Figure S64: ^{13}C NMR spectrum of [In⊂{Au$_3$(L1^{ethyl})$_3$}] (**17**) in $CDCl_3$.

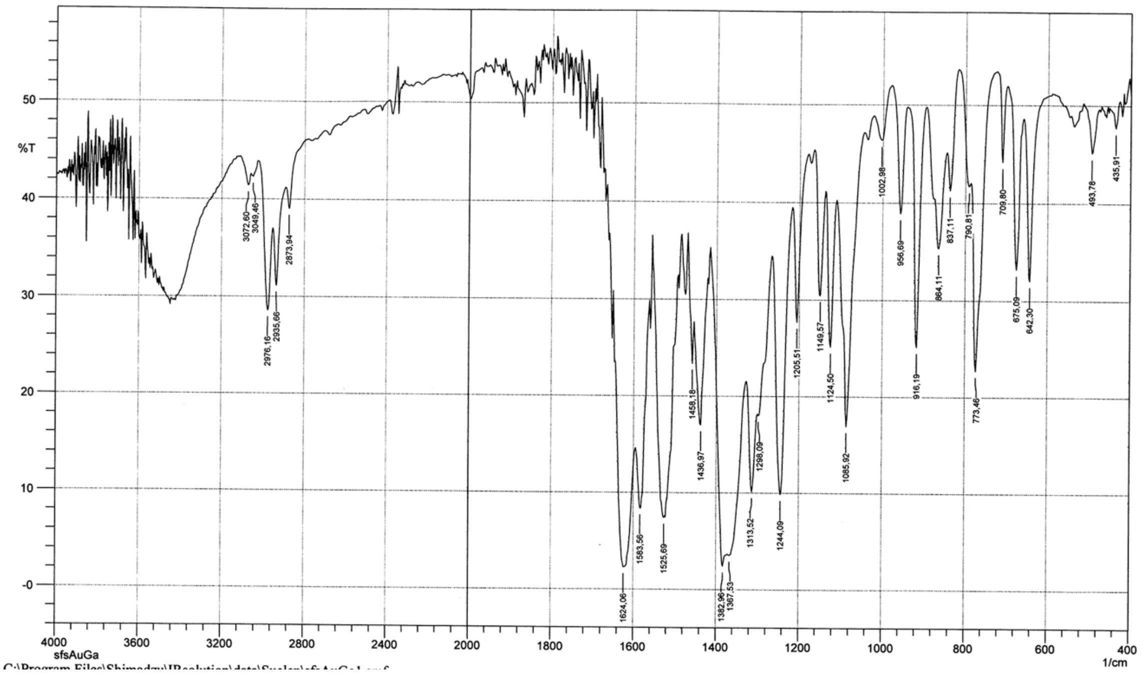

Figure S65: IR (KBr) spectrum of [Ga⊂{Au$_2$(L1^{ethyl})$_2$}](NO$_3$) (**18a**).

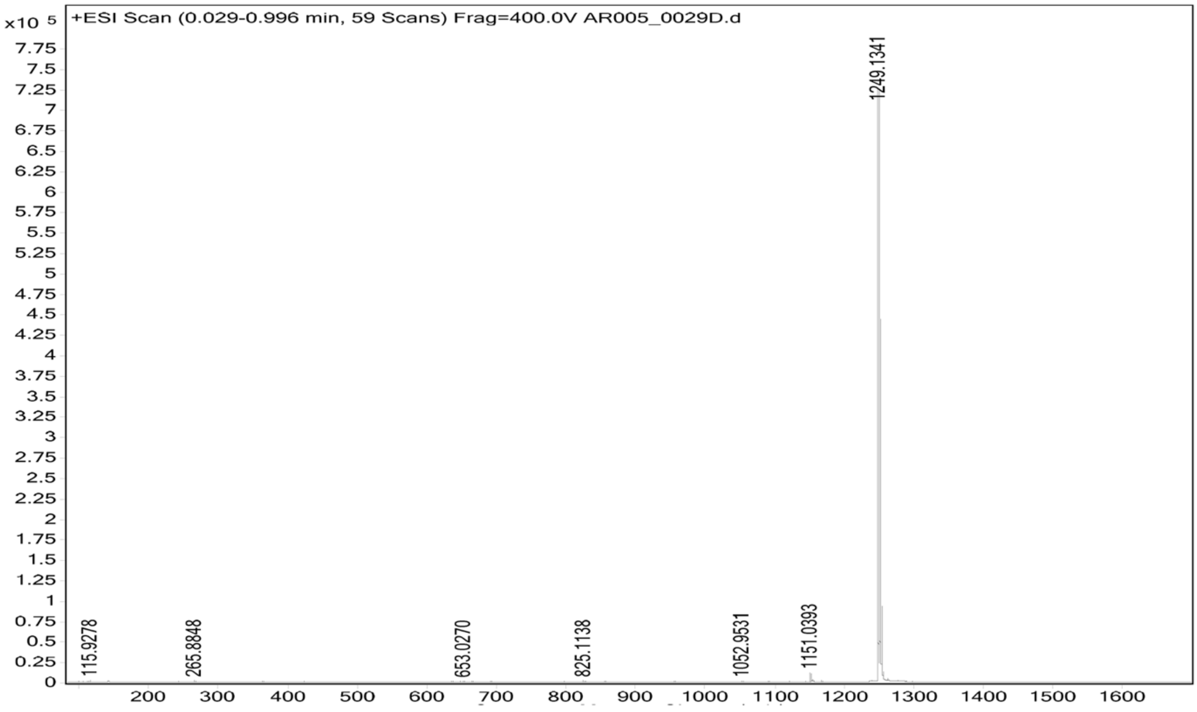

Figure S66: ESI+ MS spectrum of [Ga⊂{Au$_2$(L1^{ethyl})$_2$}](NO$_3$) (**18a**).

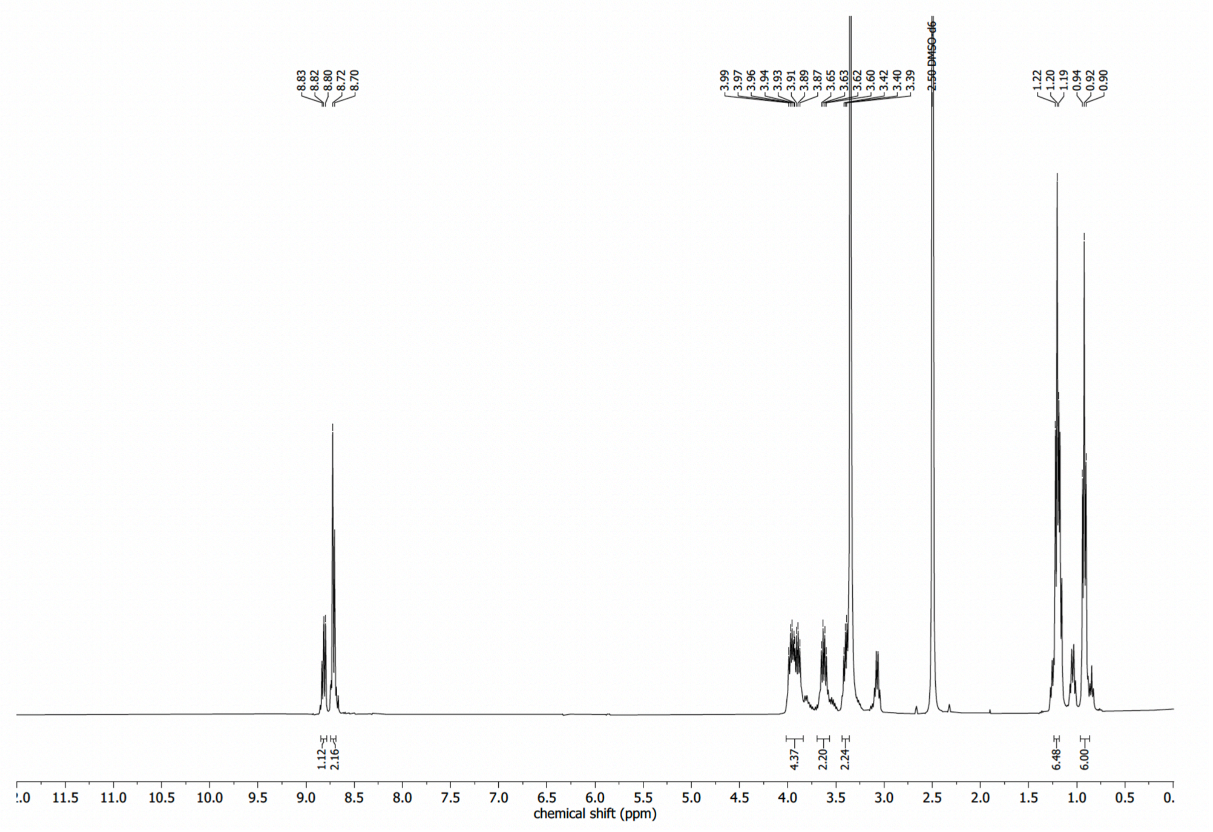

Figure S67: ^{1}H NMR spectrum of [Ga⊂{Au_2(L1^{ethyl})$_2$}](NO_3) (**18a**) in DMSO-D_6.

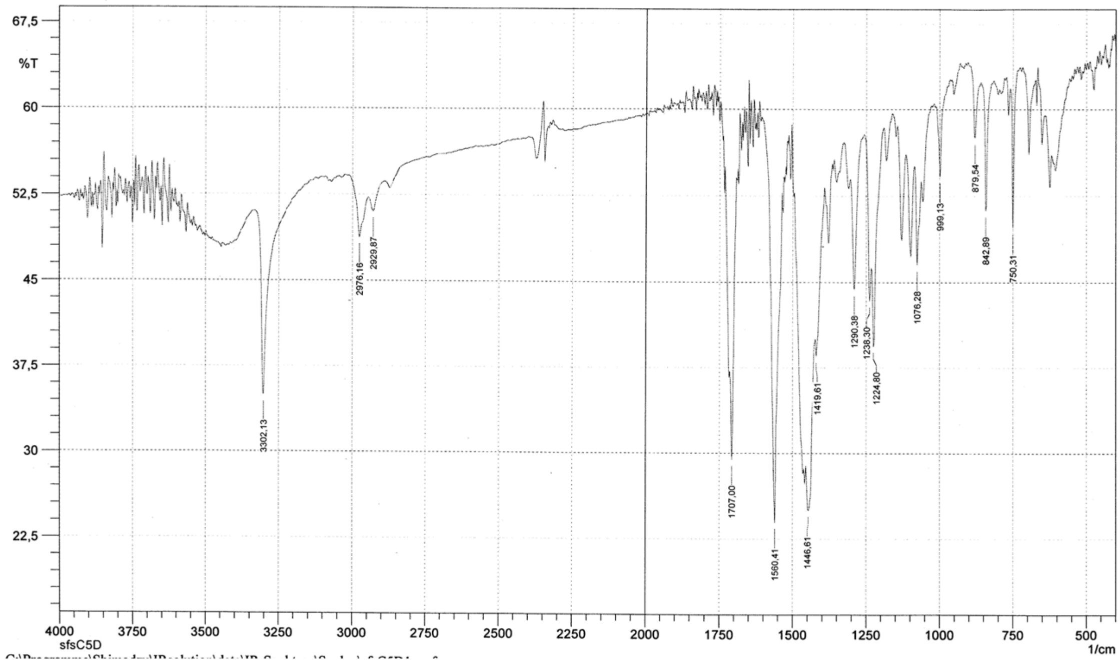

Figure S68: IR (KBr) spectrum of [{AuCl}$_2$(H_2L1^{ethyl})] (**19**).

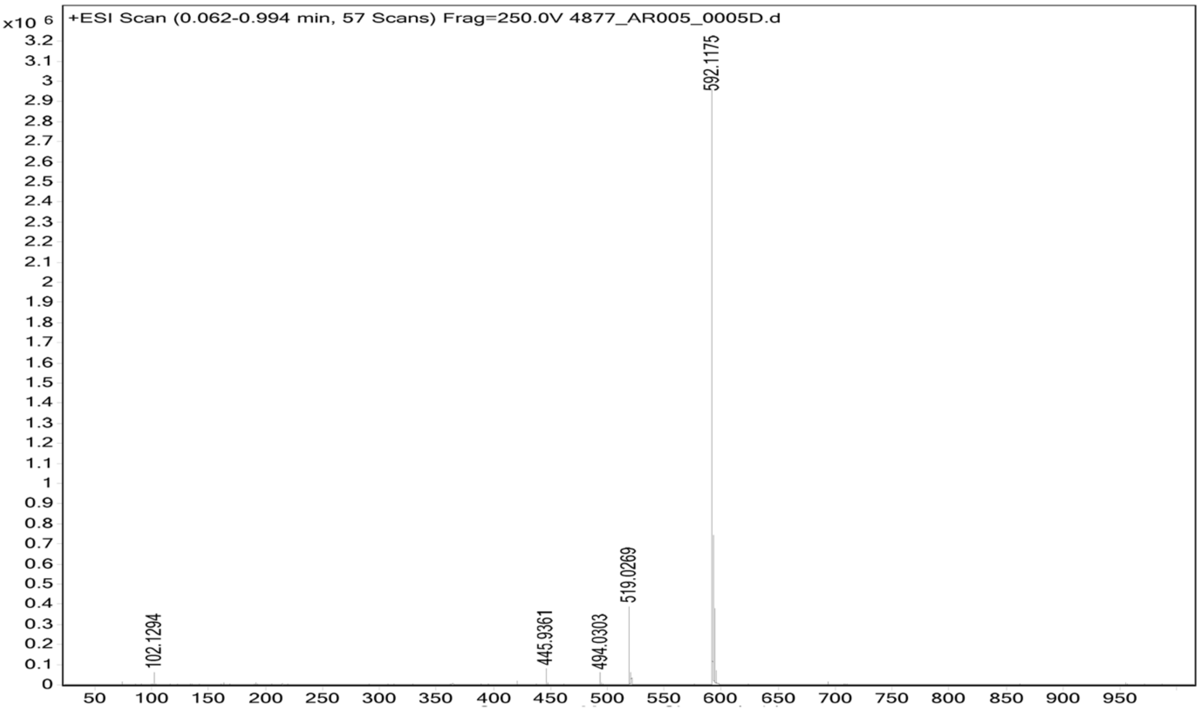

Figure S69: ESI MS spectrum of [{AuCl}$_2$(H$_2$L1^{ethyl})] (**19**).

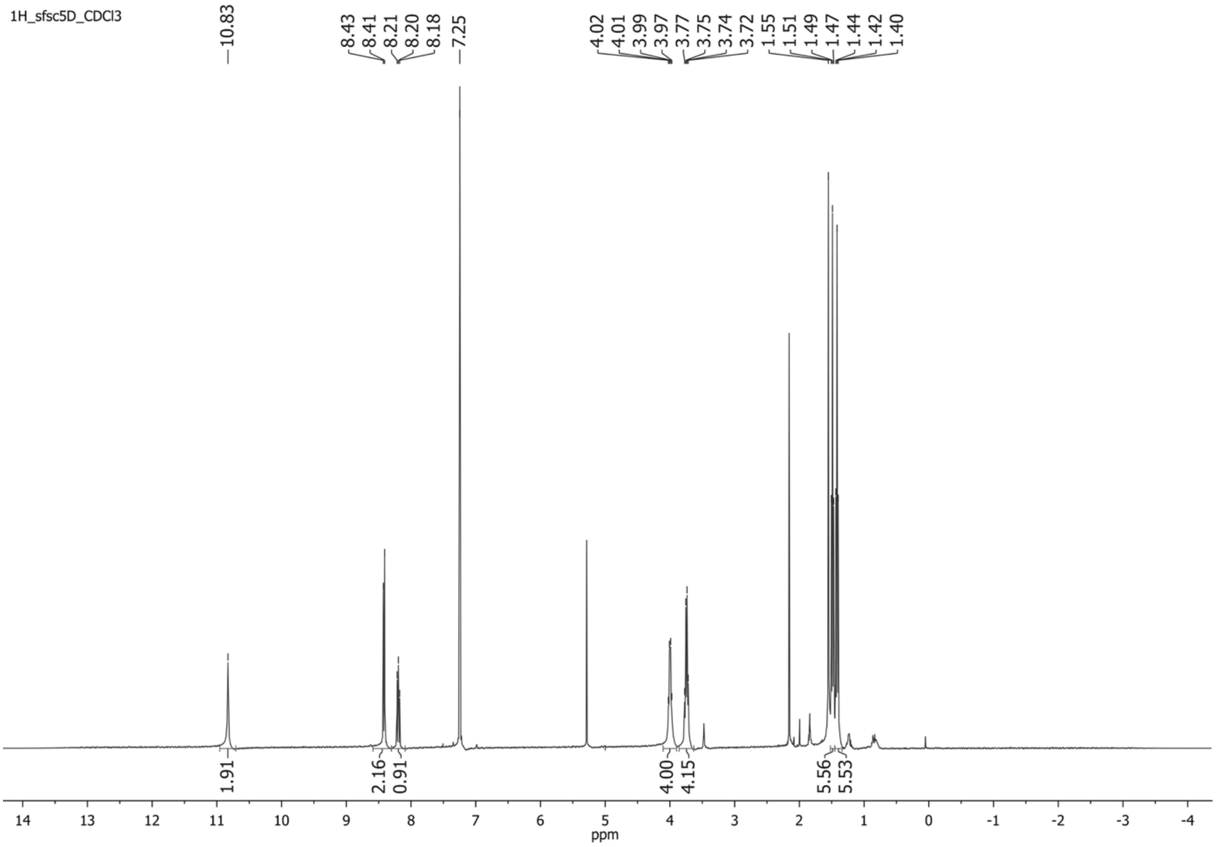

Figure S70: ^{1}H NMR spectrum of [{AuCl}$_2$(H$_2$L1^{ethyl})] (**19**). in CDCl$_3$.

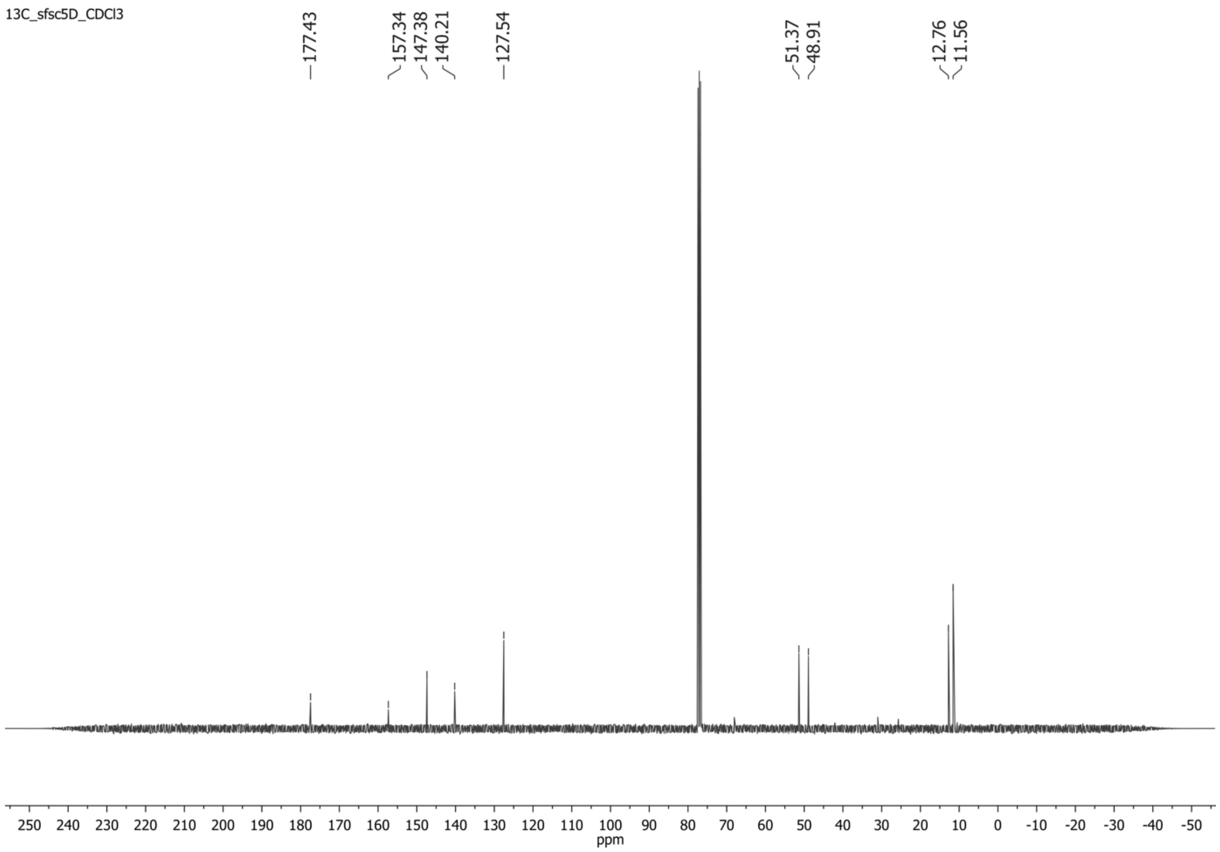

Figure S71: ^{13}C NMR spectrum of [{AuCl}$_2$(H$_2$L1^{ethyl})] (**19**). in CDCl$_3$.

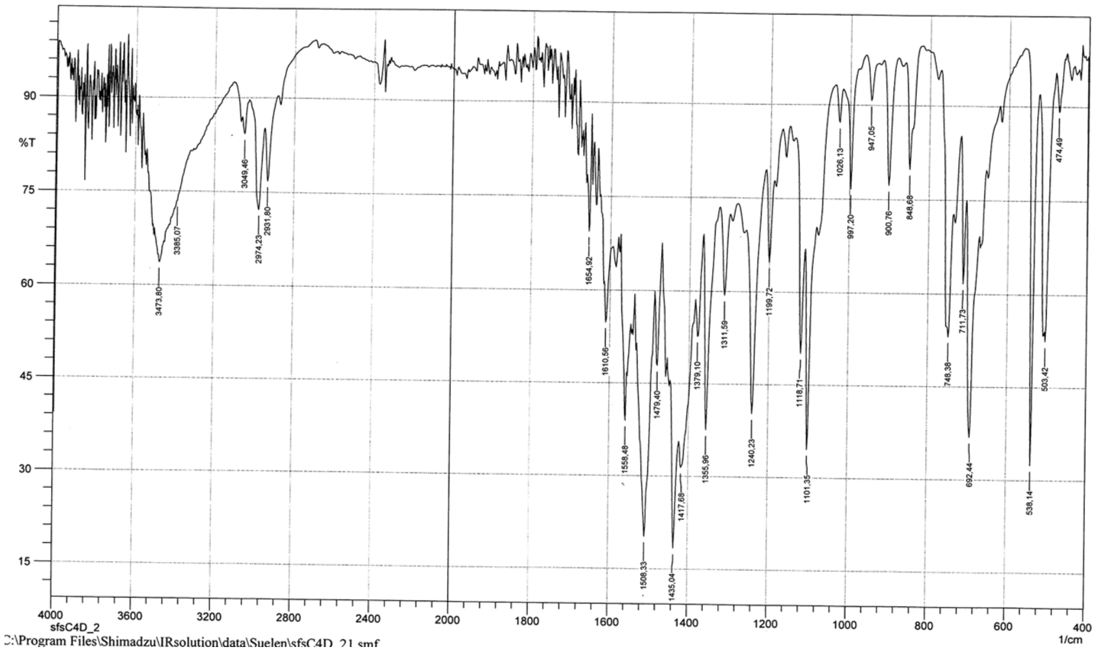

Figure S72: IR (KBr) spectrum of [{Au(PPh$_3$}$_2$(L1^{ethyl})] (**20**).

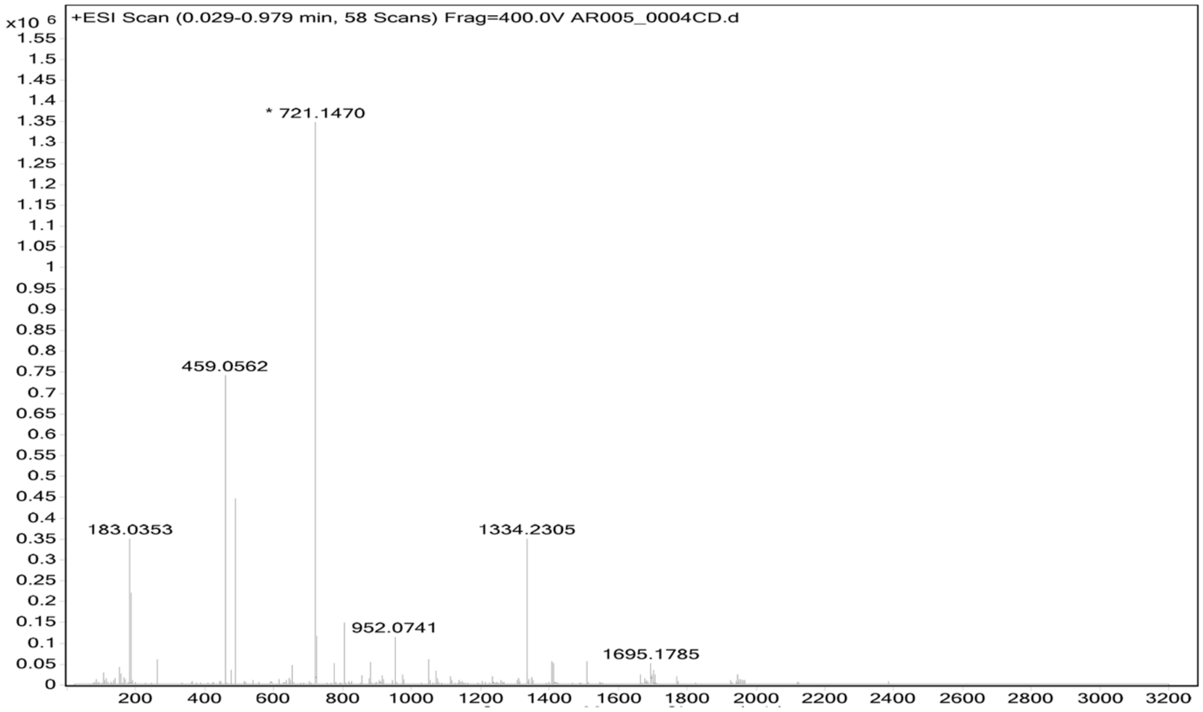

Figure S73: ESI+ MS spectrum of [{Au(PPh$_3$}$_2$(L1^{ethyl})] (**20**).

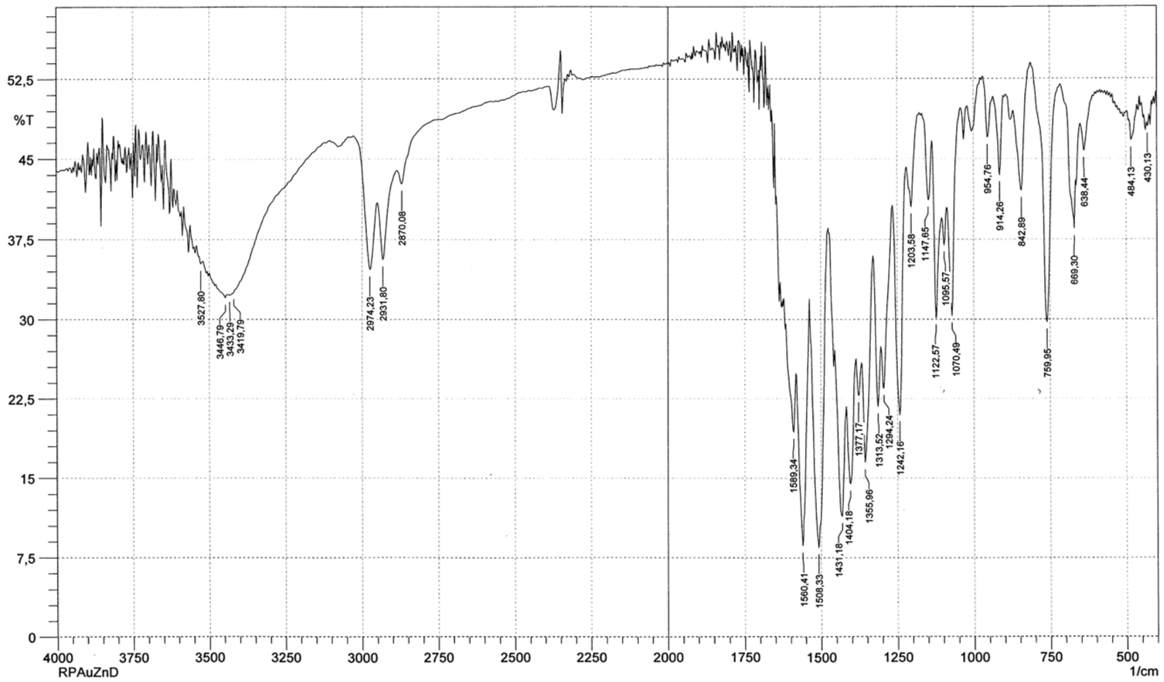

Figure S74: IR (KBr) spectrum of [Zn⊂{Au$_2$(L1^{ethyl})$_2$}] (**21**).

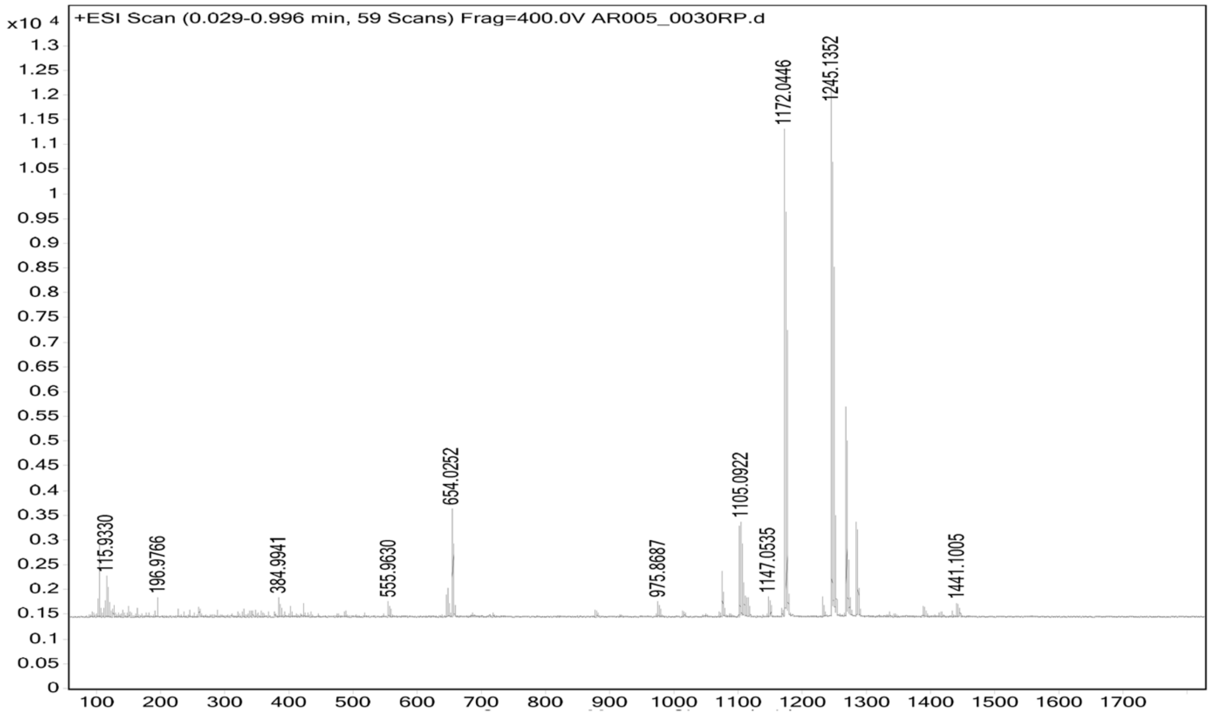

Figure S75: ESI+ MS spectrum of [Zn⊂{Au_2(L1^{ethyl})$_2$}] (**21**).
